# Development and evaluation of a values-based anti-doping education program for university sport in Japan: the UNIVAS clean sports intervention

**DOI:** 10.3389/fspor.2026.1835205

**Published:** 2026-06-22

**Authors:** Yuka Murofushi, Yusuke Okada, Eishin Teraoka, Kosuke Suzuki, Yoshinori Okade, Hisashi Naito, Hidenori Tomozoe, Takashi Kawahara

**Affiliations:** 1 Faculty of Health and Sports Science, Juntendo University, Inzai-shi, Chiba, Japan; 2 Graduate School of Health and Sports Science, Juntendo University, Inzai-shi, Chiba, Japan; 3 Faculty of Psychology, Meiji Gakuin University, Minato-ku, Tokyo, Japan; 4 Faculty of Sport Culture, Nippon Sport Science University, Setagaya-ku, Tokyo, Japan; 5 Faculty of Childhood Sport Education, Nippon Sport Science University, Setagaya-ku, Tokyo, Japan; 6 Institute of Health and Sports Science & Medicine, Juntendo University, Inzai-shi, Chiba, Japan; 7 Faculty of Physical Education, International Pacific University, Okayama Higashi-ku, Okayama, Japan; 8 Japan Association for University Athletics and Sport (UNIVAS), Chiyoda-ku, Tokyo, Japan

**Keywords:** anti-doping education, clean sport, doping likelihood, doping prevention, moral disengagement, university athletes

## Abstract

**Background:**

Anti-doping education has increasingly emphasized values-based approaches that aim to foster ethical decision-making and long-term engagement in clean sport. However, relatively few studies have reported the systematic development and evaluation of educational programs specifically designed for university athletes within authentic educational contexts.

**Purpose:**

The present study aimed to develop and preliminarily evaluate a UNIVAS anti-doping education program for university sport in Japan, designed to foster doping-related decision-making competence through a values-based clean sport approach.

**Methods:**

The research was conducted in three sequential studies. Study 1: Fifteen doping-related decision-making scenarios were developed based on theoretical frameworks, previous literature, and domestic anti-doping disciplinary cases. An online survey of 1,000 university student-athletes and student-athlete support personnel examined the acceptability of each scenario and the behavioral responses participants would choose. Study 2: Selected scenarios were used to develop lecture slides, instructional scripts, and worksheets. These materials were iteratively refined through five pilot implementations conducted in university classes. Study 3: Short-term changes associated with the finalized educational program were examined using a pre-post design with two instructional formats: face-to-face and on-demand. A total of 1,241 university athletes completed measures of moral disengagement in doping, doping likelihood, and scenario acceptability before and after the intervention.

**Results:**

Study 1: The acceptability of doping-related behaviors was generally low, and responses indicating clear acceptance of wrongdoing were rare. Participants more frequently selected responses reflecting rejection, self-regulated effort, consultation, or legal alternatives. Study 2: The materials were refined into a structured educational program integrating foundational clean sport content with scenario analysis and self-explanation activities. Study 3: Moral disengagement significantly decreased after the intervention across both instructional formats. Doping likelihood also decreased, and acceptability ratings changed in several scenarios, indicating reduced tolerance for doping-related behaviors. Format-related differences were limited, although direct comparisons between formats should be interpreted cautiously because allocation was non-random and implementation timing differed.

**Conclusion:**

These findings suggest that the UNIVAS materials provide a feasible and potentially useful model for values-based anti-doping education in university sport. The program may influence psychological processes associated with doping intentions and support ethically grounded decision-making in realistic sport contexts.

## 1 Introduction

Doping continues to pose a persistent threat to fairness in sport and to the health of athletes. Athletes are exposed to various psychosocial stressors, such as competitive pressure, demands for rapid recovery from injury, and expectations from others in their environment (1). These factors may influence the formation of doping intentions and doping-related decision-making (2, 3). In addition, internal concerns related to medical, economic, and performance issues, perceptions of the legitimacy of anti-doping systems, doping-related norms, and identity-related factors have also been identified as psychosocial determinants that influence athletes’ personal commitment to clean sport (4). Reflecting the psychosocial nature of doping-related decision-making, the Incremental Model of Doping Behaviour (IMDB) has been proposed, which conceptualizes doping not as a single deviant act but as a progressively developed behavior (5). From this perspective, doping is understood as a gradual process in which behaviors are learned and morally justified over time. Accordingly, anti-doping education should not be limited to the transmission of factual knowledge but should instead be designed and evaluated to develop athletes’ ability to make appropriate decisions in complex social and moral situations (6). Within this educational context, the university years represent an important developmental stage during which identity formation and the integration of personal values progress (7). Educational interventions that support the development of decision-making abilities related to sport participation and athletic values are therefore particularly important during this period. In particular, research has shown that moral factors, including cognitive mechanisms that justify unethical behavior, play a significant role in doping-related decision-making (8–10).

Building on the importance of decision-making competence and moral factors, anti-doping education has been undergoing international reform. The World Anti-Doping Code (WADA Code) and the International Standard for Education (ISE) emphasize the development of athletes’ abilities to make judgments and act in accordance with the values of sport, highlighting the importance of values-based education (11–14). These frameworks also suggest that educational programs should be designed from the perspective of achieving intended learning outcomes and educational impact (6, 15, 16). Empirical studies conducted in multiple countries have also shown that recognition of the values of clean sport is associated with integrity and respect for rules (17). However, studies that explicitly describe the instructional design process for educational materials addressing values-based decision-making for university athletes and empirically examine the effects of such interventions on doping intentions and moral judgment remain limited.

Previous research has consistently shown that doping intentions are among the strongest psychological predictors of doping behavior (2, 3). Among the various psychosocial factors associated with doping intentions, moral disengagement has been identified as a central psychological mechanism involved in the formation of doping intentions. Based on Bandura's social cognitive theory of moral thought and action (18), moral disengagement refers to cognitive processes through which individuals justify or minimize the moral consequences of unethical behavior, such as feelings of guilt or self-condemnation (e.g., negative affect or cognitive dissonance) (10). This selective disengagement of self-sanctions operates through eight mechanisms: moral justification, euphemistic labeling, advantageous comparison, displacement of responsibility, diffusion of responsibility, distortion of consequences, dehumanization, and attribution of blame. Through these mechanisms, either independently or in combination, individuals reinterpret prohibited, unfair, or morally questionable behaviors as acceptable. In sport contexts, these mechanisms have been shown to contribute to rule violations and norm-deviant behavior (19), and they have also been applied to doping-related decision-making (8, 9). For example, athletes may justify doping as a means of improving performance (moral justification), describe the behavior in less negative terms, such as a method for maximizing potential or aiding recovery (euphemistic labeling), or minimize its seriousness by comparing it with other illegal behaviors in everyday life (advantageous comparison). They may also attribute responsibility to others or to the broader team environment (displacement of responsibility and diffusion of responsibility) or downplay the harmful consequences of the behavior (distortion of consequences). Through such mechanisms, psychological resistance to doping may be reduced, thereby facilitating the formation of doping intentions (8).

Furthermore, individual differences in moral disengagement have been shown to influence the formation of doping intentions (2). Higher levels of moral disengagement have been associated with more favorable attitudes toward doping, greater acceptance of rule-violating behavior, and stronger doping intentions (8, 9, 20–26). At the same time, intentions do not fully determine behavior. Actual doping use is also influenced by situational factors, such as substance availability, perceived detection risk, and perceived health consequences (2, 27). Therefore, to better understand doping-related decision-making, it is important to examine not only general intentions but also judgment processes in specific situational contexts.

One approach to examining such decision-making processes is to present hypothetical scenarios describing doping-related behaviors, through which athletes’ judgments and intentions can be evaluated (28–30). This approach can also serve as a useful educational tool, allowing learners to place themselves in realistic situations and consider decisions guided by values and norms. In addition, the process of explaining and justifying the reasoning behind their judgments is known as the self-explanation effect, which has been shown to promote the integration of prior knowledge with new information and to facilitate understanding and problem solving (31, 32). Such processes of explanation and justification have also been shown to support the development of argumentation skills that include claims, evidence, and justification, and to facilitate the internalization of values and norms (33, 34). From a moral judgment perspective, experiences in which individuals articulate their judgments and explain or justify them in social interactions may also serve as developmental opportunities that help translate moral reasoning into action (18, 35). This is particularly relevant for university students, who are likely to encounter situations in which they must explain or justify their decisions and responses to others. Accordingly, educational approaches that incorporate explanatory and dialogic activities may have practical significance in this context. Furthermore, evaluating the effectiveness of educational interventions requires consideration not only of changes in knowledge and attitudes but also of motivational aspects, such as learners’ perceived value of the learning task (36, 37). However, although competitive environments and broader social contexts influence doping-related decision-making (38), studies that incorporate scenario-based representations of doping-related situations into educational programs and empirically examine their effects remain limited. As a result, clear guidelines for developing instructional materials that can be implemented in educational settings have not yet been sufficiently established.

These research gaps are also evident in educational practice in university sport in Japan. In particular, within the context of Japanese university sport, anti-doping education may be delivered not only by faculty members but also by student ASP. Under the WADA Code and the ISE, anti-doping education is not limited to elite athletes, but is expected to reach athletes at different levels, athlete support personnel, and stakeholders who deliver or support education. In Japan, the Japan Anti-Doping Agency (JADA), as a Code Signatory, promotes anti-doping education and awareness in line with these international standards. However, given concerns about anti-doping rule violations in university sport, broader dissemination of education within the Japanese university sport context remains an important issue. To address this need, the Japan Sports Agency commissioned a project to promote anti-doping education in university sport, which was implemented by the Japan Association for University Athletics and Sport (UNIVAS) in collaboration with JADA (39). Accordingly, instructional materials and lesson plans are needed that can be used across diverse university settings by student-athletes, student ASP, faculty members, coaches, and others involved in supporting athletes. However, previous studies have often focused on individual educational components or short-term effects, while providing limited descriptions of the overall development process and the design principles of educational programs. In particular, the design of instructional materials that directly address doping intentions, which are among the strongest predictors of doping use (2, 3), and moral judgment (8) remains limited. Given these needs, sport organizations and cross-university initiatives need to play a central role in systematically developing and providing anti-doping educational materials that can be widely implemented in university sport settings.

Based on these research gaps, the present study aimed to develop educational materials centered on scenario-based representations of doping-related decision-making situations relevant to university student-athletes and to describe the design process of these materials. In addition, the study aimed to examine the educational effects of the intervention using the developed materials. Specifically, through both face-to-face and on-demand educational interventions, the study examined changes in doping likelihood, moral disengagement, and the acceptability of doping-related behaviors. In doing so, the study sought to provide empirical evidence regarding the relevance of values-based education and its potential as a psychology-based preventive approach to doping-related decision-making in university sport.

## 2 Methods

This study was conducted as part of the “Anti-Doping Education Promotion Project in University Sports: A Project on Anti-Doping Education for University Student-Athletes,” commissioned by the Japan Sports Agency and implemented by UNIVAS, to develop and preliminarily evaluate anti-doping educational materials for university sport settings in Japan. In collaboration with JADA, the project was carried out by an interdisciplinary research team with expertise in anti-doping, sport pedagogy, sport psychology, sport ethics, and sports medicine.

The project targeted university student-athletes and student-athlete support personnel (ASP) and was conducted in three sequential studies (Table 1). Study 1 developed fifteen scenarios representing decision-making situations that student-athletes and student ASP may encounter. Survey data were used to assess acceptability and behavioral response tendencies for each scenario and to collect foundational data to inform the design of the educational materials. Study 2 used a selected scenario to develop a lesson plan, lecture slides, and instructional script. These materials were refined through a pilot lecture and worksheet-based activities, resulting in finalized lecture slides consisting of basic and advanced components. Study 3 produced an on-demand video based on the finalized materials and examined short-term changes associated with the program using a two-group design (face-to-face and on-demand). Moral disengagement, scenario acceptability, and doping likelihood were assessed before and after the intervention, and changes were compared statistically to examine short-term changes associated with the educational intervention. All three studies (Studies 1–3) were approved by the Ethics Committee of the Faculty of Health and Sports Science and the Graduate School of Health and Sports Science, Juntendo University (IRB No. 2022-84; 2023-58; 2023-148).

**Table 1:** Study design (studies 1–3).

Study	Purpose/focus	Main content	Indicators/outcomes
1	Development of doping-related decision-making scenarios	Development of fifteen scenarios	Scenario acceptability (Likert scale)
	Collection of foundational data for scenario evaluation and educational material design	Creation of short video clips for each scenario (for consistent scenario presentation)	Behavioral responses (multiple-choice options)
2	Development and refinement of lecture-based anti-doping educational materials	Development of a lesson plan, PowerPoint lecture slides, and a structured lecture script based on one selected scenario	Learners’ perceived task value (post-intervention)
	Instructional design supporting both face-to-face and on-demand delivery	Integration of structured explanation activities using worksheets	Learner-generated worksheet responses used to refine instructional materials
		Pilot implementation through a face-to-face lecture with university students (including student-athletes and ASP)	Classroom observations through video recordings used to inform instructional refinement
		Refinement and finalization of lecture materials into three slide sets (basic and advanced components)	Feasibility and usability of the developed lecture materials
		Preparation of materials suitable for both instructor-led and on-demand delivery	
3	Evaluation of the effectiveness of the educational intervention	Pre–post assessment using a two-group design (face-to-face and on-demand)	Moral disengagement in doping
			Doping likelihood
			Scenario acceptability

### 2.1 Study 1 methods

#### 2.1.1 Phase 1: development of doping-related decision-making scenarios

The scenarios were designed as theory-informed educational stimuli intended to elicit ethical judgments in doping-related decision-making situations. To enhance ecological validity, the scenarios were constructed by incorporating situations involving heightened competitive pressure, demands for a rapid return from injury, chronic performance stagnation, and expectations from surrounding individuals (1, 40), as well as the risk of cross-contamination of supplements with prohibited substances (41–49), while also taking into account evidence suggesting that supplement use may be associated with, or may precede, prohibited substance use (50–55). The scenarios were also informed by cases reported in domestic anti-doping disciplinary panel decisions (56). Each scenario was primarily designed to reflect mechanisms of doping-related moral disengagement (8, 9, 57, 58). However, it was assumed that multiple psychological processes may simultaneously operate in real-world decision-making contexts. Therefore, the purpose of these scenarios was not to operationalize moral disengagement as a latent construct, but rather to present theoretically informed justification processes within an educational context.

**Figure 1:**
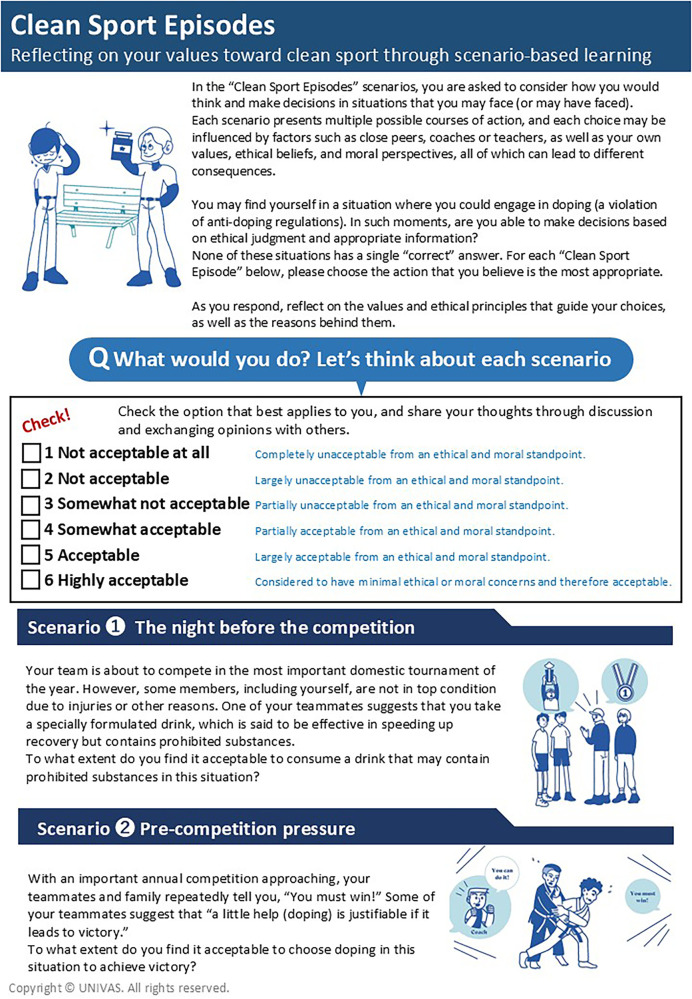
Doping-related decision-making scenarios (page 1 of 4). Translated and reproduced with permission from the Japan Association for University Athletics and Sport (UNIVAS). Copyright © UNIVAS. All rights reserved. Source: UNIVAS.

**Figure 2:**
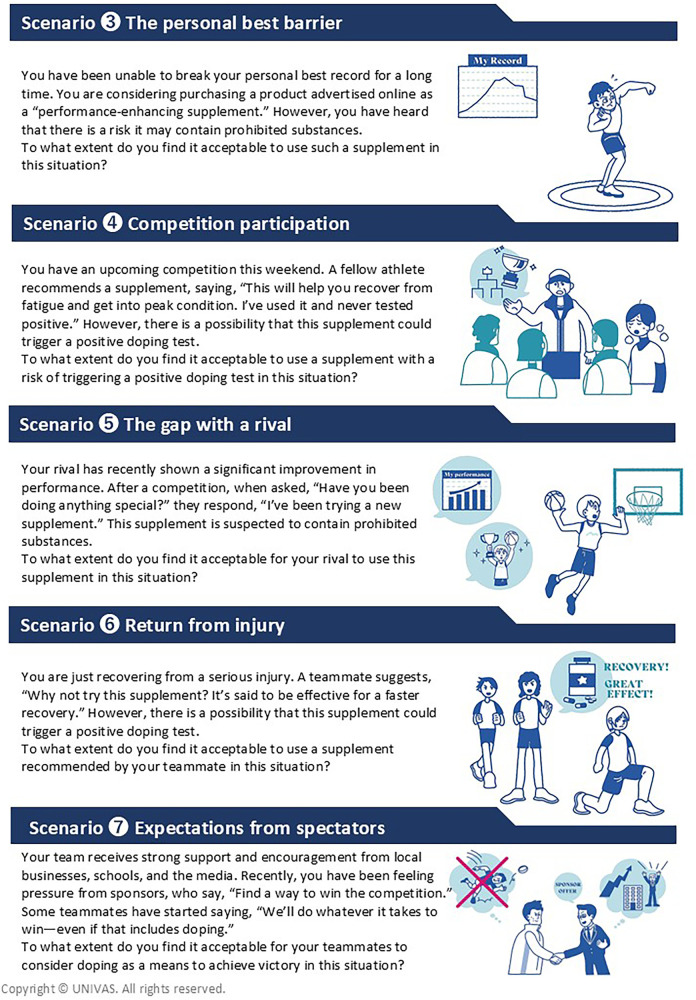
Doping-related decision-making scenarios (page 2 of 4).

**Figure 3:**
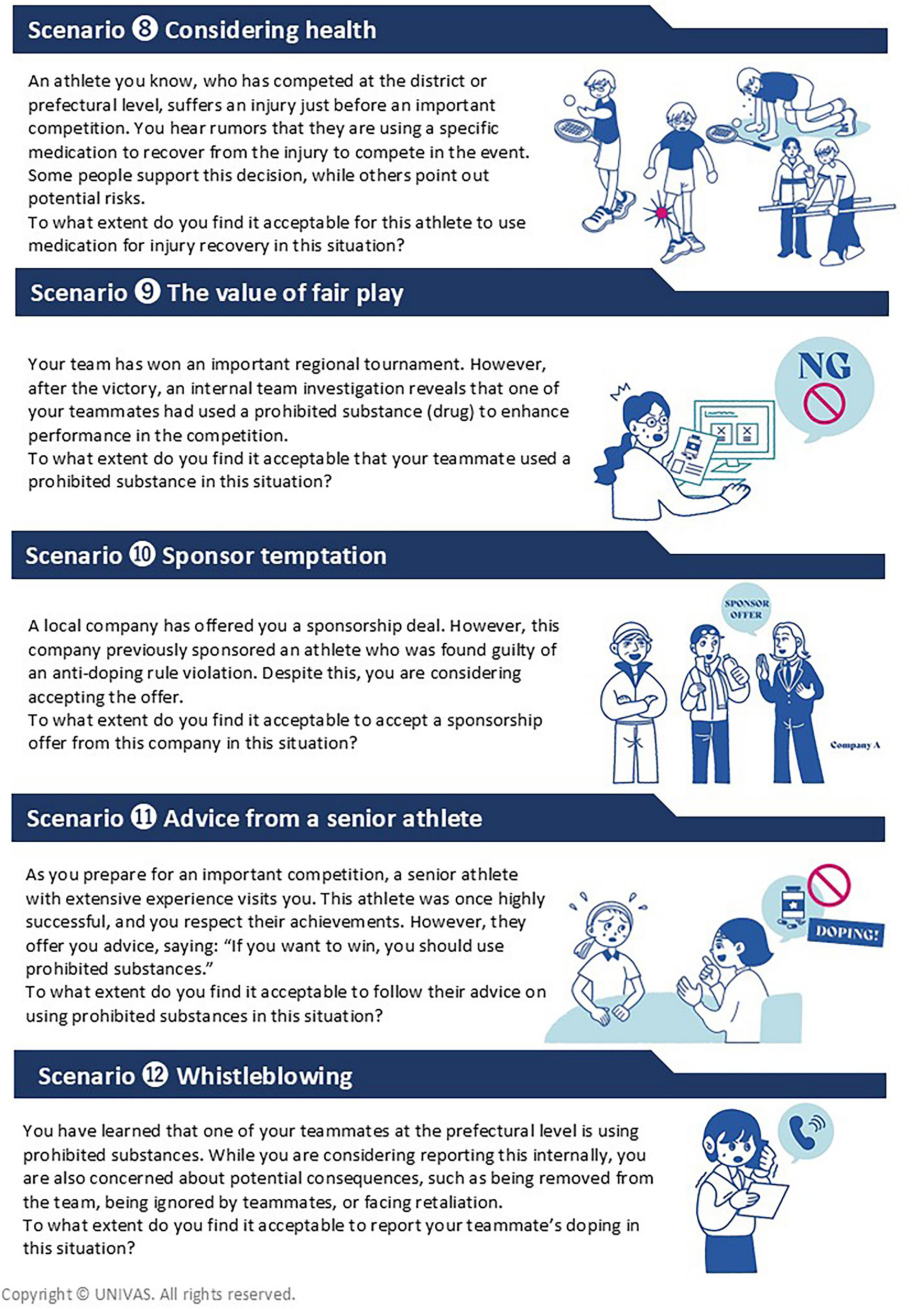
Doping-related decision-making scenarios (page 3 of 4).

**Figure 4:**
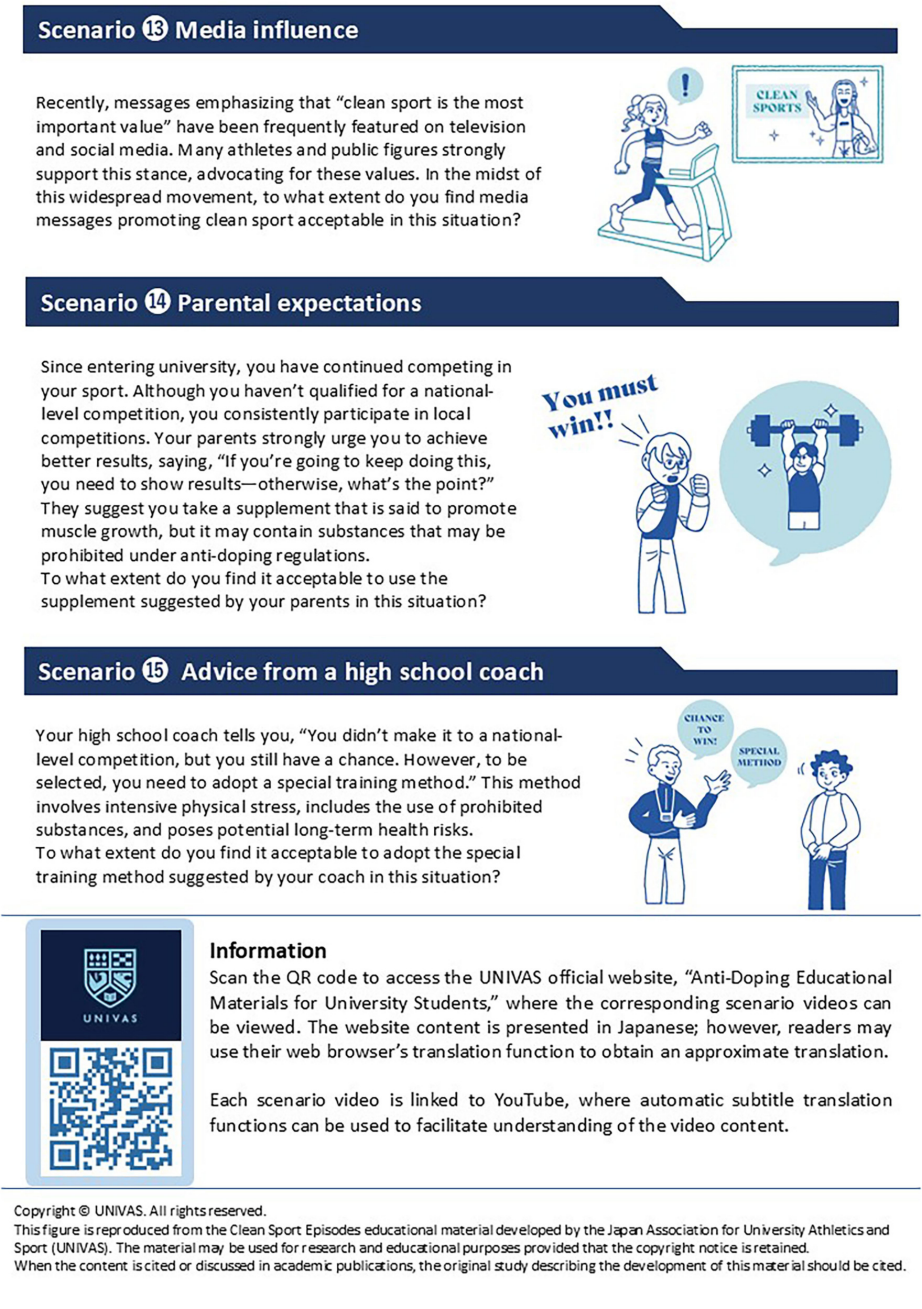
Doping-related decision-making scenarios (page 4 of 4).

Through this process, fifteen scenarios integrating these elements were finalized (Figures 1–4). The finalized scenarios encompassed diverse ethical contexts, including situations in which athletes themselves consider engaging in doping-related behavior, evaluate others’ doping, tolerate morally ambiguous recovery-related practices, face pressure from significant others or sponsors, respond to clean sport values, and engage in whistleblowing (59–61).

Conceptually, consistent with social cognitive theory of moral thought and action (18), the finalized scenarios reflected different targets of moral decision-making, distinguishing between situations in which athletes evaluate their own potential engagement in doping-related behavior, respond to others’ suspected or confirmed doping, and orient their decisions toward broader social norms and clean sport values. Accordingly, the scenarios were conceptually grouped into three categories based on the primary decision target involved: (A) self-referential decision-making (athletes’ own potential engagement in doping-related behavior: scenarios No. 1, 2, 3, 4, 6, 11, 14, and 15), (B) other-referential decision-making (responses to others’ suspected or confirmed doping: scenarios No. 5, 8, 9, and 12), and (C) normative and value-based orientation (responses to social pressures, sponsorship contexts, or clean sport values: scenarios No. 7, 10, and 13). While the scenarios were primarily informed by mechanisms of moral disengagement (8, 9, 57, 58), they were also designed to capture variation in the target of moral decision-making across different ethical contexts.

#### 2.1.2 Phase 2: survey methods

From October to November 2023, we conducted an online survey via a Japanese internet research company (My Voice Co., Ltd.). The target population consisted of university athletes and student ASP across Japan. Inclusion criteria were: (1) currently training for official competitions or serving as ASP supporting student-athletes, and (2) participating in JADA-affiliated sports. To enhance the generalizability of the findings and capture the diversity of university sport environments in Japan, participants were recruited from a wide range of sports disciplines. This approach was adopted because doping-related decision-making and moral reasoning are influenced by broader psychosocial and educational contexts rather than being confined to specific sports. ASP were also included because they represent key members of the athlete support system and are recognized as important stakeholders in anti-doping education and athlete development. The study's purpose and procedures were explained on a webpage, and informed consent was obtained from participants. Of the 1,200 individuals invited nationwide, 1,000 met the inclusion criteria and were included in the analysis. A sensitivity analysis using G*Power 3.1 (62, 63) indicated that, for a one-way ANOVA comparing three groups with a total sample size of 1,000, *α* = .05, and power = .80, the minimum detectable effect size was f = 0.098, corresponding to *η*^2^ = .010.

#### 2.1.3 Participant characteristics

The athlete sample comprised 877 participants representing 56 sports affiliated with the JADA, including 364 males (41.5%) and 513 females (58.5%), with a mean age of 20.6 ± 2.5 years. The most common sport was athletics (13.3%), followed by volleyball (9.4%), tennis (8.9%), soccer (8.6%), and basketball (8.0%). Regarding competition level, 73.8% were classified as regional (including those with no competition experience), whereas 26.2% had competed at the national or international level. Regarding anti-doping education experience, 42.9% reported having received anti-doping education.

The student ASP sample comprised 135 participants involved in 27 sports affiliated with JADA, including 25 males (20.3%) and 98 females (79.7%), with a mean age of 20.3 ± 2.1 years. The most common affiliated sports were baseball (9.8%) and athletics (9.8%), followed by volleyball (8.9%), tennis (8.1%), and swimming (8.1%). Regarding the competition level of the athletes they supported, 81.3% were regional (including those with no competition experience) and 18.7% were at the national or international level. Regarding anti-doping education experience, 36.6% reported having received anti-doping education.

#### 2.1.4 Measure

Fifteen doping-related decision-making scenarios developed in Phase 1 were designed not only as instructional materials for lecture-based and workshop settings, but also as tools that enable both participants and educators to evaluate decision-making tendencies and normative judgments within educational contexts. To facilitate their use in educational implementation and evaluation, each scenario incorporated two response formats to assess acceptability judgments and behavioral choices. For each scenario, participants rated the acceptability of the described behavior on a 6-point Likert scale ranging from 1 (not acceptable at all) to 6 (highly acceptable). Higher mean scores indicated greater acceptability of doping-related behavior.

Acceptability ratings reflected the strength of normative attitudes toward the behavior and were treated as continuous variables. In addition, for each scenario, participants selected one behavioral response option that best reflected the action they would take (e.g., accept the proposal, reject it, suggest an alternative, consult a relevant person, or other). An open-ended option was also included to reduce forced-choice bias. Behavioral response options were treated as categorical variables. For student ASP, participants were instructed to respond from the perspective of support staff rather than as athletes. Accordingly, student ASP rated the acceptability of the athlete's doping-related behavioral response option that best reflected how they would act in their role as support staff in each situation.

#### 2.1.5 Statistical analysis

All statistical analyses in Studies 1 through 3 were conducted using IBM SPSS Statistics 31 (IBM Corp., Tokyo, Japan), with a significance level of *α* = .05. First, descriptive statistics (mean, standard deviation, and median) were calculated for acceptability ratings for each scenario. As an exploratory analysis, participants were categorized into three groups: (1) athletes whose highest competition level was regional (i.e., district or regional competitions), (2) athletes who had competed at the national or international level, and (3) student ASP. Group differences in acceptability ratings for each scenario were examined using one-way analysis of variance (ANOVA), and eta squared (*η*^2^) was reported as the effect size. The interpretation of *η*^2^ values was as follows: 0.01, small; 0.06, medium; and 0.14, large (64). *post hoc* comparisons were conducted with the Bonferroni correction. Behavioral response options for each scenario were categorical; therefore, group differences in response distributions were examined using cross-tabulations and *χ*^2^ tests. Cramér's V statistic was reported as the effect size. The interpretation of V coefficients was as follows: 0.05, weak; 0.10, moderate; 0.15, strong; and 0.25, very strong (65).

### 2.2 Results

#### 2.2.1 Acceptability ratings across scenario types

The one-way ANOVA showed a significant group difference only for Scenario 1 (*p* = .015) (Table 2). *post hoc* comparisons indicated that student ASP reported higher acceptability than athletes who had competed at the national or international level. For all other scenarios, group differences were not statistically significant, and effect sizes were small, indicating minimal differences between groups.

**Table 2:** Acceptability ratings by athletic level and student ASP.

Scenario	Athletic level				Student ASP (*N* = 135)		*p*	*η*^2^ [95% CI]	MCT
	Regional (*N* = 635)		National and international (*N* = 230)						
	*M*	*SD*	*M*	*SD*	*M*	*SD*			
1. The night before the competition	2.16	1.19	1.97	1.12	2.33	1.29	.015	.008 [.000,.022]	ASP > National or International
2. Pre-competition pressure	1.97	1.11	1.92	1.23	2.15	1.16	.166	.004 [.000,.013]	*n.s.*
3. The personal best barrier	2.06	1.11	1.96	1.16	2.21	1.12	.114	.004 [.000,.015]	*n.s.*
4. Competition participation	2.11	1.14	1.98	1.16	2.13	0.96	.265	.003 [.000,.011]	*n.s.*
5. The gap with a rival	1.96	1.07	1.84	1.04	2.03	1.02	.216	.003 [.000,.012]	*n.s.*
6. Return from injury	2.03	1.13	2.02	1.19	2.14	1.18	.550	.001.001 [.000,.008]	*n.s.*
7. Expectations from spectators	1.81	1.07	1.78	1.12	1.96	1.15	.297	.002 [.000,.011]	*n.s.*
8. Considering health	2.27	1.09	2.13	1.15	2.37	1.16	.094	.005 [.000,.015]	*n.s.*
9. The value of fair play	1.90	1.07	1.84	1.06	2.04	1.23	.244	.003.003 [.000,.012]	*n.s.*
10. Sponsor temptation	2.75	1.44	2.63	1.39	2.82	1.39	.394	.002 [.000,.009]	*n.s.*
11. Advice from a senior athlete	1.84	1.10	1.71	1.05	1.95	1.16	.124	.004.004 [.000,.014]	*n.s.*
12. Whistleblowing*	3.05	1.68	2.94	1.67	2.93	1.57	.580	.001.001 [.000,.007]	*n.s.*
13. Media influence*	3.54	1.75	3.56	1.77	3.49	1.70	.928	.000 [.000,.002]	*n.s.*
14. Parental expectations	1.95	1.09	1.88	1.09	2.10	1.22	.172	.004 [.000,.013]	*n.s.*
15. Advice from a high school coach	1.79	1.07	1.67	1.08	1.96	1.17	.056	.006 [.000,.017]	*n.s.*

Response options ranged from “1 = Not acceptable at all” to “6 = Highly acceptable.”

(A) self-referential decision-making (No. 1, 2, 3, 4, 6, 11, 14, and 15), (B) other-referential decision-making (No. 5, 8, 9, and 12), and (C) normative and value-based orientation (No. 7, 10, and 13).

M, Mean; SD, standard deviation; MCT, multiple comparison testing; n.s., not significant; ASP, Student ASP.

* Higher scores indicate greater acceptance of doping-related behavior for Scenarios 1–11 and 14–15, whereas higher scores indicate stronger endorsement of clean sport–related responses for Scenarios 12 and 13.

When interpreted according to the theoretically defined categories, acceptability ratings in the (A) self-referential decision-making scenarios (No. 1, 2, 3, 4, 6, 11, 14, and 15) were generally low across all groups (means approximately 1.7–2.3). In the (B) other-referential decision-making scenarios (No. 5, 8, 9, and 12), acceptability ratings for tolerating others’ doping-related behavior were likewise low in Scenarios 5, 8 and 9. Scenario 12 was described separately because higher scores indicated greater acceptability of reporting a teammate's doping, unlike the other doping-tolerance senarios; its mean scores were relatively higher across groups (approximately 2.9–3.1). In the (C) normative and value-based scenarios (No. 7, 10, and 13), moderate levels of acceptability were observed overall. Scenario 13 was also described separately because higher scores indicated greater acceptability of clean sport-oriented responses to media messages; it showed the highest mean scores among all scenarios (approximately 3.5–3.6).

#### 2.2.2 Behavioral response distributions across scenario types

Behavioral response distributions for each scenario and group are shown in Table 3. Across scenarios involving doping-related wrongdoing, responses indicating acceptance or tolerance of such wrongdoing were rare. Chi-square analyses showed statistically significant group differences in response distributions for several scenarios, although effect sizes were small (Cramér's V = .080–.125). In the (A) self-referential scenarios (No. 1, 2, 3, 4, 6, 11, 14, and 15), participants most frequently selected self-regulated options (e.g., refusing the proposal, suggesting legal alternatives, or relying on their own effort) and consultation-based options (e.g., consulting coaches, medical professionals, or support staff). In the (B) other-referential scenarios (No. 5, 8, 9, and 12), consultation-based responses and direct engagement options were selected more frequently than responses indicating tolerance or inaction in Scenarios 5, 8, and 9. For Scenario 12, which involved reporting a teammate's doping, reporting- or consultation-oriented responses were selected more frequently than tolerance or inaction. In the (C) value-based scenarios (No. 7, 10, and 13), responses reflecting alignment with clean sport values and consultation with others were most frequently selected, including clean sport-oriented responses in Scenario 13.

**Table 3:** Behavioral responses by athletic level and student ASP.

Scenario	Response option	Athletic level		Student ASP (*N* = 135)	*χ*^2^ (12)	*p*	V
		Regional (*N* = 635)	National or international (*N* = 230)				
1. The night before the competition (A)	Accept proposal	9 (5.9%)	35 (5.1%)	3 (1.9%)	19.754	.072	.099
	Reject & admonish	36 (23.5%)	182 (26.3%)	40 (25.8%)			
	Suggest legal alternative	42 (27.5%)	241 (34.9%)	48 (31.0%)			
	Consult coach	33 (21.6%)	114 (16.5%)	33 (21.3%)			
	Consult staff	17 (11.1%)	38 (5.5%)	17 (11.0%)			
	Consult peers	10 (6.5%)	43 (6.2%)	6 (3.9%)			
	Consult family	6 (3.9%)	38 (5.5%)	8 (5.2%)			
2. Pre-competition pressure (A)	Agree to dope	4 (2.6%)	17 (2.5%)	5 (3.2%)	12.703	.391	.080
	Oppose proposal	43 (28.1%)	244 (35.3%)	41 (26.5%)			
	Suggest legal alternative	44 (28.8%)	211 (30.5%)	51 (32.9%)			
	Consult coach	24 (15.7%)	93 (13.5%)	26 (16.8%)			
	Consult staff	15 (9.8%)	46 (6.7%)	14 (9.0%)			
	Consult peers	14 (9.2%)	35 (5.1%)	7 (4.5%)			
	Consult family	9 (5.9%)	45 (6.5%)	11 (7.1%)			
3. The personal best barrier (A)	Purchase and use supplement	6 (3.9%)	21 (3.0%)	3 (1.9%)	30.865	.002	.124
	Do not purchase; rely on own effort	36 (23.5%)	243 (35.2%)	40 (25.8%)			
	Explore legal performance methods	44 (28.8%)	211 (30.5%)	54 (34.8%)			
	Consult coach	27 (17.6%)	99 (14.3%)	32 (20.6%)			
	Consult support staff	25 (16.3%)	45 (6.5%)	11 (7.1%)			
	Consult peers	10 (6.5%)	33 (4.8%)	6 (3.9%)			
	Consult family	5 (3.3%)	39 (5.6%)	9 (5.8%)			
4. Competition participation (A)	Decline friend's recommendation	41 (26.8%)	300 (43.4%)	56 (36.1%)	27.635	.006	.118
	Accept and use	7 (4.6%)	16 (2.3%)	4 (2.6%)			
	Consult medical professional	27 (17.6%)	107 (15.5%)	30 (19.4%)			
	Consult coach	34 (22.2%)	138 (20.0%)	32 (20.6%)			
	Consult support staff	20 (13.1%)	50 (7.2%)	7 (4.5%)			
	Consult peers	17 (11.1%)	45 (6.5%)	18 (11.6%)			
	Consult family	7 (4.6%)	35 (5.1%)	8 (5.2%)			
5. The gap with a rival (B)	Do not suspect rival; rely on own effort	30 (19.6%)	207 (30.0%)	32 (20.6%)	22.024	.037	.105
	Express concern directly to rival	27 (17.6%)	143 (20.7%)	27 (17.4%)			
	Consult coach	31 (20.3%)	113 (16.4%)	39 (25.2%)			
	Consult support staff	21 (13.7%)	66 (9.6%)	12 (7.7%)			
	Consult peers	18 (11.8%)	56 (8.1%)	14 (9.0%)			
	Report to JADA	19 (12.4%)	76 (11.0%)	20 (12.9%)			
	Consult family	7 (4.6%)	30 (4.3%)	11 (7.1%)			
6. Return from injury (A)	Use recommended supplement	8 (5.2%)	18 (2.6%)	2 (1.3%)	30.464	.002	.123
	Recover naturally	40 (26.1%)	278 (40.2%)	43 (27.7%)			
	Consult medical professional	40 (26.1%)	162 (23.4%)	48 (31.0%)			
	Consult coach	30 (19.6%)	93 (13.5%)	27 (17.4%)			
	Consult support staff	19 (12.4%)	59 (8.5%)	12 (7.7%)			
	Consult peers	13 (8.5%)	41 (5.9%)	14 (9.0%)			
	Consult family	3 (2.0%)	40 (5.8%)	9 (5.8%)			
7. Expectations from spectators (C)	Use doping if necessary to win	4 (2.6%)	10 (1.4%)	0 (0.0%)	30.768	.002	.124
	Discuss with team and aim to perform best	52 (34.0%)	354 (51.2%)	70 (45.2%)			
	Consult coach	35 (22.9%)	131 (19.0%)	33 (21.3%)			
	Consult support staff	17 (11.1%)	37 (5.4%)	21 (13.5%)			
	Consult peers	17 (11.1%)	60 (8.7%)	11 (7.1%)			
	Inform sponsor	16 (10.5%)	53 (7.7%)	11 (7.1%)			
	Consult family	12 (7.8%)	46 (6.7%)	9 (5.8%)			
8. Considering health (B)	Point out the issue directly to athlete	20 (13.1%)	117 (16.9%)	25 (16.1%)	21.673	.041	.104
	Pretend not to notice	19 (12.4%)	114 (16.5%)	12 (7.7%)			
	Consult coach	33 (21.6%)	168 (24.3%)	41 (26.5%)			
	Consult support staff	31 (20.3%)	86 (12.4%)	30 (19.4%)			
	Consult peers	19 (12.4%)	86 (12.4%)	20 (12.9%)			
	Report to anti-doping organization	24 (15.7%)	71 (10.3%)	18 (11.6%)			
	Consult family	7 (4.6%)	49 (7.1%)	9 (5.8%)			
9. The value of fair play (B)	Observe situation without action	18 (11.8%)	117 (16.9%)	11 (7.1%)	27.288	.007	.117
	Consult coach	40 (26.1%)	230 (33.3%)	59 (38.1%)			
	Consult support staff	21 (13.7%)	60 (8.7%)	18 (11.6%)			
	Consult peers	18 (11.8%)	64 (9.3%)	13 (8.4%)			
	Propose team meeting	40 (26.1%)	115 (16.6%)	26 (16.8%)			
	Speak privately to athlete	9 (5.9%)	60 (8.7%)	17 (11.0%)			
	Consult family	7 (4.6%)	45 (6.5%)	11 (7.1%)			
10. Sponsor temptation (C)	Decline offer	33 (21.6%)	155 (22.4%)	28 (18.1%)	15.317	.225	.088
	Accept offer	20 (13.1%)	75 (10.9%)	17 (11.0%)			
	Consult coach	38 (24.8%)	183 (26.5%)	35 (22.6%)			
	Consult support staff	20 (13.1%)	64 (9.3%)	25 (16.1%)			
	Consult peers	18 (11.8%)	52 (7.5%)	14 (9.0%)			
	Investigate company background	19 (12.4%)	123 (17.8%)	26 (16.8%)			
	Consult family	5 (3.3%)	39 (5.6%)	10 (6.5%)			
11. Advice from a senior athlete (A)	Ignore advice	44 (28.8%)	296 (42.8%)	52 (33.5%)	23.306	.025	.108
	Accept advice	11 (7.2%)	29 (4.2%)	6 (3.9%)			
	Argue against advice	29 (19.0%)	113 (16.4%)	32 (20.6%)			
	Consult coach	31 (20.3%)	120 (17.4%)	27 (17.4%)			
	Consult support staff	20 (13.1%)	44 (6.4%)	19 (12.3%)			
	Consult peers	8 (5.2%)	42 (6.1%)	10 (6.5%)			
	Consult family	10 (6.5%)	47 (6.8%)	9 (5.8%)			
12. Whistleblowing (B) *	Do not consult anyone	12 (7.8%)	58 (8.4%)	9 (5.8%)	19.630	.074	.099
	Consult JADA	36 (23.5%)	159 (23.0%)	37 (23.9%)			
	Consult sport federation	14 (9.2%)	69 (10.0%)	14 (9.0%)			
	Consult coach	45 (29.4%)	205 (29.7%)	49 (31.6%)			
	Consult support staff	23 (15.0%)	45 (6.5%)	10 (6.5%)			
	Consult peers	16 (10.5%)	77 (11.1%)	17 (11.0%)			
	Consult family	7 (4.6%)	78 (11.3%)	19 (12.3%)			
13. Media influence (C) *	Maintain own values	34 (22.2%)	218 (31.5%)	27 (17.4%)	31.213	.002	.125
	Change values due to media	20 (13.1%)	91 (13.2%)	22 (14.2%)			
	Seek advice from coach	25 (16.3%)	95 (13.7%)	19 (12.3%)			
	Seek advice from support staff	21 (13.7%)	48 (6.9%)	20 (12.9%)			
	Discuss values with peers	31 (20.3%)	99 (14.3%)	29 (18.7%)			
	Discuss values within team	13 (8.5%)	86 (12.4%)	27 (17.4%)			
	Seek advice from family	9 (5.9%)	54 (7.8%)	11 (7.1%)			
14. Parental expectations (A)	Decline proposal	55 (35.9%)	357 (51.7%)	65 (41.9%)	23.233	.026	.108
	Accept proposal	5 (3.3%)	25 (3.6%)	3 (1.9%)			
	Consult medical professional	25 (16.3%)	72 (10.4%)	23 (14.8%)			
	Consult coach	27 (17.6%)	90 (13.0%)	27 (17.4%)			
	Consult support staff	20 (13.1%)	52 (7.5%)	17 (11.0%)			
	Consult peers	14 (9.2%)	58 (8.4%)	10 (6.5%)			
	Seek psychological support	7 (4.6%)	37 (5.4%)	10 (6.5%)			
15. Advice from a high school coach (A)	Reject advice	54 (35.3%)	323 (46.7%)	58 (37.4%)	21.401	.045	.103
	Accept advice	10 (6.5%)	18 (2.6%)	5 (3.2%)			
	Consult current coach	34 (22.2%)	133 (19.2%)	32 (20.6%)			
	Consult support staff	16 (10.5%)	54 (7.8%)	17 (11.0%)			
	Consult peers	14 (9.2%)	53 (7.7%)	15 (9.7%)			
	Reconsider after checking health risks	17 (11.1%)	52 (7.5%)	19 (12.3%)			
	Consult family	8 (5.2%)	58 (8.4%)	9 (5.8%)			

(A) self-referential decision-making (No. 1, 2, 3, 4, 6, 11, 14, and 15), (B) other-referential decision-making (No. 5, 8, 9, and 12), and (C) normative and value-based orientation (No. 7, 10, and 13).

*Higher scores indicate greater acceptance of doping-related behavior for Scenarios 1–11 and 14–15, whereas higher scores indicate stronger endorsement of clean sport–related responses for Scenarios 12 and 13.

## 3 Study 2

### 3.1 Purpose

Study 2 aimed to develop an anti-doping educational program for university students by iteratively refining the instructional materials and lesson structure through repeated pilot implementations in authentic university educational settings. The program integrated a Foundational Module on the basic principles of clean sport with two Applied Modules based on selected scenarios from the fifteen doping-related decision-making scenarios developed in Study 1. These scenarios represented two distinct referential contexts of doping-related decision-making: self-referential decision-making, in which athletes consider a decision concerning themselves, and other-referential decision-making, in which athletes consider a decision involving another athlete. Through this process, Study 2 sought to establish a pedagogically structured instructional program suitable for university-based clean sport education in Japan.

### 3.2 Design framework

The educational materials used in Study 2 were developed through pilot implementation and iterative refinement in authentic university classroom settings. The development process was informed by principles of iterative instructional design commonly used in educational research and was conceptually informed by approaches described in design-based research (66–69). However, Study 2 was not intended as a full design-based research project; rather, it aimed to develop and refine instructional materials for anti-doping education in university contexts. The design of the educational program was grounded in a values-based approach to clean sport education, which emphasizes the development of athletes’ decision-making competence and anti-doping literacy (6, 12, 14). To support learners’ understanding of doping-related decisions in realistic sport situations, the program incorporated scenario-based learning activities in which participants analyze decision-making situations situated in sport contexts.

In addition, the instructional design emphasized self-explanation activities in which learners articulated and justified their own judgments. Explaining and justifying one's reasoning has been shown to promote deeper understanding through the self-explanation process and may support learners in connecting their judgments with underlying values (32, 34). Because doping-related decisions may be influenced by social pressures and processes of moral justification, perspectives from social cognitive theory and moral disengagement research were also incorporated into the instructional design (8–10, 18). Finally, the program design incorporated perspectives from task value theory by helping learners connect doping-related issues to their own sport contexts and recognize the relevance of the learning task (36).

### 3.3 Educational materials

The program was designed based on a competency-oriented, values-based approach to anti-doping education intended to support athletes’ situated decision-making competence and anti-doping literacy (6, 12, 14). The instructional materials consisted of lecture slides, slide notes (delivery scripts), and worksheets. The lecture slides were used to present the fundamental concepts of clean sport and anti-doping, while the slide notes served as instructional scripts to promote consistency in the explanations provided during the lessons.

These materials were designed to present concrete doping-related situations to support learners’ understanding of clean sport values and doping-related decision-making processes. Learners engaged in explanation activities in which they analyzed these situations and articulated the reasons for their judgments. The program integrated lecture content addressing the foundational concepts of clean sport with self-explanation activities (32, 34), using two doping-related decision-making scenarios developed in Study 1: Scenario No. 3 (“The personal best barrier”), representing a self-referential decision-making context, and Scenario No. 5 (“The gap with a rival”), representing an other-referential decision-making context. These two scenarios were selected to include one self-referential and one other-referential decision-making context in the instructional activities.

Worksheets were designed to support learners’ understanding and decision-making processes in a step-by-step manner. The worksheet activities were structured around three stages: (1) organizing key concepts related to clean sport and anti-doping; (2) analyzing the social influences surrounding the athlete; and (3) developing response strategies for addressing and persuading individuals who may pressure or encourage an athlete to dope. These worksheet activities guided learners through a sequence of conceptual understanding, situational analysis, and articulation of personal responses. This sequence was intended to help learners connect clean sport principles with concrete decision-making situations.

### 3.4 Instructional design and lesson flow

The instructional session was structured around four phases: foundational instruction, scenario analysis, explanation activity, and reflection. The foundational instruction introduced the definition and values of clean sport, anti-doping rules and rule violations, and the importance of fairness and integrity in sport. During this phase, learners completed corresponding worksheet items. During the scenario analysis phase, learners identified individuals surrounding the athlete (e.g., coaches, teammates, and family members) and considered the possible positions these actors might take in doping-related decision-making situations. Scenario No. 3, “The personal best barrier,” and Scenario No. 5, “The gap with a rival,” were then presented as the focal cases for analysis. In the explanation activity, learners described how they would respond to someone who might recommend or tolerate doping and how they would attempt to persuade that person. Finally, a whole-class reflection was conducted to revisit clean sport values and behavioral responses aligned with those values.

Across the pilot implementations, the scenario analysis and explanation activities were delivered either through small-group discussion or an individually structured worksheet, depending on the lesson version. In the individually structured format, learners followed the scenario presented in the slides and recorded their responses on the worksheet step by step. These instructional components were designed to guide learners sequentially from conceptual understanding to situational analysis and the articulation of personal decisions. Through explanation and perspective-taking activities, the program was intended to encourage active cognitive processing and reasoning (70, 71).

### 3.5 Participants

Pilots 1 and 2 were conducted as an initial trial of the educational materials in undergraduate classes within a university department specializing in sport sciences. Because Pilots 1 and 2 were exploratory classroom trials, participant characteristics were collected only to a limited extent, including sex and current athletic involvement. Pilot 1 included 37 students, comprising 24 males and 13 females, and Pilot 2 included 44 students, comprising 32 males and 12 females. Reflecting the characteristics of the student population in this department, approximately 70% of the participants were athletes.

Pilot 3 was conducted in an undergraduate psychology course. A total of 135 students attended the class, of whom 65 agreed to participate in the survey. The Pilot 3 sample therefore comprised 65 undergraduate students: 15 males (23.1%) and 50 females (76.9%), with a mean age of 19.6 ± 0.7 years. Athletes accounted for 13.8% of the participants, and the sports represented mainly included basketball (3.1%), badminton (3.1%), soccer (1.5%), and track and field (1.5%). In addition, 3.1% of participants were involved in sport as student ASP. The proportion of participants reporting previous sport experience was 61.5% in junior high school and 55.4% in high school.

Pilots 4 and 5 were conducted in undergraduate classes within a university department specializing in sport sciences. In Pilot 4, 34 students participated in the survey, including 23 males (67.6%) and 11 females (32.4%), with a mean age of 19.6 ± 0.6 years. Athletes accounted for 73.5% of the participants, and the sports represented mainly included basketball (14.7%), baseball (14.7%), and lacrosse (11.8%). In addition, 2.9% of participants were involved in sport as student support staff. In Pilot 5, 41 students participated in the survey, including 28 males (68.3%) and 13 females (31.7%), with a mean age of 19.7 ± 0.7 years. Athletes accounted for 63.4% of the participants, and the sports represented mainly included lacrosse (9.8%), ultimate (4.9%), dance (4.9%), and baseball (4.9%). In addition, 2.4% of participants were involved in sport as student support staff. The proportions of participants reporting previous sport experience in junior high school and in high school were 97.1% and 97.1%, respectively, in Pilot 4, and 97.6% and 100.0%, respectively, in Pilot 5.

### 3.6 Methods

#### 3.6.1 Measurement

Task value was assessed using the Task Value Rating Scale developed by Inada (72), which is based on the task value framework proposed by Eccles and Wigfield (36). The scale measures learners' perceived value of learning content and consists of five dimensions: institutional utility value, practical utility value, interest value, public attainment value, and private attainment value. The original instrument contained 30 items, with six items for each of the five dimensions, and all items were used in Study 2. Participants were asked to evaluate the value of the learning content addressed in the educational session, namely, “clean sport” and anti-doping, including the importance of protecting fairness in sport. Specifically, they were instructed to indicate the extent to which each statement applied to the content covered in the lesson. Responses were recorded on a 5-point Likert scale ranging from 1 (strongly disagree) to 5 (strongly agree). Subscale scores were calculated as the mean of the items corresponding to each subscale. The list of items and their dimensional classification is provided in Supplementary Material 2.

#### 3.6.2 Procedure

The pilot instructional sessions were conducted between November 2023 and November 2024 in classroom settings at two universities: one in a department of sport sciences and the other in a department of psychology. The sessions were delivered as part of seminar-style undergraduate courses. Four instructors specializing in sport pedagogy and psychology, all of whom were members of the project team, conducted the instructional sessions according to a standardized implementation protocol. Each session lasted approximately 90 min. The instructional sessions used lecture slides and structured worksheets corresponding to the lesson version used in each pilot implementation. After each instructional session, participants completed a questionnaire assessing task value related to the learning content and activities. During the pilot sessions, classroom observations were conducted to examine how learners engaged with the instructional activities and to identify potential areas for improvement in the instructional materials and lesson structure. After each session, the instructional team reviewed the classroom observation and video recordings and documented issues related to the instructional design.

#### 3.6.3 Statistical analysis

Task value ratings were analyzed descriptively to examine learners’ perceived value of the learning content and activities implemented during the pilot sessions. Means, standard deviations, and Cronbach's *α* coefficients were calculated for each task value subscale. Internal consistency was interpreted according to the following criteria: *α* ≥ 0.9, excellent; 0.8 ≤ *α* < 0.9, good; 0.7 ≤ *α* < 0.8, acceptable; 0.6 ≤ *α* < 0.7, questionable; and *α* < 0.6, poor (73). The descriptive findings were used to inform the iterative refinement of the instructional materials and lesson structure.

### 3.7 Results

#### 3.7.1 Overview

Five pilot instructional sessions were conducted between 2023 and 2024 in university classroom settings as part of the development of an anti-doping educational program for university students. Across these pilot implementations, the instructional materials were organized into three stages: an initial version used in Pilots 1–2, a revised version used in Pilots 3–5, and a final version developed after completion of all five pilots. Table 4 summarizes the progression of instructional material development across these stages, while the detailed structures of the initial, revised, and final versions are presented in Tables 5a–c, respectively.

**Table 4:** Progression of the instructional materials across pilot implementations.

Material version	Corresponding pilots	Main structural characteristics	Major revisions introduced	Role in program development
Initial version	Pilots 1–2	Lecture-based foundational content combined with a worksheet-supported scenario activity using Scenario No. 3 (“The personal best barrier”). The applied part included recognition of the athlete's social environment, interpretation of survey results, and group discussion on response strategies.	—	Used to examine the feasibility of the initial instructional design and to identify practical issues related to worksheet clarity, lesson pacing, and the group discussion format.
Revised version	Pilots 3–5	Reorganized lesson structure linking foundational content and scenario analysis more explicitly. The Foundation Module was expanded, and the explanation activity for Scenario No. 3 was shifted from a group discussion format to an individual worksheet-based format.	Added or expanded content on sport values, the philosophy of clean sport education, athlete categories based on competition level, anti-doping education topics, learning objectives, and additional explanations of doping and anti-doping rule violations. The applied activity was restructured into an individually completed format with self-reflection tasks.	Used to implement the revised instructional design across different class contexts and to examine its feasibility, manageability, and consistency.
Final version	Developed after all five pilots; used in Study 3	Final program consisting of a Foundation Module, Applied Module 1, and Applied Module 2. The instructional flow was further clarified, and both self-referential and other-referential decision-making contexts were included.	Added transitional components within the Foundation Module, elaborated Applied Module 1 with content on acceptability, behavioral choices, and psychological mechanisms of doping, and newly incorporated Applied Module 2 using Scenario No. 5 (“The gap with a rival”).	Finalized as the instructional program for evaluation in Study 3.

**Table 5a:** Structure of the initial lesson (Pilots 1–2).

No.	Module	Phase	Content	Learning activities	Objective
1	Explanation	Introduction	Clean sport education title	Lecture introduction	Presentation of lesson theme
2	Explanation	Pre-lesson discussion rules	How to conduct discussions (look at the speaker's face; praise after speaking etc.)	Group preparation	Establishing a discussion environment
3	Explanation	Presentation of lesson objectives	Be able to explain the value and importance of clean sport to others	Lecture	Sharing learning objectives
4	Explanation	Explanation of learning Content	Confirmation of objectives and learning content, and procedure	Lecture explanation	Understanding lesson structure
5	Explanation	Reconfirmation of objectives	Reconfirming the value and importance of clean sport	Lecture	Reinforcement of learning objectives
6	Foundation module	Learning Content	Understanding the need to protect fairness in sport	Lecture	Direction of thinking
7	Foundation module	Importance of fairness	Fairness as a fundamental condition for sport	Lecture	Understanding sport values
8	Foundation module	Definition of doping	Doping and the 11 anti-doping rule violations (WADA Code)	Lecture	Understanding anti-doping
9	Foundation module	Clean sport concept	Anti-doping, sports integrity and the concept of clean sport (JADA framework)	Question-based interaction	Understanding the concept of clean sport
10	Foundation module	Values and integrity of sports	Sport values and sport integrity	Question-based interaction	Understanding value concepts
11	Foundation module	Responsibilities of sport stakeholders	Actions expected of those involved in sport	Lecture	Understanding responsibility for action
12	Applied module	Learning Content	Thinking about what we can do to uphold fairness in sport	Lecture	Introduction to action-oriented thinking
13	Applied module	Recognition of the social environment	Identifying people surrounding the athlete	Group discussion	Understanding social influence
14	Applied module	Presentation of decision-making materials	Scenario 3 “The personal best barrier"”	Scenario analysis	Examining doping-related decision making
15	Applied module	Learning Content	Thinking about what you can do to uphold fairness in sport	Lecture	Introduction to action-oriented thinking
16	Applied module	Presentation of survey results	Survey data related to scenario 3 “The personal best barrier” responses (UNIVAS-Japan)	Data interpretation	Comparing personal and group perspectives
17	Applied module	Consideration of response strategies	Considering how to respond to people encouraging doping	Group discussion	Exploring action strategies
18	Applied module	Summary	Review of learning content	Reflection	Learning integration

**Table 5b:** Structure of the revised lesson (Pilots 3–4).

No.	Module	Phase	Content	Learning activities	Objective
1	Explanation	Introduction	Clean sport education title	Lecture introduction	Presentation of lesson theme
2	Explanation	Presentation of learning contents*	① What is clean sport ② The importance of clean sport ③ Awareness of clean sport	Lecture	Overview of lesson structure
3	Foundation module	Values of sport*	List the values of sport based on experience in playing, watching, learning, and supporting sport	Question-based interaction	Understanding the values of sport
4	Foundation module	Examples of sport values*	Respect, effort, cooperation, fun, courage, challenge	Lecture	Recognizing concrete examples of sport values
5	Foundation module	Clean sport concept	Anti-doping, sports integrity and the concept of clean sport (JADA framework)	Question-based interaction	Understanding the concept of clean sport
6	Foundation module	Philosophy of clean sport education*	Role of clean sport education in promoting sport values and ethical behavior	Question-based interaction	Understanding the philosophy of clean sport education
7	Foundation module	Competition level and athlete categories*	Athlete categories based on competition level (WADA-ISE framework)	Lecture explanation	Understanding differences in athlete environments
8	Foundation module	Anti-doping education topics*	11 topics in anti-doping education (WADA-ISE framework)	Lecture	Understanding the scope of anti-doping education
9	Foundation module	Presentation of learning objectives*	‘Be aware of,’ ‘understand,’ ‘Be capable of doing’ (WADA-ISE framework)	Lecture	Sharing learning objectives
10	Foundation module	Learning content*	The importance of clean sport	Lecture	Reinforcement of learning objectives
11	Foundation module	Definition of doping*	Meaning of doping and prohibited methods	Question-based interaction	Understanding what constitutes doping
12	Foundation module	Definition of doping	Doping and the 11 anti-doping rule violations (WADA Code)	Lecture explanation	Understanding anti-doping rules
13	Foundation module	Definition of doping*	Additional explanations of what is doping (WADA framework)	Question-based interaction	Deepening understanding of anti-doping rules
14	Foundation module	Anti-doping philosophy*	Importance of protecting sport values through anti-doping	Question-based interaction	Understanding ethical foundations of anti-doping
15	Applied module	Learning content*	Towards the attainment of clean sport	Lecture	Reinforcement of knowledge
16	Applied module	Prerequisites of clean sport education	Survey data on perceptions of clean sport importance (UNIVAS-Japan)	Data presentation	Recognizing shared values about clean sport
17	Applied module	Survey results on attitudes toward doping*	Survey data on attitudes toward doping behaviors (UNIVAS-Japan)	Data interpretation	Understanding common perceptions among students
18	Applied module	Recognition of the social environment	Identifying people surrounding the athlete	Self-reflection time	Understanding social influences on decision making
19	Applied module	Scenario introduction	QR code video introduction for scenario 3 “The personal best barrier” learning	Video viewing	Preparation for scenario analysis
20	Applied module	Presentation of decision-making materials	Review the text for scenario 3 “The personal best barrier"	Scenario analysis	Examining doping-related decision making
21	Applied module	Examination of social pressure*	Identifying individuals who may encourage doping	Self-reflection time	Understanding social pressure in sport
22	Applied module	Presentation of survey results	Survey results related to responses to scenario 3 “The personal best barrier” (UNIVAS-Japan)	Data interpretation	Comparing personal and group perspectives
23	Applied module	Consideration of response strategies*	Considering how to respond to people encouraging doping	Self-reflection time	Exploring action strategies
24	Applied module	Summary—what you have learned	Review of learning content and key messages	Reflection	Learning integration

* Indicates elements newly added or expanded in the revised lesson (Pilots 3–4).

**Table 5c:** Final instructional structure after iterative refinement.

No	Module	Phase	Content	Learning activities	Objective
1	Foundation module	Introduction	Clean Sport Education Title	Lecture introduction	Presentation of lesson theme
2	Foundation module	Presentation of lesson content*	① What is clean sport, ② Education based on competition level and role, ③ The importance of clean sport	Lecture	Overview of lesson structure
3	Foundation module	Values of sport	List the values of sport based on experience in playing, watching, learning, and supporting sport	Question-based interaction	Understanding the values of sport
4	Foundation module	Examples of sport values	Respect, effort, cooperation, fun, courage, challenge	Lecture	Recognizing concrete examples of sport values
5	Foundation module	Clean sport concept	Integrity in sport and the importance of protecting sport values	Lecture explanation	Understanding the concept of clean sport
6	Foundation module	Philosophy of clean sport education	Role of clean sport education in promoting sport values and ethical behavior	Lecture explanation	Understanding the philosophy of clean sport education
7	Foundation module	Learning content*	Transition to the topic: Education based on competition level and role	Lecture	Connecting lesson components
8	Foundation module	Competition level and athlete categories	Athlete categories based on competition level (WADA-ISE framework)	Lecture explanation	Understanding differences in athlete environments
9	Foundation module	Anti-doping education topics	11 topics in anti-doping education (WADA-ISE framework)	Lecture	Understanding the scope of anti-doping education
10	Foundation module	Presentation of learning objectives	‘Be aware of,’ ‘understand,’ ‘Be capable of doing’ (WADA-ISE framework)	Lecture	Sharing learning objectives
11	Foundation module	Learning content*	Transition to the topic: The importance of clean sport	Lecture	Connecting lesson components
12	Foundation module	Definition of doping	Meaning of doping and prohibited methods	Question-based interaction	Understanding what constitutes doping
13	Foundation module	Definition of doping	Doping and the 11 anti-doping rule violations (WADA Code)	Lecture explanation	Understanding anti-doping rules
14	Foundation module	Definition of doping	Additional explanations of what is doping (WADA framework)	Question-based interaction	Deepening understanding of anti-doping rules
15	Foundation module	Anti-doping philosophy	Importance of protecting sport values through anti-doping	Question-based interaction	Understanding ethical foundations of anti-doping
16	Foundation module	Summary—what you have learned	Review of key ideas about clean sport and anti-doping	Reflection	Reinforcement of knowledge
17	Applied module 1	Introduction	Introduction to clean sport scenario: The personal best barrier	Lecture introduction	Preparation for scenario learning
18	Applied module 1	Learning content*	Importance of clean sport, performance pressures, impacts of doping, and actions to promote and realise clean sport	Lecture explanation	Understanding the goals of scenario learning
19	Applied module 1	Prerequisites of clean sport education	Survey data on perceptions of clean sport importance (UNIVAS-Japan)	Data presentation	Recognizing shared values about clean sport
20	Applied module 1	Survey results on attitudes toward doping	Survey data on attitudes toward doping behaviors (UNIVAS-Japan)	Data interpretation	Understanding common perceptions among students
21	Applied module 1	Scenario introduction	QR code video introduction for scenario 3 “The personal best barrier” learning	Video viewing	Preparation for scenario analysis
22	Applied module 1	Presentation of decision-making materials	Review the text for scenario 3 “The personal best barrier"	Lecture explanation	Examining doping-related decision making
23	Applied module 1	Acceptability and behavioral choices*	Evaluation of acceptability and behavioral choices in the scenario	Lecture explanation	Reflecting on ethical decisions
24	Applied module 1	Presentation of survey results	Survey results related to responses to scenario 3 “The personal best barrier” (UNIVAS-Japan)	Data interpretation	Comparing perspectives
25	Applied module 1	Factors contributing to doping*	Psychological factors influencing doping behavior: gateway theory, moral disengagement	Lecture explanation	Understanding psychological mechanisms
26	Applied module 1	Reflecting through scenarios*	Reflection question: Resisting doping temptation	Self-reflection time	Encouraging ethical judgment
27	Applied module 1	Consideration on action choice*	Handling the situation yourself or seeking support	Self-reflection time	Exploring action strategies
28	Applied module 1	Summary—what you have learned	Review of learning content and key messages	Reflection	Learning integration
29	Applied module 2	Introduction*	Introduction to clean sport scenario: The gap with a rival	Lecture introduction	Preparation for scenario learning
30	Applied module 2	Learning content*	Recognizing the importance of anti-doping, risks of supplements, and ethical decision-making	Lecture explanation	Understanding scenario learning objectives
31	Applied module 2	The importance of anti-doping education*	Survey results about anti-doping knowledge	Data presentation	Understanding the importance of education
32	Applied module 2	The importance of anti-doping*	Importance of following anti-doping rules	Data presentation	Understanding athlete responsibility
33	Applied module 2	The gap with a rival*	Scenario introduction	Scenario analysis	Understanding other-referential decision situations
34	Applied module 2	The gap with a rival*	Scenario description involving suspected doping	Scenario analysis	Examining ethical dilemmas
35	Applied module 2	Acceptability and behavioral choices*	Evaluation of acceptability and behavioral choices in the scenario	Self-reflection time	Reflecting on ethical decisions
36	Applied module 2	Reflecting through scenarios*	Survey results related to scenario responses (UNIVAS-Japan)	Data interpretation	Comparing perspectives
37	Applied module 2	Risks of supplement use*	The risk of prohibited substances contaminating supplements and cases of doping	Lecture explanation	Understanding contamination risks of supplement use
38	Applied module 2	Reflecting through scenarios*	Reflection on responses to others’ doping	Self-reflection time	Encouraging ethical judgment
39	Applied module 2	Consideration on action choice*	Handling the situation yourself or seeking support; actions for realizing clean sport	Self-reflection time	Exploring action strategies
40	Applied module 2	Summary—what you have learned*	Review of scenario learning	Reflection	Learning integration

* Indicates elements newly added or expanded in the revised lesson (after iterative refinement).

#### 3.7.2 Pilots 1–2: initial implementation and identification of issues

The first two pilot implementations were conducted to examine the feasibility of the initial instructional design and to explore ways of introducing anti-doping education to university students in a sport sciences department. Although many students were involved in sport, not all were competitive athletes who would realistically be subject to doping control procedures. Therefore, the lesson was designed to address anti-doping issues within a broader sport context. The initial lesson combined lecture-based instruction on clean sport and anti-doping with a scenario-based activity using Scenario No. 3 (“The personal best barrier”), in which students considered how people surrounding an athlete might influence doping-related decision-making and how they themselves might respond. Worksheets were used to support explanation and reflection during these activities, and the instructional process was video-recorded to enable post-class review by the instructional team. The structure of the initial lesson is presented in Table 5a.

Classroom observations and post-class reflections during Pilot 1 identified several issues in the initial implementation. First, some students had difficulty understanding how to complete parts of the worksheet, indicating that the task instructions were not sufficiently clear. Second, the worksheet activities took longer than anticipated, resulting in waiting time because of differences in students’ working pace. Third, the small-group discussions were less active than expected, as many students tended to work individually on the worksheet rather than engage in sustained peer discussion. In addition, completion rates were lower for the final worksheet task, which asked students to describe how they would persuade a person likely to justify or encourage doping, than for the earlier worksheet items. The worksheet responses further illustrated the need for clearer prompts. In the “List the people around athletes” section of Supporting Clean Sport, students in both Pilots 1 and 2 were generally able to identify people surrounding athletes, such as coaches, teammates, family members, rivals, sponsors, and medical or support staff. However, in the subsequent “Persuasion strategy” section, which asked students how they would persuade someone likely to justify or encourage doping, some responses were brief, such as “I would tell them that doping is harmful to health” or “doping violates the rules and is unfair.” Other responses were more developed, for example asking whether the athlete would “really be satisfied with a result that was not achieved through their own effort” or noting that doping could “cause trouble for the people around them.” These examples indicated that the activity was feasible, while also showing that learners needed clearer prompts to move from identifying social influences to articulating concrete response strategies. In response to these observations, several immediate adjustments were made for Pilot 2, including more explicit instructions for completing the worksheet, additional guidance for the final task, supplementary tasks for students who finished the worksheet early, and encouragement for students to talk while writing and to assign roles during discussion. Overall, these observations indicated the need for clearer task guidance, better pacing of the lesson, and a learning structure less dependent on spontaneous group discussion, and they informed the subsequent refinement of the instructional design.

#### 3.7.3 Pilots 3–5: refined instructional design and implementation

Based on the issues identified in Pilots 1 and 2, the instructional materials and lesson structure were revised for the subsequent implementations. Specifically, the worksheet was revised to scaffold learners’ reasoning more explicitly, moving from reflection on sport values and personal experiences to foundational clean sport concepts, scenario analysis, and individual response strategies. To provide a clearer conceptual structure, the revised lesson used in Pilots 3–5 was reorganized around three guiding questions: what clean sport is, why clean sport is important, and how clean sport can be achieved. The Foundational Module was expanded to include explicit content on sport values, the philosophy of clean sport education, athlete categories based on competition level, anti-doping education topics, learning objectives derived from the ISE, and additional explanations of doping and anti-doping rule violations. In the applied module, Scenario No. 3 (“The personal best barrier”) was retained, but the explanation activity was restructured from a group discussion format to an individual worksheet format in which learners articulated their reasoning in writing. This modification was introduced to provide a clearer sequence of tasks and to support a more self-directed mode of learning, enabling learners to work through the worksheet individually, including in independent learning contexts. Overall, these revisions were intended to strengthen the connection between foundational knowledge and scenario analysis and to support more consistent implementation across different class contexts. The structure of the revised lesson is presented in Table 5b.

Pilots 3–5 were implemented using the revised lesson structure. In these implementations, no time was allocated for peer discussion. Instead, the lesson followed the scenario presented in the slides, and learners responded to the prompts by recording their own ideas individually on the worksheet. Pilot 3 was conducted in a psychology course with many non-athlete students, whereas Pilots 4 and 5 were conducted within a department of sport sciences. Accordingly, familiar everyday situations, such as high school club activities and exam preparation, were used in Pilot 3 when explaining Scenario No. 3 (“The personal best barrier”) to enhance personal relevance. Although the explanation activity was conducted in an individual written format, students generally engaged with the worksheet in a focused manner, and few left it entirely blank. No clear signs were observed that students had difficulty following the lesson because they did not understand Scenario No. 3 itself or key concepts such as clean sport. These observations suggested that the individually structured worksheet format was feasible for guiding learners through scenario-based activities, provided that the scenario and worksheet prompts were presented clearly. In Pilots 4 and 5, the instructional team found that the revised lesson was easier to deliver than the 2023 version because, although it covered a wider range of topics, the content was more clearly organized. The individually structured format also eliminated the need to manage stagnation in peer discussions and enabled learners to work through the scenario analysis in a more self-directed manner using the worksheet. Across the revised worksheet implementations, many students appropriately connected clean sport with sport values and personal experience in their worksheet responses.

For example, in the section asking learners to reflect on the values of sport and their own experiences, students referred to values such as “teamwork,” “effort,” and “fair-play spirit,” and some connected these values to experiences of working toward goals with teammates. In the scenario-based worksheet activity, students also identified temptation-related factors such as the desire to achieve an “ideal performance” or overcome the “personal best barrier.” However, when asked how they would respond to such temptation, several responses remained centered on individual self-regulation, such as “not giving in to temptation” or “calming down and thinking carefully,” whereas references to seeking help or support from others were less frequent and tended to be brief.

Taken together, these observations suggested that, in an individually structured scenario-based format, both the scenarios and the worksheet prompts should remain clear and easy to follow, while further refinements were needed to guide learners more explicitly from individual self-regulation toward concrete action choices, including seeking support from others when necessary.

#### 3.7.4 Final program structure

Following the iterative refinements made across the pilot implementations, the final program consisted of the Foundational Module, Applied Module 1, and Applied Module 2 (Table 5c). Compared with the revised lesson used in Pilots 3–5, the final version further clarified the instructional sequence within the Foundational Module by adding transitional components that explicitly linked major lesson sections, including transitions to sections on athletes’ rights and responsibilities under the WADA Code (11–14) and on the importance of clean sport. In addition, the Foundational Module was consolidated into a more coherent sequence that moved from sport values and the philosophy of clean sport education to athlete categories based on competition level (11–14), anti-doping topics and objectives, and the definition of doping and ethical foundations of anti-doping.

Applied Module 1 retained Scenario No. 3 (“The personal best barrier”) as a self-referential doping-related decision-making situation. However, its structure was further elaborated in the final version. New or expanded elements included introductory content on the goals of scenario-based learning, explicit consideration of acceptability and behavioral choices, explanation of psychological factors contributing to doping behavior, reflection on resisting doping temptation, and consideration of action choices, including whether to handle the situation alone or seek support. In addition, Applied Module 2 was newly incorporated to address an other-referential decision-making context using Scenario No. 5 (“The gap with a rival”). This module expanded the program beyond situations in which learners themselves face doping-related decisions and introduced content on anti-doping knowledge, the importance of following anti-doping rules, ethical dilemmas involving suspected doping by another athlete, and the risks associated with supplement use.

Overall, the final program structure extended the revised lesson into a more comprehensive program that addressed both self-referential and other-referential decision-making and more explicitly linked clean sport values with concrete judgment and action in sport-related situations. The final versions of the slides and slide notes are provided in Supplementary Material 3. The slides include speaker scripts in the notes section to support delivery by instructors who had not previously taught the program. The same scripts in the notes section were also used as narration in the on-demand version, which was developed by converting the slides into video format and was published on the official UNIVAS website (39).

#### 3.7.5 Task value

For the Task Value Scale, Cronbach's *α* coefficients for the five subscales (institutional utility value, practical utility value, interest value, public attainment value, and private attainment value) ranged from .78 to .98 across the five pilot implementations, indicating generally acceptable to excellent internal consistency (Table 6). Across the pilot implementations, mean scores for all five subscales ranged from 3.21 to 4.04 and were consistently above the midpoint of the five-point scale.

**Table 6:** Cronbach's *α* coefficients and descriptive statistics for the Task Value Scale across the five pilot implementations

Pilot	Year	N	Institutional Utility			Practical Utility			Interest			Public Attainment			Private Attainment		
			*α*	*M*	*SD*	α	*M*	*SD*	α	*M*	*SD*	α	*M*	*SD*	α	*M*	*SD*
1	2023	37	0.98	3.21	1.28	0.95	3.52	1.09	0.97	3.50	1.09	0.96	3.43	1.19	0.95	3.40	1.15
2	2023	44	0.96	3.74	0.97	0.95	3.91	0.87	0.96	3.76	0.97	0.96	3.67	0.95	0.97	3.78	0.95
3	2024	65	0.92	3.45	0.79	0.78	3.96	0.55	0.95	4.04	0.56	0.94	3.72	0.65	0.93	3.30	0.87
4	2024	34	0.95	3.47	0.90	0.92	3.86	0.79	0.94	3.80	0.63	0.91	3.70	0.70	0.97	3.53	0.83
5	2024	41	0.97	3.36	0.98	0.91	3.52	0.93	0.97	3.48	0.99	0.98	3.43	1.04	0.95	3.39	1.05

α, Cronbach's α coefficients; *M*, Mean; *SD*, standard deviation. Response options ranged from “1 = Strongly disagree” to “5 = Strongly agree.”

## 4 Study 3

### 4.1 Purpose

Study 3 aimed to examine whether participation in the anti-doping educational program developed through the iterative pilot process described in Study 2 was associated with short-term changes in moral disengagement in doping, doping likelihood, and the acceptability of doping-related behaviors. The finalized instructional materials were originally designed for face-to-face classroom instruction. Based on these materials, an on-demand video version of the program was also produced to enable consistent delivery across universities. The study examined whether the educational intervention influenced athletes’ moral disengagement in doping, doping likelihood, and the acceptability of doping-related behaviors. In addition, the effectiveness of the two instructional formats, face-to-face instruction and on-demand video, was compared to determine whether the educational effects differed between delivery modalities.

### 4.2 Survey methods

The inclusion criteria were university athletes belonging to competitive sport teams, clubs, university sport circles, or external sport organizations. Participants were recruited through university faculty members collaborating with the UNIVAS anti-doping education project. Faculty members at participating universities distributed invitations to athletes to participate in the survey and the educational program. The on-demand educational sessions were conducted in January 2025, whereas the face-to-face educational sessions were conducted between September and November 2025. Surveys were administered before and after each educational session using Google Forms.

To evaluate whether the sample size was sufficient to detect the expected effects, a power analysis was conducted using G*Power version 3.1.9.7 (62, 63). The analysis was based on the primary analytical model of a repeated-measures ANOVA examining the interaction between measurement time (pre- vs. post-intervention) and instructional format (face-to-face vs. on-demand). The significance level was set at *α* = .05 and statistical power at 1−*β* = .80. Assuming a small effect size (f = 0.10; *η*^2^ ≈.01) based on Cohen (64), with a correlation between repeated measurements of r = .40 and a nonsphericity correction of *ε* = 1, the required total sample size was estimated to be 552 participants (276 per group). The sample size in Study 3 exceeded this requirement.

### 4.3 Participants

The final analytical sample consisted of 1,241 university athletes. The face-to-face education group comprised 644 athletes representing 46 sports, including 323 males (50.2%) and 321 females (49.8%), with a mean age of 19.9 ± 1.3 years (range = 18–32). The most common sports were volleyball (11.8%), athletics (11.2%), softball (10.2%), baseball (9.2%), American football (7.9%), and soccer (7.9%). Regarding competition level, 9.3% competed at the district level, 32.6% at the prefectural or regional level, 55.7% at the national level, and 2.3% at the international level.

The on-demand education group comprised 597 athletes representing 25 sports, including 443 males (74.2%) and 154 females (25.8%), with a mean age of 20.0 ± 1.0 years (range = 18–25). The most common sports were baseball (33.3%), soccer (28.5%), artistic gymnastics (9.0%), basketball (5.9%), and softball (3.7%). Regarding competition level, 5.9% competed at the district level, 38.4% at the prefectural or regional level, 54.6% at the national level, and 1.2% at the international level.

### 4.4 Measures

#### 4.4.1 Moral disengagement in doping scale

Moral disengagement in doping was measured using the Moral Disengagement in Doping Scale (MDDS) (8). The Japanese version (MDDS-J) developed by Murofushi et al. (74) was used in this study. The original study reported cross-cultural adaptation through back-translation and confirmed its reliability and one-factor structure. The scale consists of six items, including statements such as “Doping is just a way to maximize your potential” and “A player should not be blamed for doping if everyone on the team is doing it.” Responses were recorded on a 7-point Likert scale ranging from 1 (strongly disagree) to 7 (strongly agree). Higher mean scores indicate a stronger tendency toward moral disengagement in doping.

#### 4.4.2 Doping likelihood

Doping likelihood was assessed using a hypothetical doping scenario developed by Kavussanu et al. (8), which has been used in previous studies as a context-specific indicator of athletes’ self-reported likelihood of using a prohibited substance in a hypothetical doping situation (24, 25). Participants were asked to imagine themselves in a situation in which they had the opportunity to use a prohibited substance to improve fitness and enhance performance in an important competition, with only a very small chance of being caught. They then indicated how likely they would be to use the prohibited substance in that situation. Responses were recorded on a 7-point Likert scale ranging from 1 (not at all likely) to 7 (very likely). The scenario was as follows:

*It's the week before the most important competitive game (event) of your season. Your opponents are of similar ability to you. Lately, your performance has been below your best. You don't feel you have the necessary fitness for this competition, and you’re concerned about how you’ll perform. You mention this to a mate, who tells you that he/she uses a substance to enhance fitness. The substance is prohibited for use in sport according to the rules, but there's only a very small chance you’ll be caught*.

#### 4.4.3 Doping-related decision-making scenarios

Acceptability of doping-related behaviors was assessed using the fifteen doping-related decision-making scenarios developed in Study 1 (Figure 1). Participants rated the ethical acceptability of the behavior described in each scenario on a 6-point Likert scale ranging from 1 (not acceptable at all) to 6 (highly acceptable). Higher mean scores indicate greater acceptance of doping-related behavior.

### 4.5 Intervention protocol

Study 3 employed a mixed design, with instructional format (face-to-face vs. on-demand) as a between-subjects factor and measurement time (pre-intervention vs. post-intervention) as a within-subjects factor. The educational intervention was based on the anti-doping educational program developed in Study 2. A unified implementation manual guided instructional procedures to promote consistency across universities. Before the intervention, participants were provided with an explanation of the study procedures and then completed the pre-intervention questionnaire. The instructional session itself lasted approximately 60 min and included the lecture component, scenario-based activities, and a summary of the key points. After the session, participants completed the same questionnaire again to assess changes associated with the educational intervention. Including the explanation of the study procedures and completion of the pre- and post-intervention questionnaires, the total session time was approximately 90 min. For the on-demand education group, the intervention was conducted in January 2025 at 37 universities. Students watched on-demand videos developed from the instructional materials created in Study 2 in classroom settings under the supervision of faculty members. For the face-to-face education group, the intervention was implemented between September and November 2025 at 15 universities. The educational sessions were delivered by four members of the UNIVAS anti-doping education project team using the same instructional slides on which the on-demand videos were based.

### 4.6 Statistical analysis

Internal consistency was evaluated using Cronbach's *α* and interpreted as excellent (≥ 0.9), good (0.8–0.9), acceptable (0.7–0.8), needs improvement (0.6–0.7), or insufficient (< 0.6) (73). Baseline characteristics were compared between the face-to-face and on-demand groups before the main analyses. Independent-samples t-tests were used for continuous variables, including age, and chi-square tests were used for categorical variables, including sex and competition level. Baseline comparisons showed no significant differences between the face-to-face and on-demand groups in age [*t*(1,239) = 1.53, *p* = .127, *d* = 0.09, negligible (64)] or competition level [district/prefectural/regional vs. national/international: *χ*^2^(1) = 0.666, *p* = .414, Cramér's V = .023, negligible (65)]. However, the sex distribution differed significantly between groups [male vs. female: *χ*^2^(1) = 75.841, *p* < .001, Cramér's V = 0.247, small-to-moderate (65)], with a higher proportion of males in the on-demand group than in the face-to-face group. Therefore, to examine changes associated with participation in the educational program while accounting for the imbalance in sex distribution, 2 × 2 × 2 mixed-design ANOVAs were conducted for each outcome variable.

Measurement time (pre-intervention vs. post-intervention) was specified as the within-subjects factor, whereas instructional format (face-to-face vs. on-demand) and sex were specified as between-subjects factors. The time   ×   instructional format interaction was used to examine whether pre-post changes differed between instructional formats, whereas the time   ×   instructional format   ×   sex interaction was used to determine whether sex moderated format-related changes. The time   ×   sex interaction was also examined to determine whether pre-post changes differed by sex irrespective of instructional format. Because the within-subjects factor had only two levels, the sphericity assumption was automatically satisfied and Mauchly's test was not required. Residual normality was assessed by visual inspection of Q-Q plots for each ANOVA model; the plots generally showed some deviations from normality, particularly in the upper tails. Therefore, the findings were interpreted with attention to effect sizes and their 95% confidence intervals. Effect sizes for the main effects and interactions were calculated using partial eta squared, and 95% confidence intervals were reported. Partial eta squared values were interpreted as 0.01 for a small effect, 0.06 for a medium effect, and 0.14 for a large effect (64).

### 4.7 Results

The results of the 2 × 2 × 2 mixed-design ANOVAs for all outcome variables are summarized in Table 7. Effect sizes are reported as partial eta squared.

**Table 7:** Pre-post changes in moral disengagement in doping, doping likelihood, and scenario evaluations by educational format

Variable	Attribute	Face-to-face		On-demand		Time			Time × Educational format			Time × Sex			Time × Educational format × Sex		
		Pre	Post	Pre	Post	*F*	*p*	ηp^2^ [95% CI]	F	*p*	ηp^2^ [95% CI]	*F*	*p*	ηp^2^ [95% CI]	*F*	*p*	ηp^2^ [95% CI]
		M (SD)		M (SD)													
Moral disengagement in doping^a^	Total	1.83 (.95)	1.54 (.83)	1.68 (.86)	1.39 (.72)	181.934	<.001	.128 [.096,.163]	0.639	0.424	.001 [.000,.006]	16.975	<.001	.014 [.004,.029]	0.180	0.671	.000 [.000,.004]
	Male	1.77 (.90)	1.58 (.90)	1.64 (.89)	1.39 (.76)												
	Female	1.90 (.98)	1.50 (.76)	1.81 (.74)	1.40 (.59)												
Doping likelihood a	Total	1.64 (1.24)	1.50 (1.25)	1.59 (1.31)	1.30 (1.00)	31.001	<.001	.024 [.010,.044]	5.240	0.022	.004 [.000,.014]	0.339	0.560	.000 [.000,.005]	1.619	0.203	.001 [.000,.008]
	Male	1.70 (1.29)	1.53 (1.21)	1.61 (1.38)	1.36 (1.13)												
	Female	1.59 (1.18)	1.48 (1.30)	1.53 (1.10)	1.12 (.36)												
Doping-related decision-making scenarios b																	
1. The night before the competition (A)	Total	1.49 (.90)	1.30 (.72)	1.45 (.85)	1.18 (.60)	90.380	<.001	.068 [.044,.097]	2.910	0.088	.002 [.000,.011]	0.399	0.528	.000 [.000,.005]	0.177	0.674	.000 [.000,.004]
	Male	1.50 (.90)	1.31 (.74)	1.44 (.87)	1.19 (.64)												
	Female	1.48 (.91)	1.28 (.70)	1.47 (.78)	1.16 (.49)												
2. Pre-competition pressure (A)	Total	1.38 (.79)	1.27 (.68)	1.31 (.70)	1.16 (.53)	44.448	<.001	.035 [.017,.057]	2.374	0.124	.002 [.000,.010]	3.437	0.064	.003 [.000,.012]	0.110	0.740	.000 [.000,.004]
	Male	1.35 (.75)	1.27 (.65)	1.30 (.70)	1.18 (.58)												
	Female	1.41 (.83)	1.27 (.70)	1.34 (.70)	1.12 (.35)												
3. The personal best barrier (A)	Total	1.36 (.77)	1.26 (.66)	1.32 (.70)	1.18 (.58)	38.108	<.001	.030 [.014,.051]	1.861	0.173	.002 [.000,.009]	0.798	0.372	.001 [.000,.007]	0.639	0.424	.001 [.000,.006]
	Male	1.36 (.77)	1.27 (.67)	1.32 (.73)	1.20 (.64)												
	Female	1.35 (.77)	1.25 (.64)	1.31 (.60)	1.13 (.34)												
4. Competition participation (A)	Total	1.45 (.88)	1.29 (.70)	1.43 (.82)	1.19 (.58)	77.062	<.001	.059 [.036,.086]	4.790	0.029	.004 [.000,.014]	1.401	0.237	.001 [.000,.008]	1.505	0.220	.001 [.000,.008]
	Male	1.47 (.90)	1.31 (.75)	1.41 (.83)	1.20 (.61)												
	Female	1.43 (.86)	1.27 (.65)	1.49 (.78)	1.16 (.48)												
5. The gap with a rival (B)	Total	1.40 (.79)	1.31 (.71)	1.32 (.72)	1.19 (.59)	24.744	<.001	.020 [.007,.038]	0.957	0.328	.001 [.000,.007]	0.601	0.438	.000 [.000,.006]	0.248	0.619	.000 [.000,.005]
	Male	1.43 (.83)	1.32 (.74)	1.35 (.77)	1.21 (.64)												
	Female	1.36 (.74)	1.30 (.68)	1.27 (.55)	1.14 (.37)												
6. Return from injury (A)	Total	1.45 (.86)	1.32 (.74)	1.40 (.83)	1.24 (.67)	38.680	<.001	.030 [.014,.051]	0.566	0.452	.000 [.000,.006]	0.000	0.985	.000 [.000,.000]	0.527	0.468	.000 [.000,.006]
	Male	1.47 (.85)	1.33 (.79)	1.40 (.86)	1.25 (.69)												
	Female	1.43 (.88)	1.32 (.70)	1.41 (.75)	1.23 (.60)												
7. Expectations from spectators (C)	Total	1.39 (.83)	1.29 (.71)	1.29 (.71)	1.16 (.53)	38.892	<.001	.030 [.014,.051]	1.640	0.201	.001 [.000,.008]	3.908	0.048	.003 [.000,.012]	0.497	0.481	.000 [.000,.006]
	Male	1.39 (.84)	1.31 (.74)	1.29 (.73)	1.19 (.59)												
	Female	1.40 (.82)	1.27 (.67)	1.31 (.62)	1.09 (.29)												
8. Considering health (B)	Total	1.84 (1.15)	1.47 (0.88)	1.83 (1.17)	1.40 (0.86)	150.243	<.001	.108 [.078,.141]	2.220	0.136	.002 [.000,.010]	3.177	0.075	.003 [.000,.011]	1.245	0.265	.001 [.000,.008]
	Male	1.89 (1.24)	1.54 (1.01)	1.78 (1.19)	1.41 (.89)												
	Female	1.79 (1.05)	1.40 (.72)	1.95 (1.11)	1.38 (.73)												
9. The value of fair play (B)	Total	1.35 (.75)	1.30 (.71)	1.28 (.68)	1.18 (.57)	22.442	<.001	.018 [.006,.035]	4.568	0.033	.004 [.000,.013]	5.772	0.016	.005 [.000,.015]	0.344	0.558	.000 [.000,.005]
	Male	1.35 (.73)	1.33 (.80)	1.28 (.69)	1.20 (.62)												
	Female	1.36 (.78)	1.27 (.61)	1.31 (.65)	1.11 (.39)												
10. Sponsor temptation (C)	Total	2.07 (1.34)	1.53 (0.95)	2.00 (1.30)	1.46 (0.92)	233.819	<.001	.159 [.124,.195]	1.570	0.211	.001 [.000,.008]	2.854	0.091	.002 [.000,.011]	13.590	<.001	.011 [.002,.025]
	Male	2.22 (1.50)	1.59 (1.05)	1.86 (1.24)	1.42 (.92)												
	Female	1.93 (1.15)	1.46 (.83)	2.40 (1.37)	1.55 (.93)												
11. Advice from a senior athlete (A)	Total	1.31 (.66)	1.27 (.68)	1.30 (.68)	1.20 (.60)	18.052	<.001	.014 [.004,.030]	3.636	0.057	.003 [.000,.012]	2.485	0.115	.002 [.000,.010]	0.129	0.719	.000 [.000,.004]
	Male	1.29 (.61)	1.27 (.68)	1.28 (.70)	1.20 (.64)												
	Female	1.34 (.71)	1.27 (.68)	1.35 (.63)	1.19 (.49)												
12. Whistleblowing (B) *	Total	4.03 (1.89)	4.07 (2.07)	3.97 (1.88)	4.13 (2.00)	3.028	0.082	.002 [.000,.011]	0.986	0.321	.001 [.000,.007]	0.246	0.620	.000 [.000,.005]	0.007	0.933	.000 [.000,.002]
	Male	4.05 (1.91)	4.13 (2.03)	3.95 (1.88)	4.13 (1.99)												
	Female	4.01 (1.86)	4.02 (2.12)	4.01 (1.87)	4.14 (2.02)												
13. Media influence (C) *	Total	3.63 (2.01)	3.41 (1.74)	3.57 (1.98)	3.55 (1.71)	8.659	0.003	.007 [.001,.020]	2.431	0.119	.002 [.000,.010]	1.670	0.196	.001 [.000,.008]	0.349	0.555	.000 [.000,.005]
	Male	3.63 (2.00)	3.44 (1.71)	3.52 (1.97)	3.55 (1.71)												
	Female	3.64 (2.03)	3.38 (1.78)	3.72 (2.01)	3.56 (1.71)												
14. Parental expectations (A)	Total	1.51 (.86)	1.27 (.74)	1.47 (.88)	1.33 (.76)	37.266	<.001	.029 [.013,.050]	0.925	0.336	.001 [.000,.007]	1.087	0.297	.001 [.000,.007]	1.336	0.248	.001 [.000,.008]
	Male	1.52 (.85)	1.28 (.74)	1.46 (.90)	1.35 (.78)												
	Female	1.50 (.87)	1.26 (.75)	1.51 (.81)	1.26 (.71)												
15. Advice from a high school coach (A)	Total	1.38 (.77)	1.24 (.71)	1.37 (.79)	1.32 (.77)	7.633	0.006	.006 [.001,.018]	2.695	0.101	.002 [.000,.010]	0.080	0.777	.000 [.000,.004]	0.404	0.525	.000 [.000,.005]
	Male	1.38 (.76)	1.25 (.73)	1.38 (.84)	1.32 (.72)												
	Female	1.38 (.79)	1.22 (.70)	1.33 (.62)	1.32 (.88)												

Values represent mean (*M*) and standard deviation (*SD*) before and after the intervention. Score range: a (1–7); b (1–6).

(A) self-referential decision-making scenarios (No. 1, 2, 3, 4, 6, 11, 14, and 15), (B) other-referential decision-making scenarios (No. 5, 8, 9, and 12), and (C) normative and value-based orientation scenarios (No. 7, 10, and 13).

* Higher scores indicate greater acceptance of doping-related behavior for Scenarios 1–11 and 14–15, whereas higher scores indicate stronger endorsement of clean sport–related responses for Scenarios 12 and 13.

#### 4.7.1 Moral disengagement

The MDDS-J demonstrated good internal consistency in the present sample (Cronbach's *α* = .83). For moral disengagement, the time   ×   instructional format interaction was not significant (Table 7). However, the main effects of measurement time and instructional format were significant; moral disengagement scores significantly decreased from pre- to post-intervention, and overall scores across pre- and post-intervention were significantly lower in the on-demand group than in the face-to-face group. The time   ×   sex interaction was also significant, with a significantly larger pre–post decrease observed among female athletes than among male athletes, whereas the time   ×   instructional format   ×   sex interaction was not significant.

#### 4.7.2 Doping likelihood

For doping likelihood, the time   ×   instructional format interaction was significant (Table 7). Scores significantly decreased from pre- to post-intervention, and the magnitude of decrease was significantly larger in the on-demand group than in the face-to-face group. The main effects of measurement time and instructional format were also significant; overall scores across pre- and post-intervention were significantly lower in the on-demand group than in the face-to-face group. The time   ×   sex interaction and the time   ×   instructional format   ×   sex interaction were not significant.

#### 4.7.3 Scenario acceptability

(A) Self-referential decision-making scenarios

Across the self-referential scenarios (Scenarios 1, 2, 3, 4, 6, 11, 14, and 15), a significant main effect of measurement time was observed for all scenarios, with scores decreasing from pre- to post-intervention (Table 7). The effect sizes for these time effects ranged from small to medium. The time   ×   instructional format interaction was significant only for Scenario 4 (*p* = .029), with a larger decrease in the on-demand group than in the face-to-face group. No significant time   ×   sex or time   ×   instructional format   ×   sex interactions were observed.

(B) Other-referential decision-making scenarios

For scenarios involving judgments about others’ doping-related behavior (Scenarios 5, 8, 9, and 12), the main effect of measurement time was significant for Scenarios 5, 8, and 9, with scores decreasing from pre- to post-intervention (Table 7). The effect size for the time effect was medium for Scenario 8 and small for Scenarios 5 and 9. Scenario 12, in which higher scores indicated stronger endorsement of reporting a teammate's doping through internal whistleblowing, showed no significant main effect of measurement time. The time   ×   instructional format interaction and the time   ×   sex interaction were significant only for Scenario 9, with a larger decrease in the on-demand group than in the face-to-face group and among female athletes than among male athletes. No significant time   ×   instructional format   ×   sex interactions were observed.

(C) Normative and value-based orientation scenarios

For the normative and value-based orientation scenarios (Scenarios 7, 10, and 13), significant main effects of measurement time were observed for all three scenarios (Table 7). Scores decreased from pre- to post-intervention in Scenarios 7 and 10. Scenario 13 also showed a significant pre–post decrease; however, unlike Scenarios 7 and 10, higher scores in Scenario 13 indicated stronger endorsement of media messages promoting clean sport. The effect size for the time effect was large for Scenario 10, small-to-medium for Scenario 7, and small for Scenario 13. The time   ×   instructional format interaction was not significant for any of these scenarios. The time   ×   sex interaction was significant only for Scenario 7. Scenario 10 showed a significant time   ×   instructional format   ×   sex interaction; descriptively, the decrease was largest among female athletes in the on-demand group, whereas among male athletes, the decrease was larger in the face-to-face group than in the on-demand group.

## 5 Discussion

This study aimed to develop and evaluate a UNIVAS anti-doping education program for university sport in Japan, designed to foster doping-related decision-making competence through a values-based clean sport approach. It also examined whether participation in the program was associated with short-term changes in doping-related psychological indicators. The findings suggest that participation in an instructional approach integrating scenario-based decision-making activities was associated with changes in socio-cognitive and moral factors relevant to doping-related decision-making. In particular, reductions were observed in moral disengagement in doping, doping likelihood, and the acceptability of several doping-related decision-making scenarios. The following sections discuss these findings in relation to the development of the instructional materials (Studies 1 and 2) and the short-term changes associated with the finalized program (Study 3).

### 5.1 Scenario development for doping-related decision-making

Study 1 developed fifteen doping-related decision-making scenarios based on theoretical considerations and real-world disciplinary cases and examined participants’ acceptability ratings and intended behavioral responses. For interpretive purposes, the scenarios were conceptually grouped into three categories according to the primary target of moral decision-making: self-referential decision-making, other-referential decision-making, and normative and value-based scenarios. Although this classification was introduced as an exploratory framework for interpreting the findings, it was broadly consistent with a social cognitive perspective suggesting that moral judgment varies according to the target of action and the surrounding social context (18).

In self-referential scenarios, participants judged their own potential involvement in doping-related behavior under conditions of performance pressure, uncertainty, and interpersonal influence from significant others, such as teammates, parents, senior athletes, or coaches. The predominance of rejection, self-regulated effort, and safer alternatives suggests that judgments about one's own conduct involved self-regulatory moral processes and a sense of personal responsibility (18). At the same time, these scenarios indicate that such judgments were made within situations shaped by perceived social influence and contextual pressure, which is consistent with evidence that doping-related decisions are associated with attitudes, social norms, and self-regulatory factors rather than with a simple, stable willingness to dope (2, 3). This interpretation is also broadly consistent with recent arguments that anti-doping education should be linked to individual-level decision-making and sensemaking in context-sensitive situations (75).

In other-referential scenarios, participants evaluated the conduct of rivals, teammates, or other athletes in situations involving suspected doping, medication use, fair play, and whistleblowing. The generally low acceptability of others’ doping-related behavior, together with the relatively frequent selection of consultation-based and direct engagement responses, suggests that these scenarios elicited judgments focused on fairness, responsibility, and appropriate interpersonal responses rather than passive tolerance. This interpretation is consistent with social cognitive perspectives, which suggest that moral judgment is shaped within social relationships and in light of anticipated consequences (18). Because these scenarios were also informed by theoretically relevant justification processes related to moral disengagement, they may further reflect how participants responded to, or resisted, such justifications when evaluating others’ doping-related behavior, although moral disengagement itself was not directly measured in Study 1 (8, 9, 57, 58).

In normative and value-based scenarios, participants responded to pressures and messages originating from spectators, sponsors, and the media. The pattern of responses suggests that doping-related judgments are shaped not only by immediate behavioral choices but also by broader normative climates surrounding sport, including tensions between performance demands, reputational considerations, and clean sport values. This interpretation is consistent with recent discussions emphasizing the importance of values-based anti-doping education and the need to consider how clean sport values are socially communicated and prioritized (6, 17). At the same time, the contrast among these scenarios indicates that value-based judgments are not uniformly positive, but may vary according to whether the surrounding message supports winning at all costs, reputational compromise, or explicit endorsement of clean sport values.

From an educational perspective, the present scenarios may be meaningful not only as exploratory research materials but also as potential learning tools. Hypothetical scenarios have been used in prior doping research to assess athletes’ judgments, susceptibility, and intentions in context-specific situations (28–30). Asking learners to explain and justify their judgments is also consistent with research on self-explanation and deeper learning (31, 32), as well as with recent arguments that anti-doping education should be more explicitly linked to individual-level decision-making and sensemaking in doping-related situations (75). Although findings from dilemma-based ethical decision-making interventions should be interpreted cautiously, prior work suggests that such activities may influence young athletes’ doping-related attitudes (76). Accordingly, the scenarios developed in this study may provide a theoretically informed foundation for future intervention research and for educational approaches targeting doping-related decision-making competence.

### 5.2 Development of the anti-doping educational program

In Study 2, two scenarios were selected from the fifteen doping-related decision-making scenarios developed in Study 1, one representing self-referential decision-making and the other representing other-referential decision-making, and these scenarios served as the basis for the final educational program. Through repeated pilot implementations, the program was progressively refined from an initial face-to-face format incorporating group discussion into a more structured format centered on individual worksheet-based explanation activities. As a result, the materials appeared to become more consistently implementable across different educational contexts. In this respect, the program may be viewed as translating the educational goals of values-based anti-doping education, particularly the development of decision-making competence and anti-doping literacy, into a format that could be practically delivered in university sport settings (6). From an educational perspective, the revised program combined scenario-based learning with explanation activities, thereby linking abstract clean sport concepts to concrete decision-making situations. Scenario-based activities encouraged learners to consider realistic and ethically complex situations in sport, whereas the explanation activities required them to articulate the reasons for their judgments. Such a structure is consistent with self-explanation research, which shows that explaining and justifying one's thinking can promote deeper processing and understanding (32, 34, 70, 71).

In addition, mean scores for all five dimensions of task value were above the midpoint of the scale. According to task value theory, perceiving learning content as useful, interesting, and personally meaningful is associated with engagement and motivation (36). In Study 2, the combination of foundational content, scenario analysis, and explanation activities situated in realistic sport contexts may have supported learners’ recognition of the value of the learning content. In particular, asking learners to articulate the reasons for their judgments may have contributed to this process, given previous findings that self-explanation promotes deeper understanding (32, 34, 70, 71). However, because the Task Value Rating Scale captures learners’ perceived value of the class rather than actual changes in knowledge or attitudes, these findings should be interpreted as indicators of the perceived value and feasibility of the materials during the pilot stage rather than as evidence of educational effectiveness. Short-term changes associated with the finalized program were examined separately in Study 3.

The pilot implementations also yielded important implications for the final version of the materials. Although the individually structured format was useful for self-directed progression and consistent delivery, the scenarios needed to remain short, easy to follow, and personally relatable for learners. It was also meaningful to include supplement use and supplement-related risks (41–55), as these are realistic concerns for university athletes. Previous research has shown that sport supplement beliefs are associated with supplement use and with doping-related perceptions and susceptibility (77). Hurst et al. further showed that the association between supplement use and doping use was mediated by sport supplement beliefs and moderated by moral values and moral identity (78). In addition, the materials addressed mechanisms of moral disengagement in doping, which have been identified as processes that justify doping-related behavior (8). At the same time, worksheet responses showed that many students emphasized handling doping-related temptation on their own, whereas relatively few referred to seeking support from others. These findings indicated the need to explicitly address, in the final version of the materials, the importance of consulting others and critically examining the basis for one's judgment when facing difficult decisions.

### 5.3 Short-term changes associated with the program across instructional formats

Study 3 explored whether participation in the anti-doping program developed in Study 2 was associated with short-term changes in doping-related psychological indicators, while comparing face-to-face instruction with an on-demand video format. Moral disengagement in doping decreased significantly from pre- to post-intervention, with a relatively large effect size for measurement time. The time   ×   instructional format interaction was not significant, indicating that reductions were observed across both formats. The time   ×   sex interaction was significant, but its effect size was small. Doping likelihood also decreased in both groups, and the reduction was slightly larger in the on-demand group, although the effect size for this interaction was small; no corresponding sex-related interaction was observed for doping likelihood. Thus, including sex as an additional between-subjects factor did not substantially alter the interpretation that both psychological outcomes showed short-term decreases following the program. These changes are meaningful because moral disengagement has been identified as a key cognitive mechanism through which athletes justify doping-related behavior (8–10), while doping likelihood, although assessed with a single hypothetical scenario item, has been examined as a proximal indicator related to moral disengagement in doping (8) and doping-related intentions and decision-making processes (2, 3, 8). In anti-doping education, even small shifts in proximal psychological indicators may have practical relevance when the immediate aim is to influence ethical judgment, justification of prohibited behavior, and perceived likelihood of doping. From this perspective, the findings suggest that the materials developed in Study 2 were associated with short-term changes in proximal psychological indicators relevant to the socio-cognitive dynamics of doping across both delivery formats.

The scenario acceptability ratings provide further insight into how the program was associated with athletes’ judgments in context-specific doping-related situations, although the pattern of change differed across the three scenario categories. In (A) self-referential decision-making scenarios, scores decreased across all scenarios, suggesting reduced acceptance of doping-related behaviors when participants evaluated their own possible decisions. Although the time   ×   instructional format interaction was significant for Scenario 4, the effect size was very small, indicating that changes in this category were largely similar across face-to-face and on-demand formats. In (B) other-referential scenarios, decreases were observed for Scenarios 5, 8, and 9, suggesting reduced acceptance of doping-related behaviors when participants evaluated situations involving other athletes. For Scenario 9, the decrease was larger in the on-demand group than in the face-to-face group and larger among female athletes than among male athletes, although both effects were small. In contrast, Scenario 12, which assessed the acceptability of reporting a teammate's doping through internal whistleblowing, showed no significant time effect. In (C) normative and value-based scenarios, scores decreased for Scenarios 7, 10, and 13. For Scenarios 7 and 10, these decreases reflected reduced acceptance of doping-related behavior. However, for Scenario 13, the decrease reflected a slight reduction in the acceptability of media messages promoting clean sport, because higher scores in this scenario indicated stronger endorsement of such messages. Scenario 10 also showed a small time   ×   instructional format   ×   sex interaction, suggesting that responses to sponsor-related temptation varied somewhat by delivery format and sex. Overall, the scenario findings indicate modest changes in scenario-based judgments across both instructional formats, but these changes were not uniform across all items and should be interpreted according to each scenario category and normative meaning.

These findings are meaningful when interpreted alongside the broader psychological outcomes. Scenario-based approaches have been used to present realistic and ethically ambiguous sport situations (28–30) and to examine doping-related decision-making processes in sport contexts (38). In the present study, changes in scenario acceptability, together with decreases in moral disengagement and doping likelihood, may reflect short-term shifts in proximal psychological processes involved in doping-related judgment. Moral disengagement mechanisms are key processes through which athletes justify unethical behavior in sport (8, 10), and such cognitive processes are linked to doping intentions (2, 3). From the perspective of the IMDB (5), doping-related decisions may emerge gradually through interactions among justification, perceived norms, and situational pressures. Although many effects in Study 3 were small, the pattern of changes across moral disengagement, doping likelihood, and scenario-based judgments is consistent with short-term changes in early psychological processes relevant to ethical decision-making. These findings support the value of anti-doping education that goes beyond rule-based knowledge transmission (79–81) and helps athletes interpret, evaluate, and respond to ethically complex situations in sport (6). Thus, the scenario-based materials developed through Studies 1 and 2 may provide a practical framework for values-based anti-doping education in Japanese university sport. However, the findings should still be regarded as preliminary evidence of short-term changes associated with the program rather than evidence of behavioral or long-term effects.

### 5.4 Study limitations and future directions

Several limitations should be acknowledged. First, the Study 1 survey included university athletes and student ASP, and the pilot implementations in Study 2 included student ASP and, in some classroom settings, non-athlete students. This reflected the authentic educational context of Japanese university sport, where athletes may learn alongside peers and support personnel; however, the scenario ratings from Study 1 and the learning responses from Study 2 may not fully represent the perspectives of competitive athletes. Although Study 3 analyzed university athletes, future research should compare athletes, student ASP, and non-athlete students to determine whether scenario acceptability and learning responses differ by participant role. Second, the instructional refinement process in Study 2 was conducted through pilot implementations in authentic university classroom settings. Some of the information used to guide revisions was derived from classroom observations, instructor reflections, and consensus discussions among the project researchers rather than from formal qualitative analysis. Although representative worksheet responses are presented to illustrate the refinement process, these examples should be regarded as illustrative rather than as systematically coded qualitative data. Third, Study 3 did not include a no-intervention control group and relied on a pre-post design. Therefore, the observed short-term changes cannot be interpreted as definitive causal evidence of program effectiveness, as they may partly reflect testing or maturation effects. Fourth, direct comparisons between instructional formats in Study 3 should be interpreted cautiously. Participants were not randomly assigned to instructional formats, and the on-demand and face-to-face implementations were conducted approximately 8–10 months apart. Thus, any apparent differences between formats may have been influenced not only by delivery modality itself but also by history effects, selection bias, differences in sex distribution, sport composition, participant characteristics, and institutional context across universities. Although the analyses accounted for sex, unmeasured group differences may still have influenced the findings. Future research should examine whether program-associated changes differ across specific sports, event types, and competitive contexts using designs that allow more precise classification of sport-related characteristics. Fifth, Study 3 focused on short-term changes immediately after the intervention, and the long-term sustainability of these changes remains unclear. Future research should examine whether participation in the program is associated with sustained changes in moral disengagement, doping likelihood, and scenario-based judgments over time. Sixth, the Study 3 outcome measures assessed moral disengagement, doping likelihood, and scenario-based acceptability judgments. Although these are theoretically relevant psychological indicators, they do not directly assess actual doping behavior. In addition, doping likelihood was assessed using a single hypothetical scenario item, which may not fully capture the multidimensional nature of doping intentions across different situations. Because the study addressed a socially sensitive topic and responses were linked across time points for pre-post comparisons, socially desirable responding cannot be ruled out, particularly at post-intervention. Finally, because the present study was conducted within the context of university sport in Japan, cultural and institutional characteristics of the Japanese university sport system may have influenced the findings. Caution is therefore needed when generalizing the results to other cultural or institutional contexts. Future research should examine whether similar findings are observed in other national sport systems and educational settings. It will also be important to investigate how specific instructional components, such as scenario analysis, explanation activities, and support-seeking elements, contribute to learning and decision-making in both face-to-face and on-demand formats.

## 6 Conclusion

This study developed and preliminarily evaluated a values-based anti-doping education program for university sport in Japan. Building on theoretical frameworks and real-world disciplinary cases, it developed scenario materials representing doping-related decision-making situations relevant to university athletes. These scenarios were incorporated into instructional materials that were iteratively refined through pilot implementations in university classroom settings, resulting in a structured educational program integrating scenario analysis and explanation activities. Evaluation of the finalized program across face-to-face and on-demand formats provided preliminary evidence of short-term program-associated changes in psychological variables relevant to doping-related decision-making. Taken together, these findings suggest that a values-based, scenario-based educational approach may provide a feasible and potentially useful framework for anti-doping education in university sport and support the development of ethically grounded decision-making in realistic sport contexts. These findings contribute to the development of evidence-informed anti-doping education by offering a theoretically grounded and practically implementable instructional model for Japanese university sport settings.

## Data Availability

The raw data supporting the conclusions of this article will be made available by the authors upon reasonable request, without undue reservation.
