# Supplementary material for: Development and evaluation of a values-based anti-doping education program for university sport in Japan: the UNIVAS clean sports intervention
Source: Front Sports Act Living. 2026 Jun 22;8:1835205. doi: 10.3389/fspor.2026.1835205 (PMC13333625; doi:10.3389/fspor.2026.1835205)
Supplement: Supplementary file 1 [file Supplementaryfile1.docx]

Supplementary Material 1

# Worksheet Used in Study 2

This worksheet was used in the pilot instructional sessions described in Study 2 to support learners’ understanding of clean sport concepts, analysis of social influences surrounding athletes, and articulation of response strategies in doping-related situations. Blank items (A–I) were completed by learners during the lesson based on the instructional slides and lecture explanation. The original worksheet was developed in Japanese. The English version presented here was prepared by the authors to facilitate readers’ understanding and does not represent a separately validated English-language version of the worksheet.

**Clean Sport Education Worksheet**

**Student No.**（　　　　　　　　　　　　　）**Name**（　　　　　　　　　　　　　　）

**1. What is Clean Sport?**

- **Reflect on the values of sport and your own experiences**

| **Values of Sport** | **Your Experiences** |
| --- | --- |
|  |  |

- **Fill in the blank based on the explanation in the lecture.**

**□What is clean sport?（**from the slide materials)

Clean sport refers to a state in which the (A      ) of sport and of each individual are protected, and people have a positive influence on one another.

**□What is Clean Sport Education?（**from the slide materials)

Clean sport education aims to develop people who play a role not only in sport, but also in (B      ), by (C      ) the values of sport to create a better society and future.

**2. The importance of clean sport**

- **Fill in the blank based on the explanation in the lecture.**

**□What is Doping?**

Doping refers to the intentional use of prohibited substances (drugs) or prohibited methods

in order to enhance (D      ).

It also includes possessing (E      ) substances, illegally “trading” them, “concealing” them, forcing others to use them, or (F      ) doping control tests.

**□The philosophy of anti-doping**

Sport exists under a set of (G      ), and is sustained when all people involved in sport —those who compete, watch, support, and contribute — are (H      ).

⇒ Anti-doping activities are essential to protect the (I      ) of sport.

**3. Supporting Clean Sport**

- List the people around athletes.

- Among the people around athletes, write the top three who might be most likely to justify or encourage doping. For each, explain your reason.

| **Rank** | **Person** | **Reason** |
| --- | --- | --- |
| **1st** |  |  |
| **2nd** |  |  |
| **3rd** |  |  |

- How would you persuade someone who is likely to justify or encourage doping?
- If your approach differs depending on the person, clearly explain who you would persuade and how.

| **Persuasion strategy** |
| --- |
|  |
