# Supplementary material for: Development and evaluation of a values-based anti-doping education program for university sport in Japan: the UNIVAS clean sports intervention
Source: Front Sports Act Living. 2026 Jun 22;8:1835205. doi: 10.3389/fspor.2026.1835205 (PMC13333625; doi:10.3389/fspor.2026.1835205)
Supplement: Supplementary file 2 [file Supplementaryfile2.docx]

Supplementary Material 2

# Translation of the Task Value Rating Scale (Inada, 2001)

The Task Value Rating Scale was developed by Inada (2001) to assess students’ perceived value of academic learning tasks. The original instrument consisted of 30 items representing five dimensions: institutional utility value, practical utility value, interest value, public attainment value, and private attainment value. In the present study, responses were recorded on a 5-point Likert scale ranging from 1 (strongly disagree) to 5 (strongly agree). Subscale scores were calculated as the mean of the items corresponding to each factor, with higher scores indicating greater perceived task value.

The original scale was developed in Japanese. For the purpose of reporting the scale items in this English-language article, the items are presented here in English translation prepared by the authors. This translation is provided solely to facilitate readers’ understanding of the original Japanese items and does not represent a validated English version of the scale.

**Response scale:**

1 = Strongly disagree

2 = Disagree

3 = Neither agree nor disagree

4 = Agree

5 = Strongly agree

**Factor 1. Institutional Utility Value**

1. Necessary for passing examinations required for my desired occupation.
2. Necessary for achieving my career goals.
3. Required when seeking employment or further education.
4. Useful when trying to obtain employment or enter higher education.
5. Increases my chances of obtaining employment or entering higher education.
6. Important for passing employment or entrance examinations.

**Factor 2. Practical Utility Value**

1. Important for functioning as a member of society in the future.
2. Helps me understand social issues related to my future work.
3. Helps me solve problems that I may face in my future work.
4. Helps me contribute to society through my profession.
5. Can be applied in practical situations in my future work.
6. Related to the actual tasks involved in my desired occupation.

**Factor 3. Interest Value**

1. Enjoyable to learn.
2. Interesting to learn.
3. Allows me to study with genuine interest.
4. Stimulates my curiosity while learning.
5. Gives me a sense of satisfaction while learning.
6. Provides intellectual stimulation while learning.

**Factor 4. Public Attainment Value**

1. Would make others respect me if I know it well.
2. Makes me feel I would become smarter than others by learning it.
3. Makes others see me as capable if I know it.
4. Something I could be proud of or share with others if I learn it.
5. Would make me look impressive if I master it.
6. Makes me feel proud of what I am learning.

**Factor 5. Private Attainment Value**

1. Helps me better understand myself through learning.
2. Helps me discover aspects of myself that I had not noticed before.
3. Helps me become closer to my true self through learning.
4. Makes me more interested in myself as a person.
5. Helps me make use of my individuality.
6. Makes me feel that I grow as a person through learning.

**Reference**

Inada M. An attempt to construct the academic task values evaluation scale (in Japanese). Bull Grad Sch Educ Hum Dev Nagoya Univ (Psychol Hum Dev Sci). 2001;48:83-95. doi:10.18999/nupsych.48.83
