# Supplementary material for: Development and evaluation of a values-based anti-doping education program for university sport in Japan: the UNIVAS clean sports intervention
Source: Front Sports Act Living. 2026 Jun 22;8:1835205. doi: 10.3389/fspor.2026.1835205 (PMC13333625; doi:10.3389/fspor.2026.1835205)
Supplement: Supplementary file 3 [file Presentation1.pptx]

## Slide 1
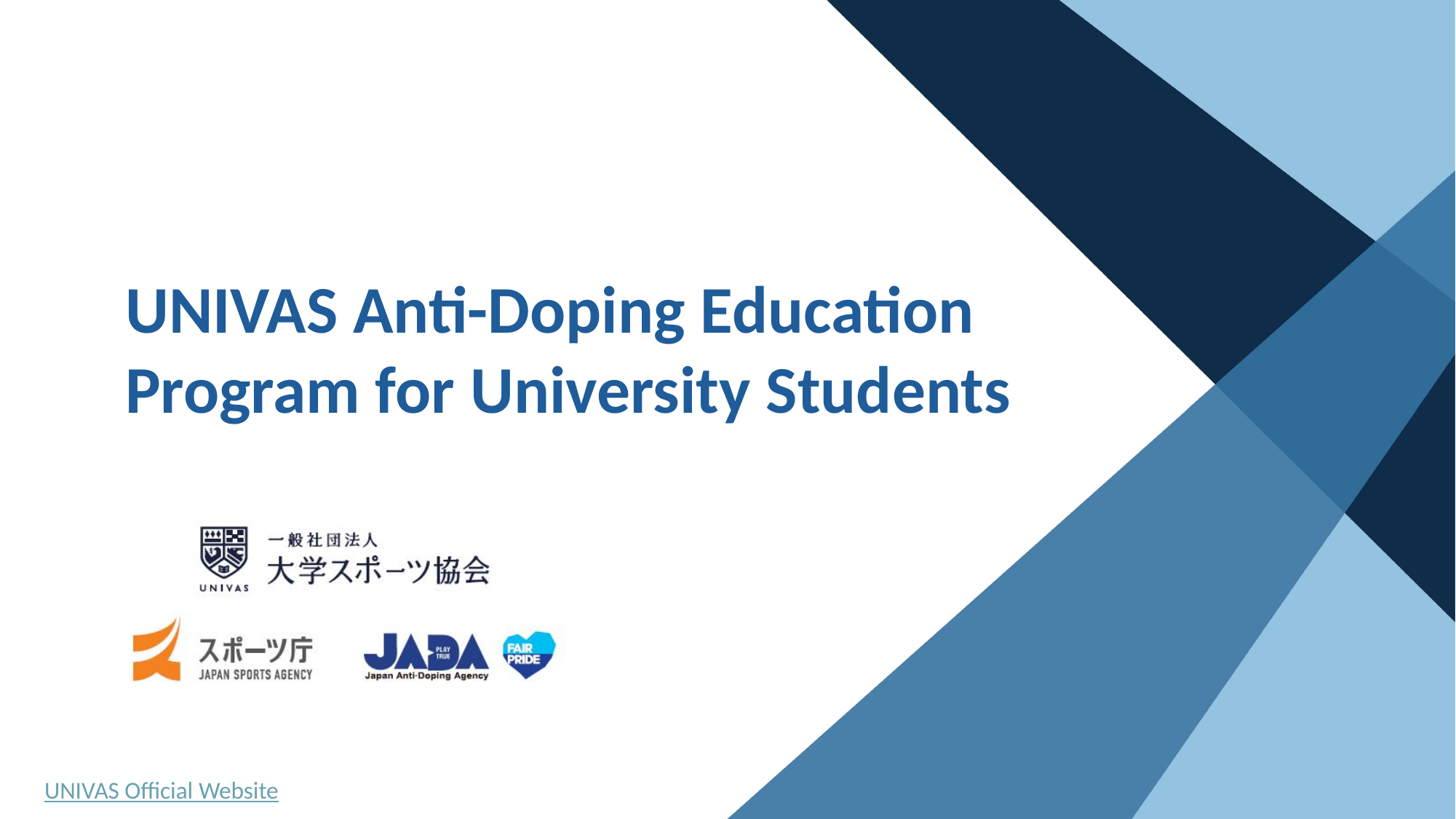

UNIVAS Anti-Doping Education Program for University Students
UNIVAS Official Website

## Slide 2
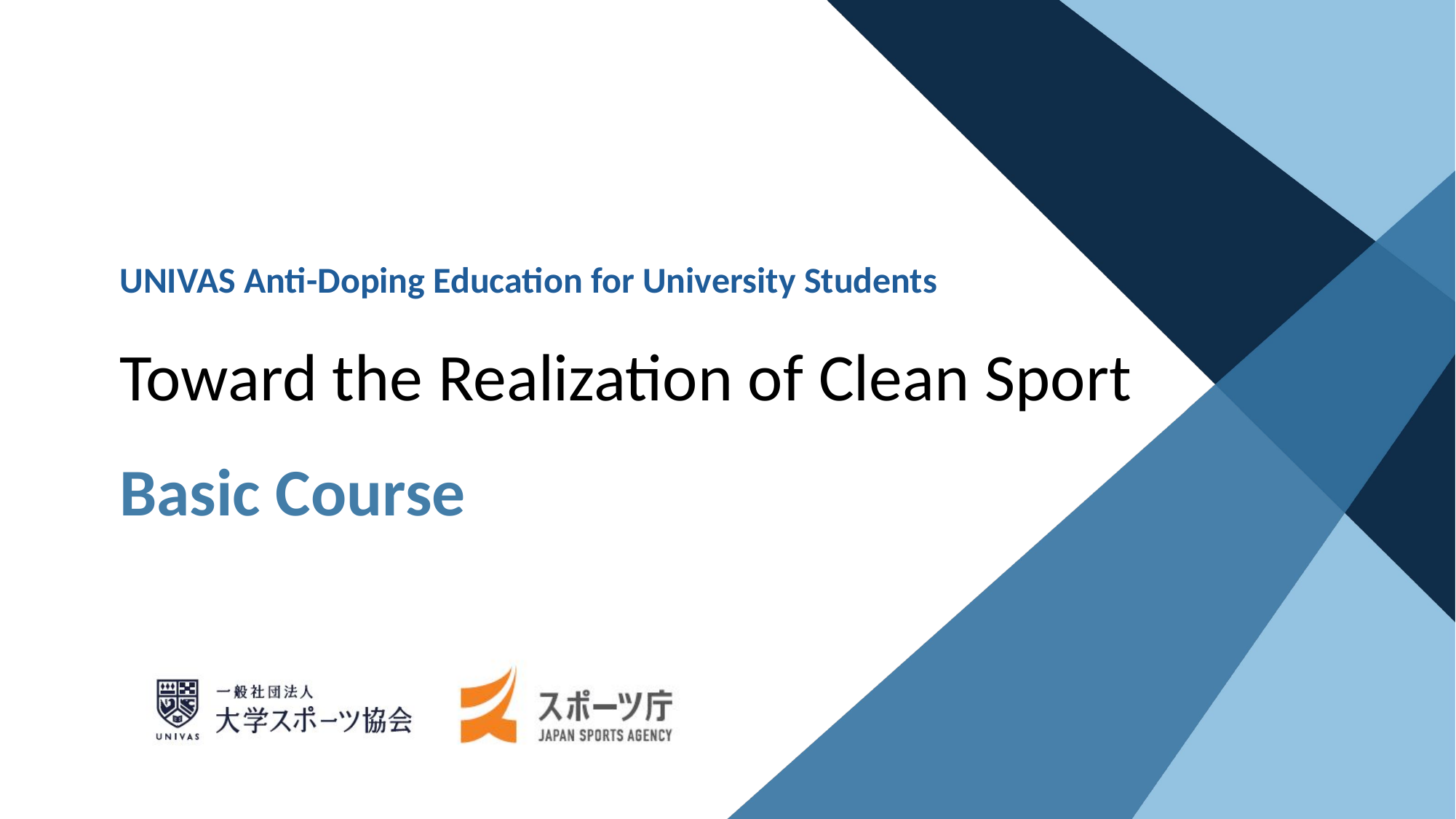

UNIVAS Anti-Doping Education for University Students
Toward the Realization of Clean Sport
Basic Course

## Slide 3
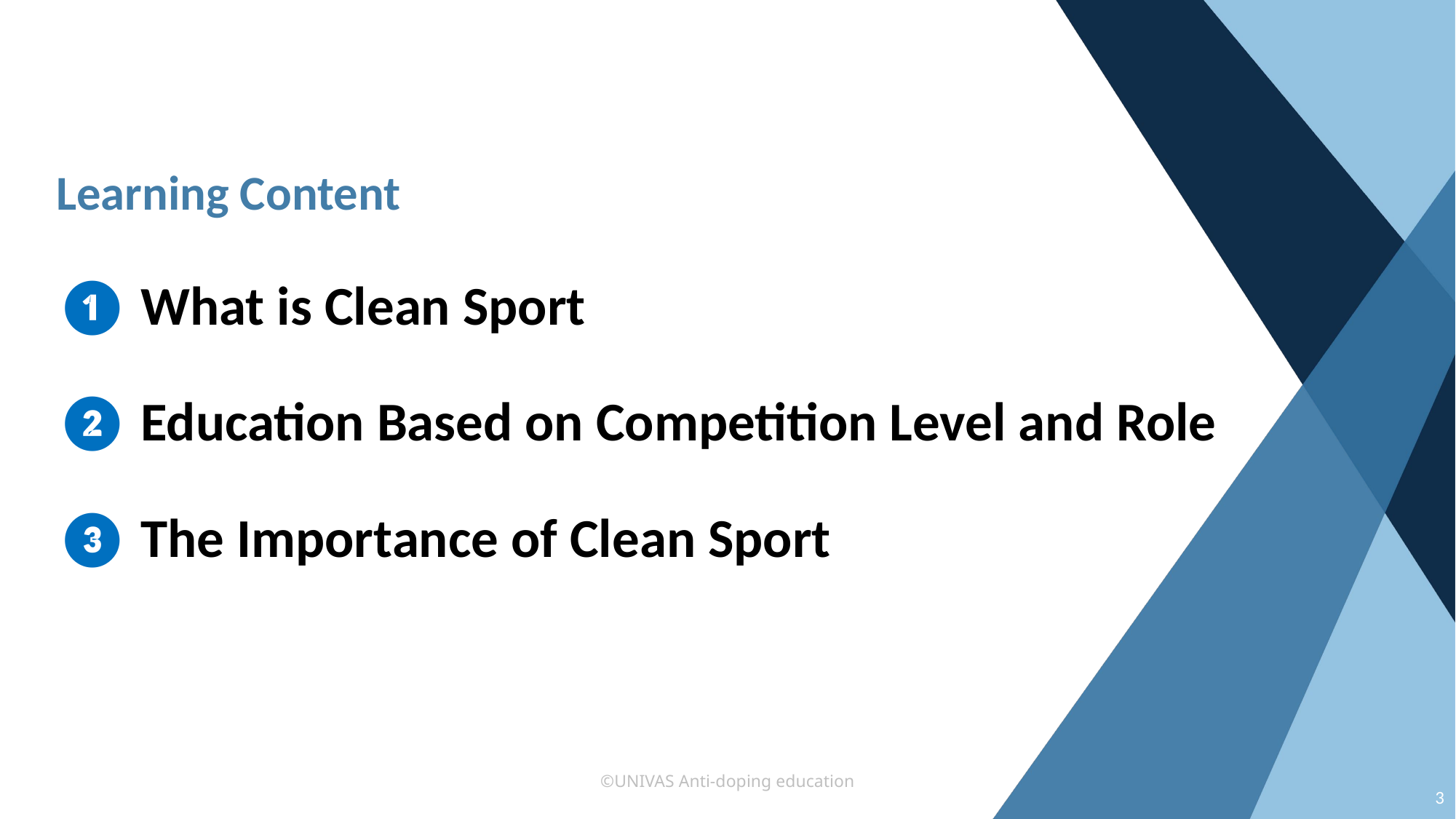

Learning Content
❶ What is Clean Sport
❷ Education Based on Competition Level and Role
❸ The Importance of Clean Sport
©UNIVAS Anti-doping education
2

## Slide 4
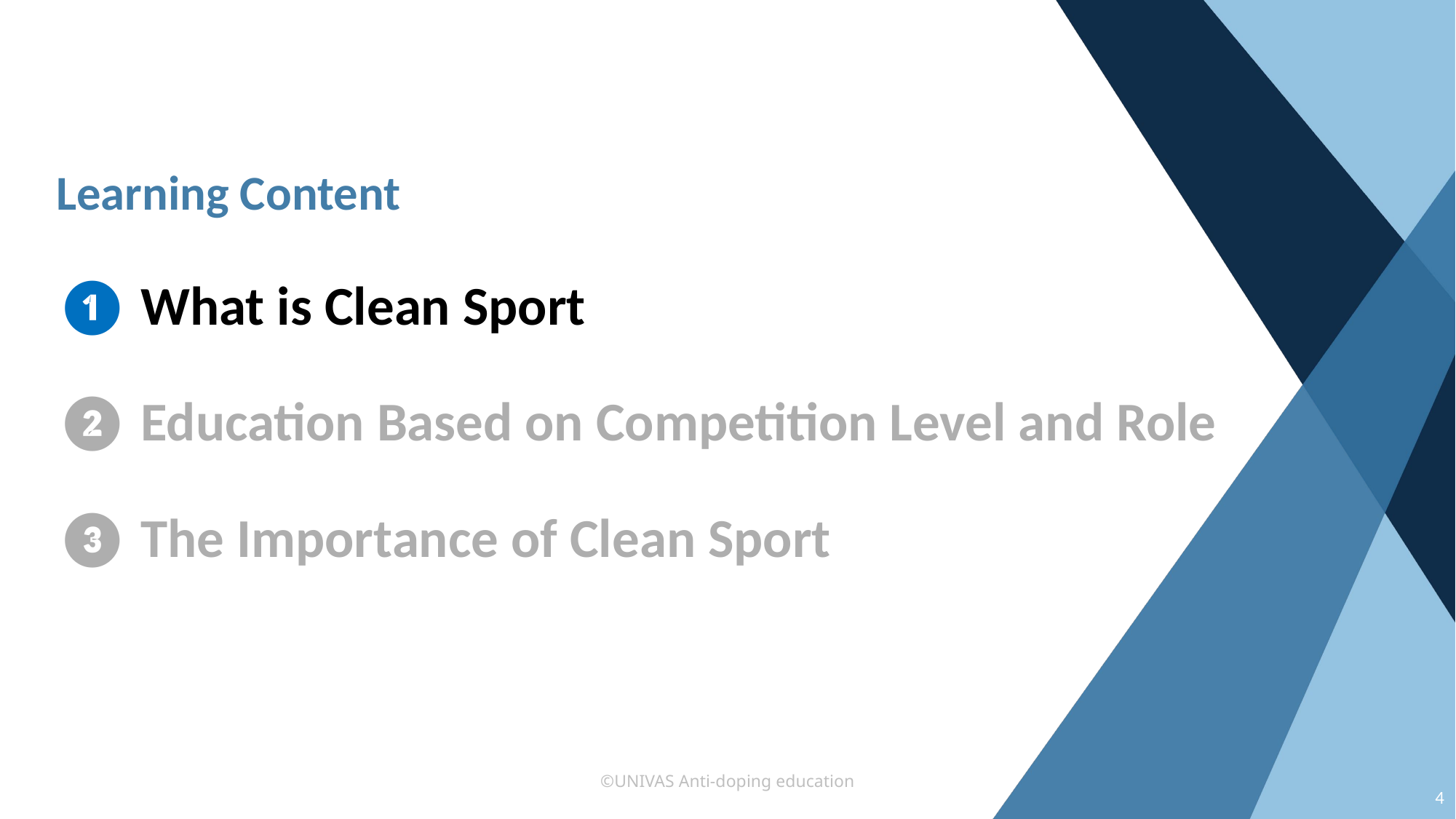

Learning Content
❶ What is Clean Sport
❷ Education Based on Competition Level and Role
❸ The Importance of Clean Sport
©UNIVAS Anti-doping education
3

## Slide 5
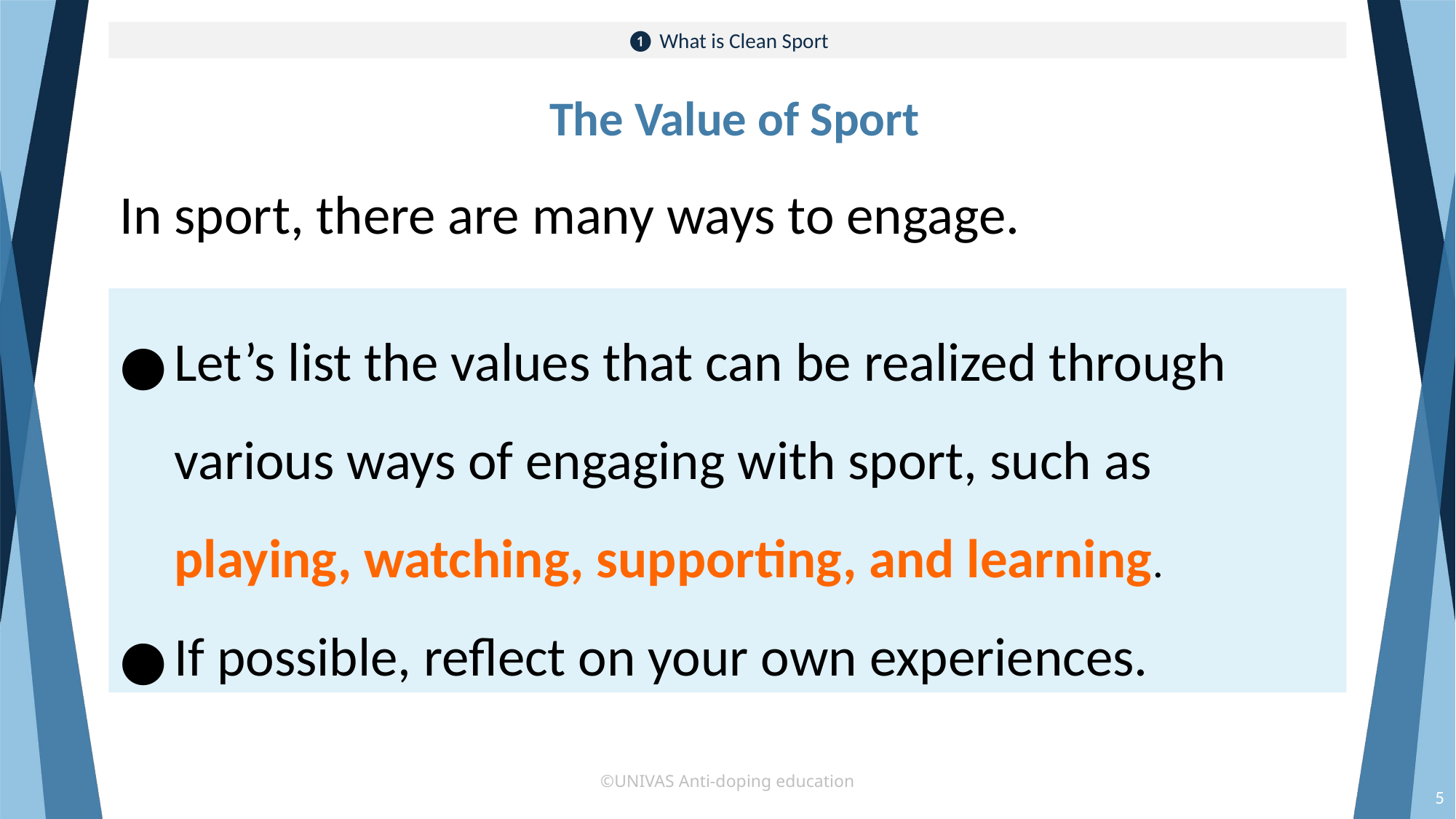

❶ What is Clean Sport
The Value of Sport
In sport, there are many ways to engage.
Let’s list the values that can be realized through various ways of engaging with sport, such as playing, watching, supporting, and learning.
If possible, reflect on your own experiences.
©UNIVAS Anti-doping education
4

## Slide 6
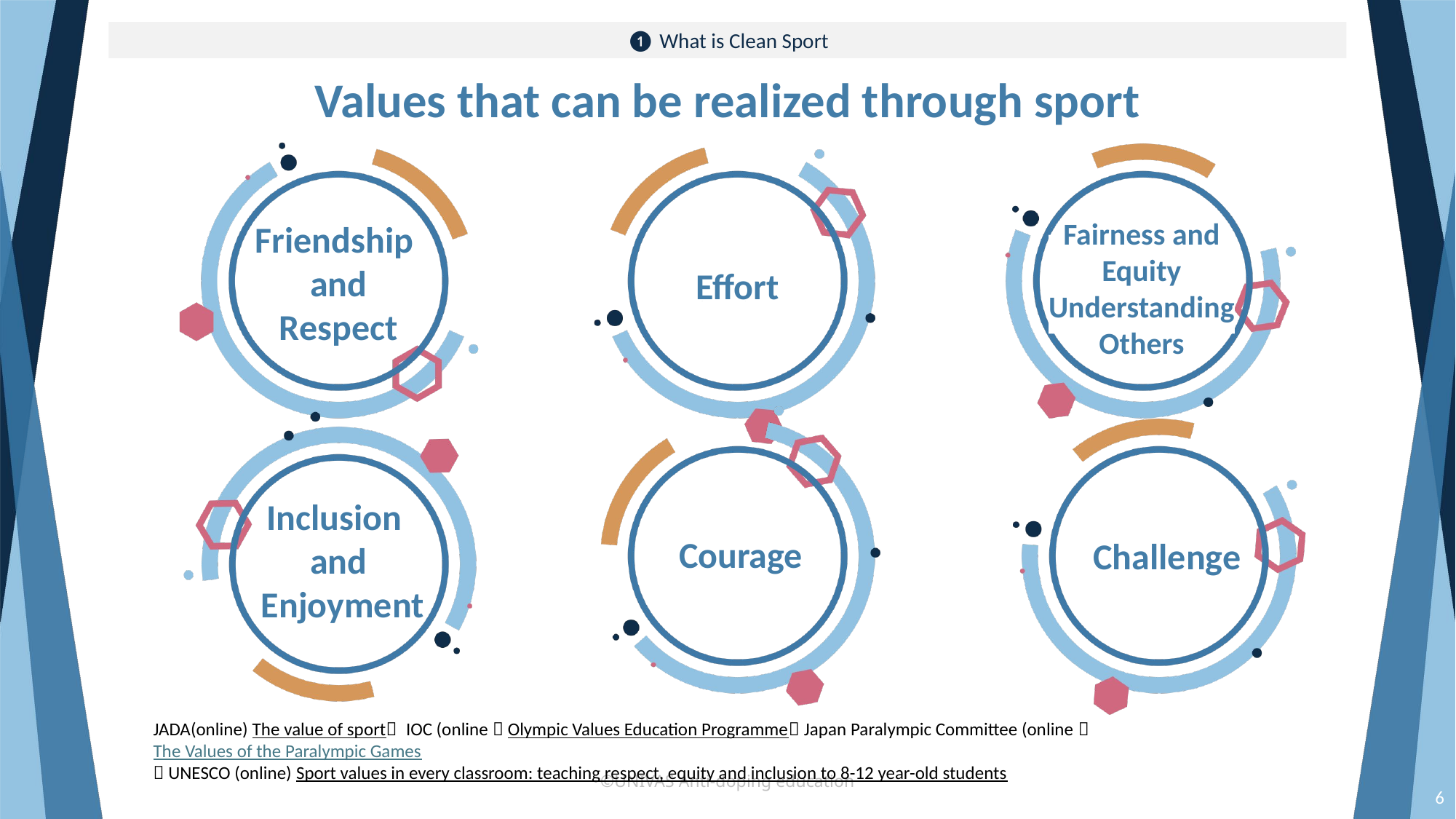

❶ What is Clean Sport
Values that can be realized through sport
Fairness and Equity Understanding Others
Friendship
and Respect
Effort
Inclusion
and
 Enjoyment
Courage
Challenge
JADA(online) The value of sport； IOC (online）Olympic Values Education Programme；Japan Paralympic Committee (online）The Values of the Paralympic Games
；UNESCO (online) Sport values in every classroom: teaching respect, equity and inclusion to 8-12 year-old students
©UNIVAS Anti-doping education
5

## Slide 7
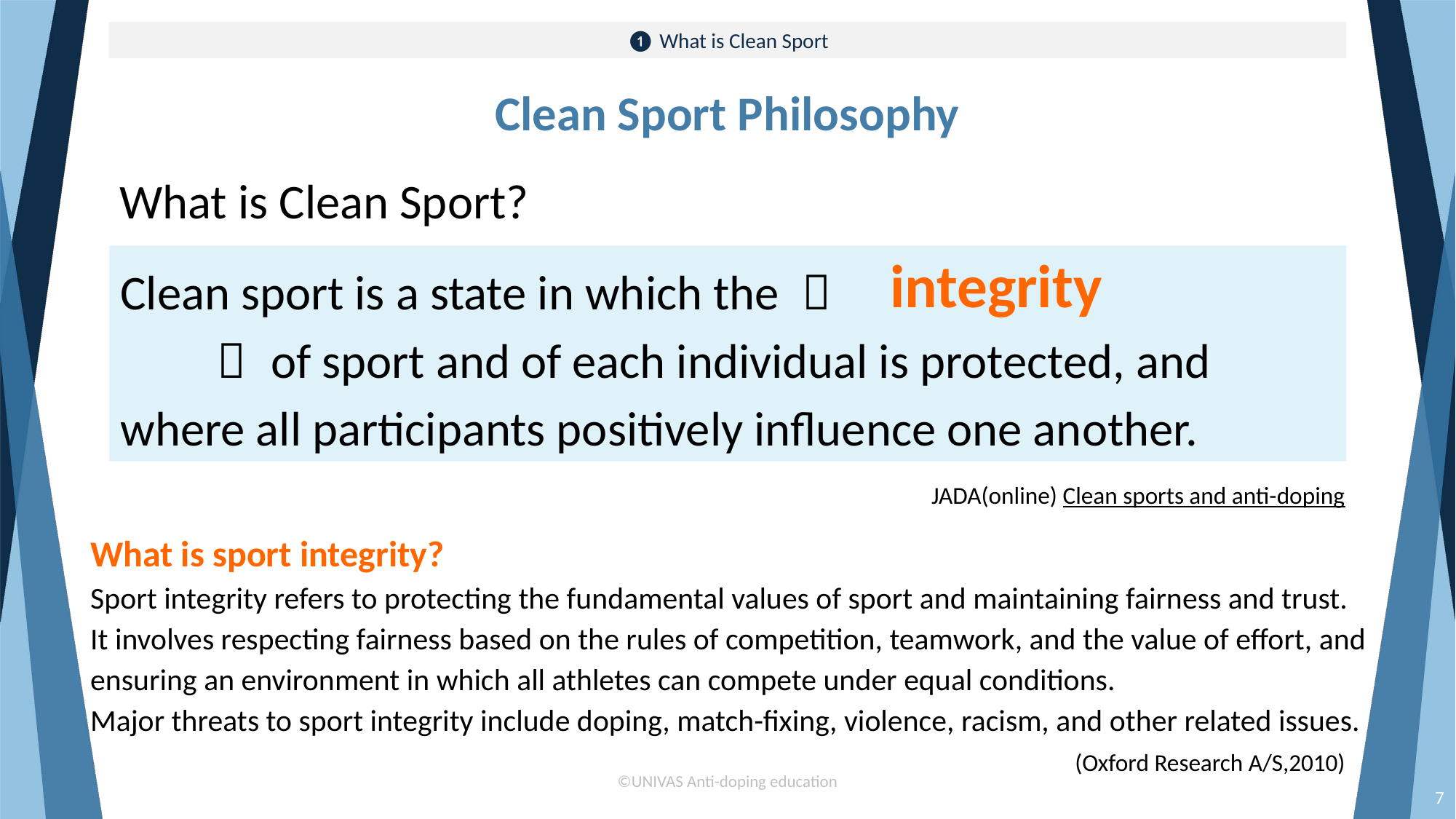

❶ What is Clean Sport
Clean Sport Philosophy
What is Clean Sport?
integrity
Clean sport is a state in which the （　　　　　　　　　　　　） of sport and of each individual is protected, and where all participants positively influence one another.
JADA(online) Clean sports and anti-doping
What is sport integrity?
Sport integrity refers to protecting the fundamental values of sport and maintaining fairness and trust.
It involves respecting fairness based on the rules of competition, teamwork, and the value of effort, and ensuring an environment in which all athletes can compete under equal conditions.
Major threats to sport integrity include doping, match-fixing, violence, racism, and other related issues.
(Oxford Research A/S,2010)
©UNIVAS Anti-doping education
6

## Slide 8
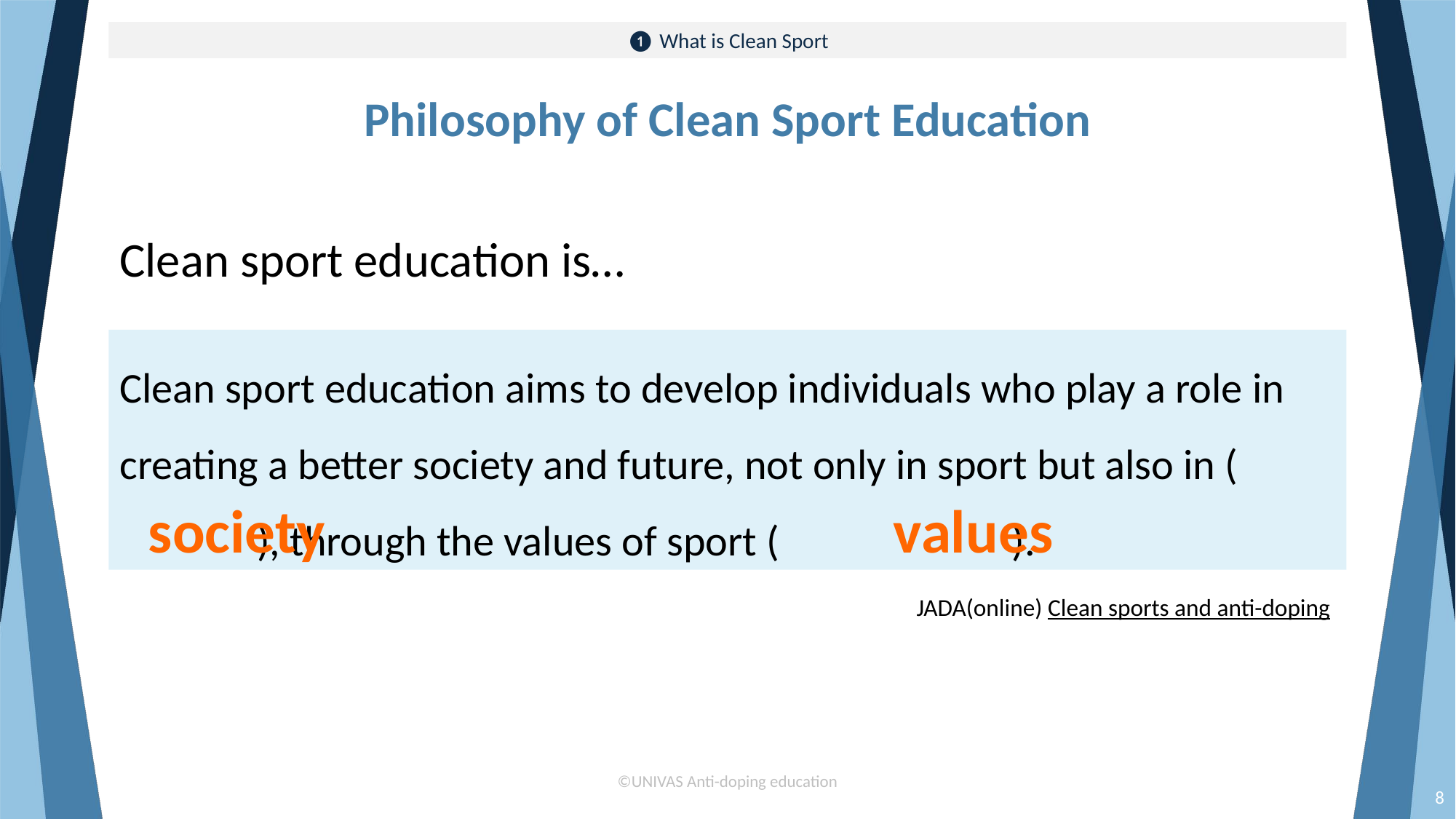

❶ What is Clean Sport
Philosophy of Clean Sport Education
Clean sport education is…
Clean sport education aims to develop individuals who play a role in creating a better society and future, not only in sport but also in ( 　   ), through the values of sport ( 　   ).
JADA(online) Clean sports and anti-doping
society
values
©UNIVAS Anti-doping education
7

## Slide 9
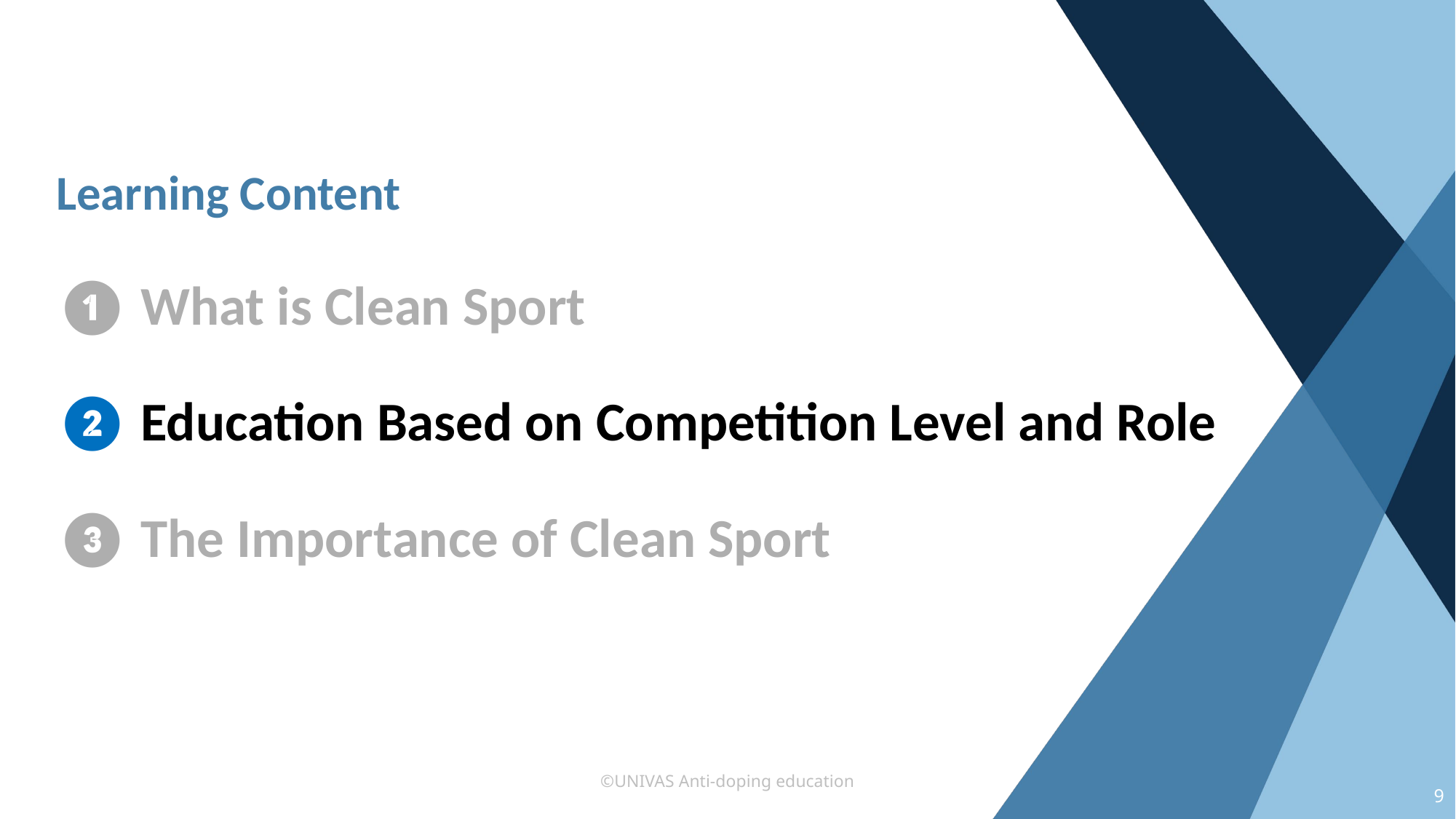

Learning Content
❶ What is Clean Sport
❷ Education Based on Competition Level and Role
❸ The Importance of Clean Sport
©UNIVAS Anti-doping education
8

## Slide 10
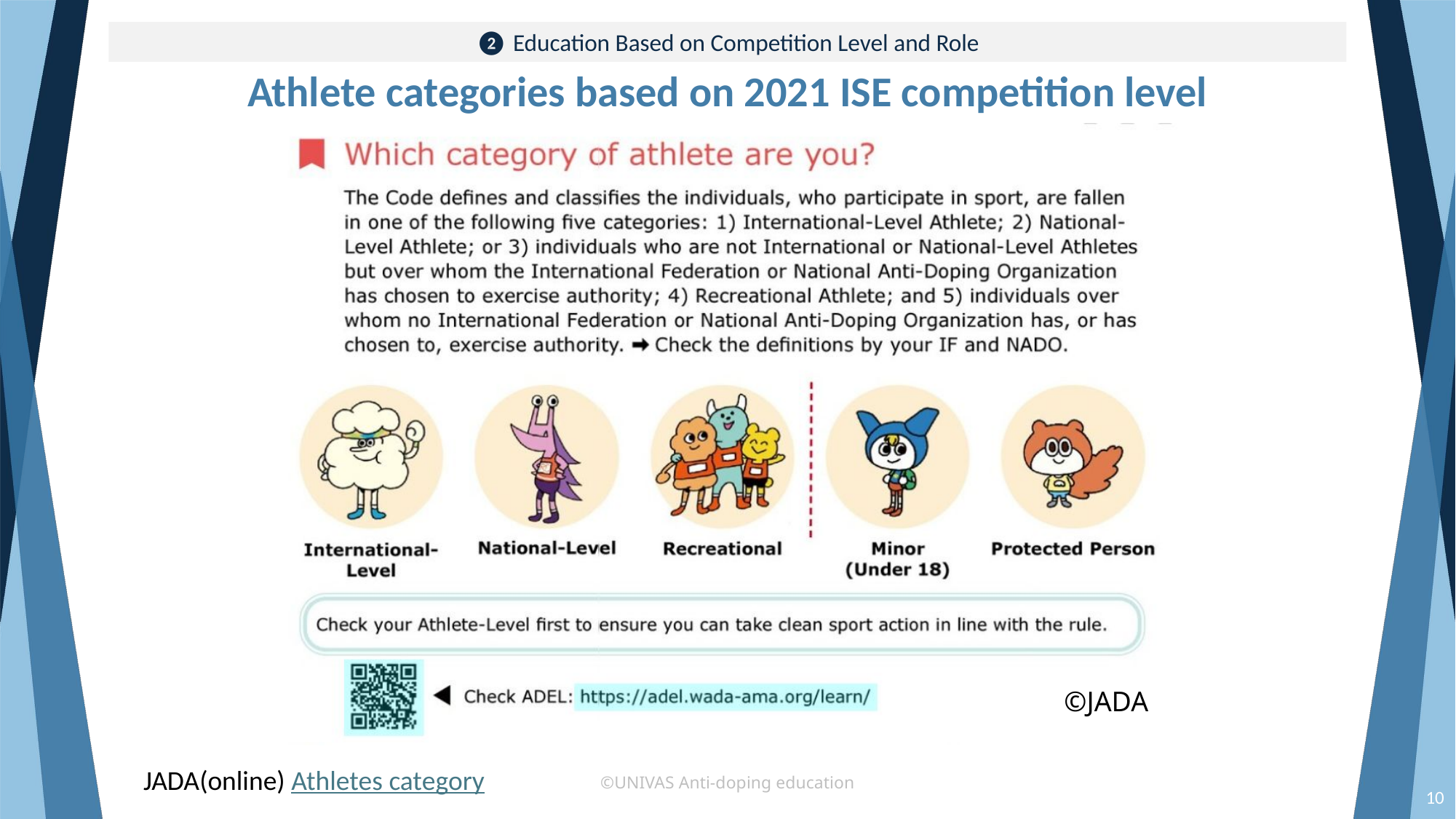

❷ Education Based on Competition Level and Role
Athlete categories based on 2021 ISE competition level
©JADA
JADA(online) Athletes category
©UNIVAS Anti-doping education
9

## Slide 11
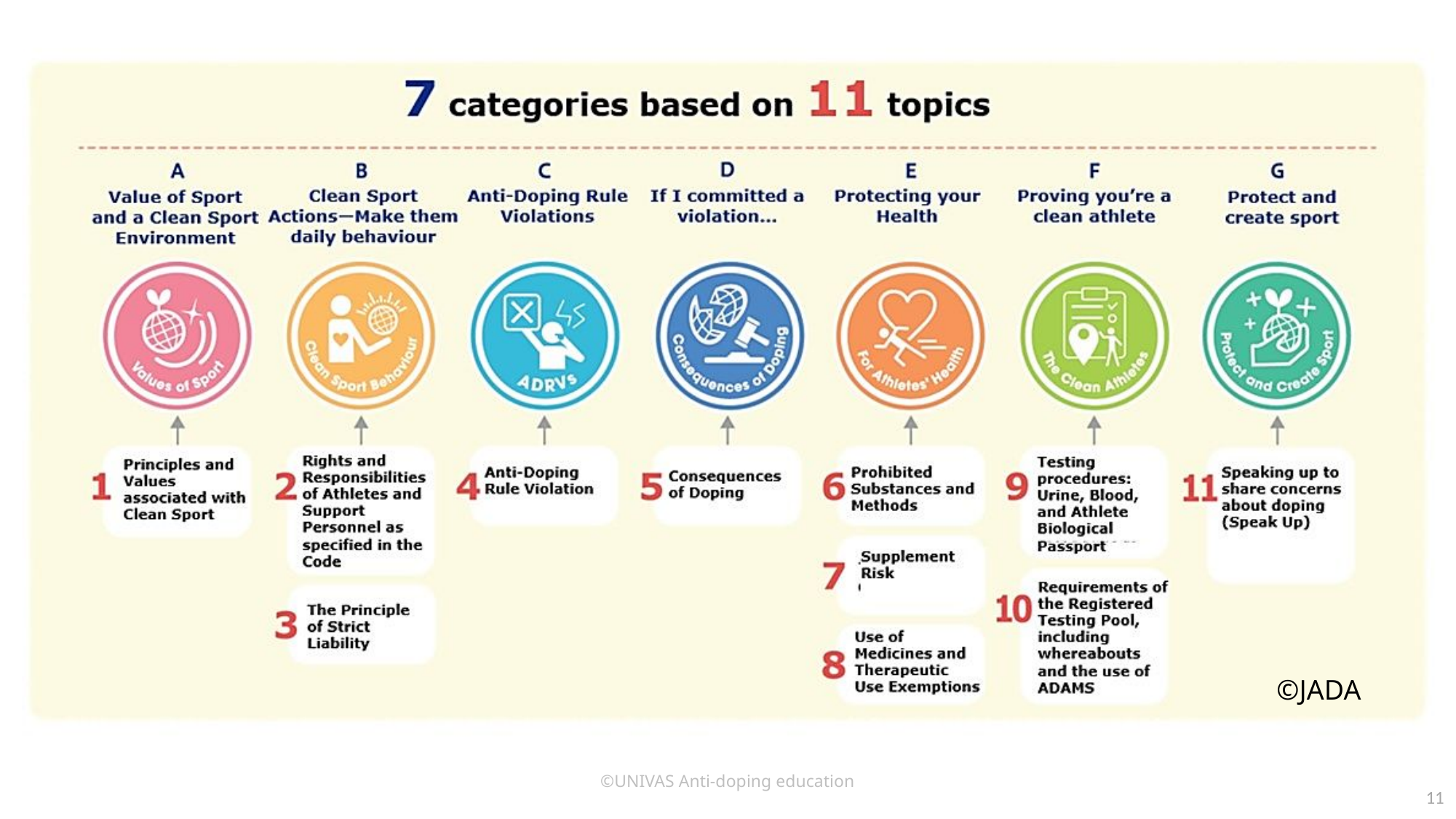

©JADA
©UNIVAS Anti-doping education
10

## Slide 12
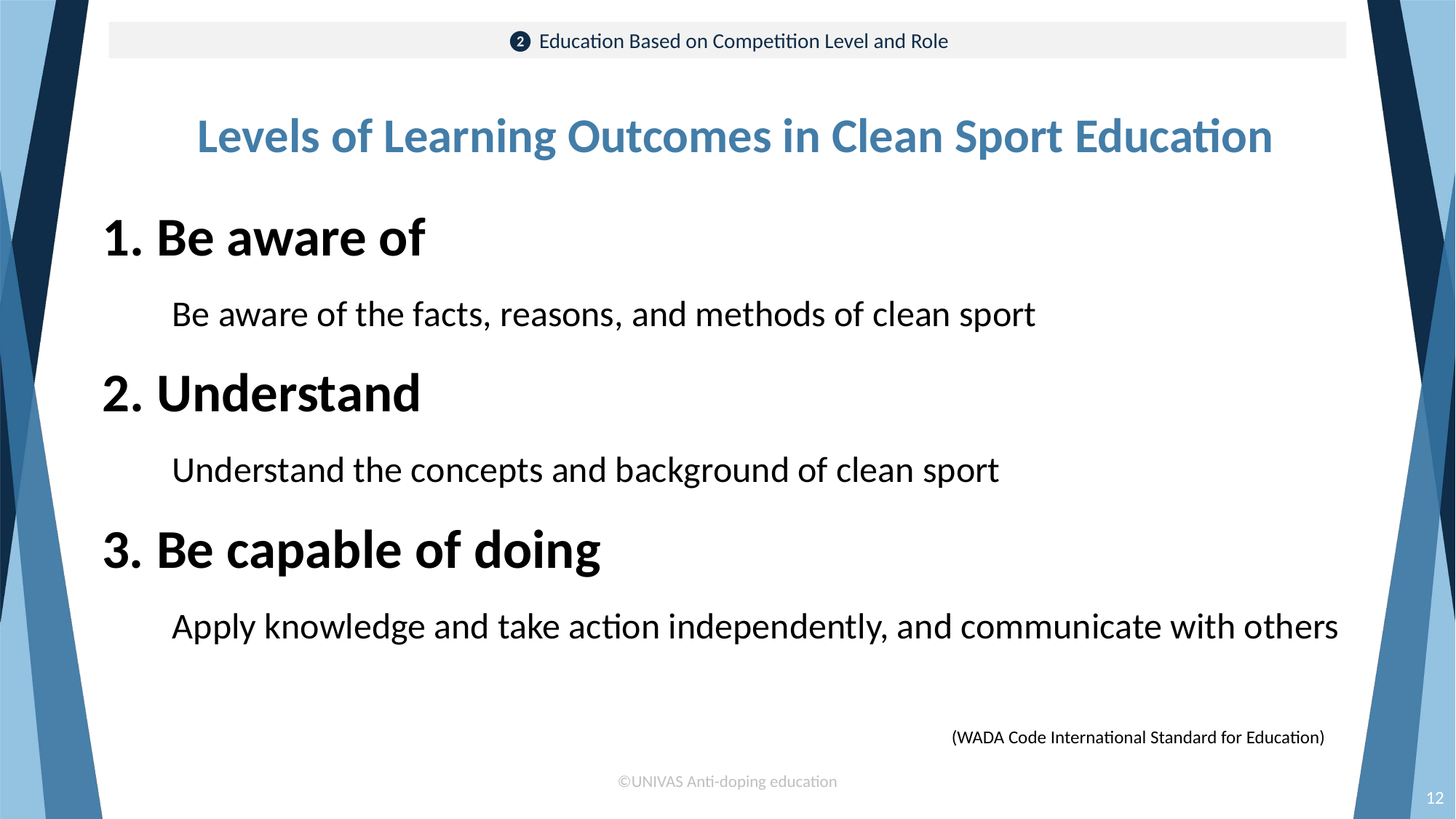

❷ Education Based on Competition Level and Role
Levels of Learning Outcomes in Clean Sport Education
1. Be aware of
Be aware of the facts, reasons, and methods of clean sport
2. Understand
Understand the concepts and background of clean sport
3. Be capable of doing
Apply knowledge and take action independently, and communicate with others
(WADA Code International Standard for Education)
©UNIVAS Anti-doping education
11

## Slide 13
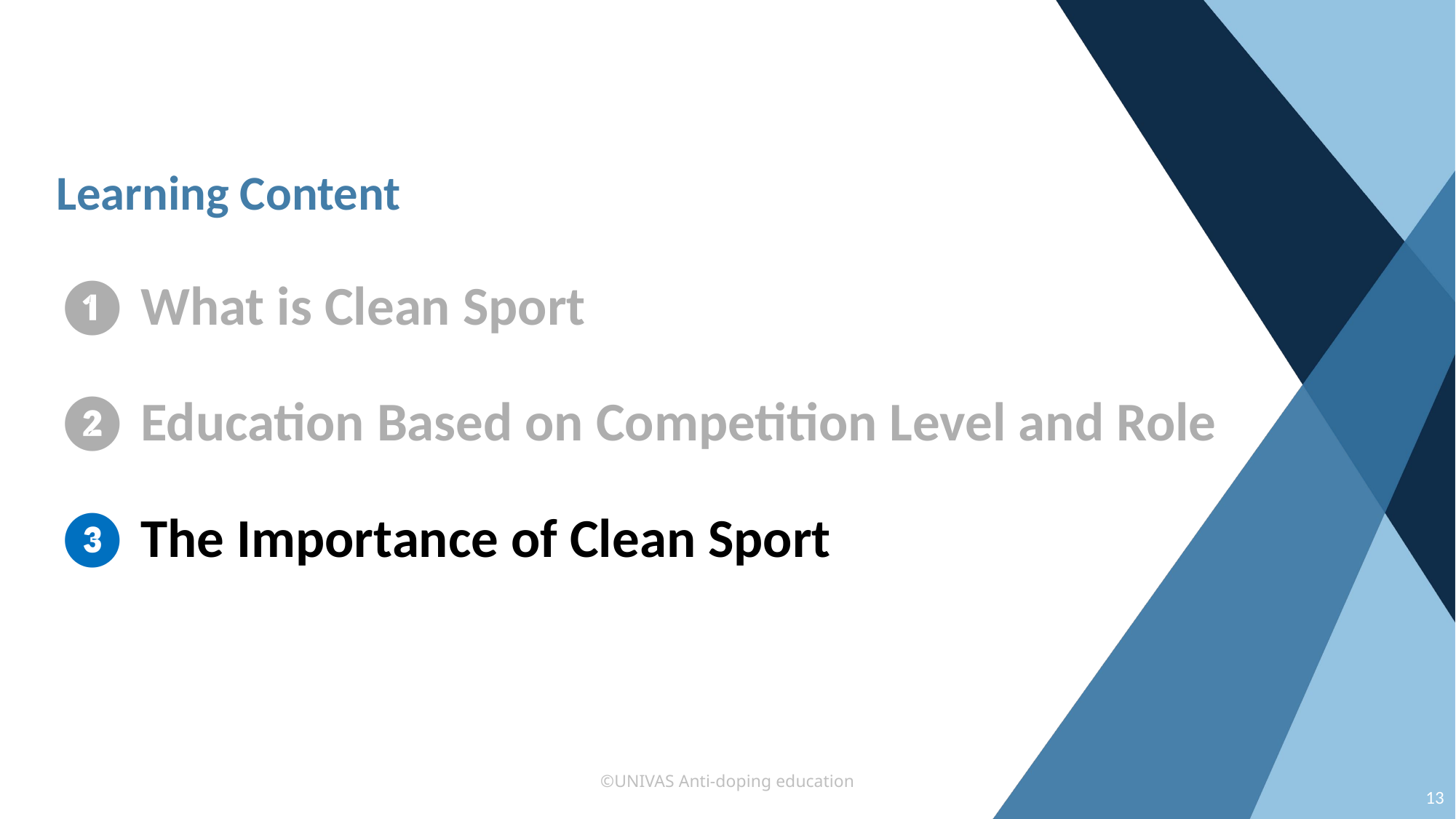

Learning Content
❶ What is Clean Sport
❷ Education Based on Competition Level and Role
❸ The Importance of Clean Sport
©UNIVAS Anti-doping education
12

## Slide 14
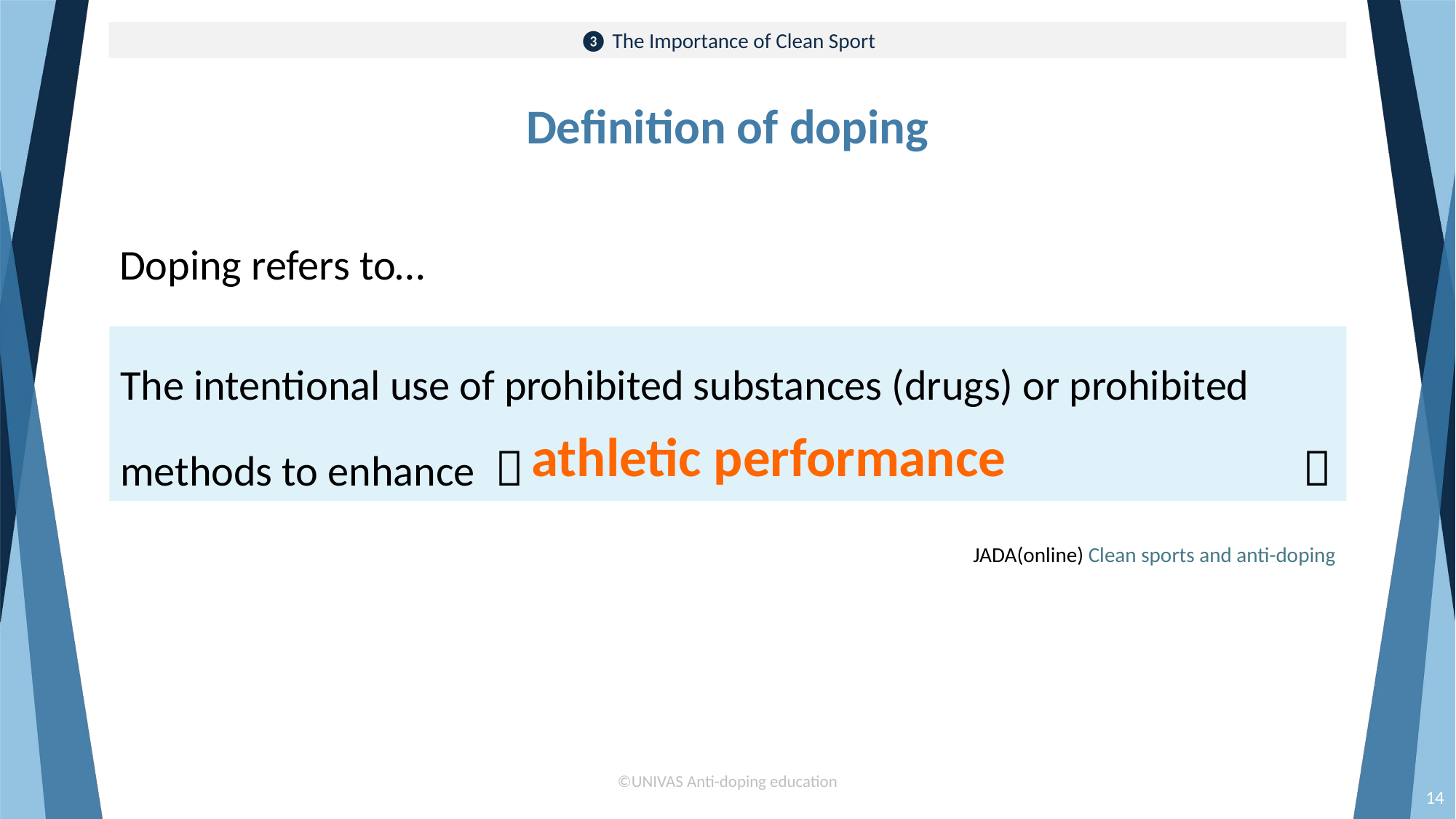

❸ The Importance of Clean Sport
Definition of doping
Doping refers to…
The intentional use of prohibited substances (drugs) or prohibited methods to enhance （ 　　　　　　　　 　　　　　　　）
athletic performance
JADA(online) Clean sports and anti-doping
©UNIVAS Anti-doping education
13

## Slide 15
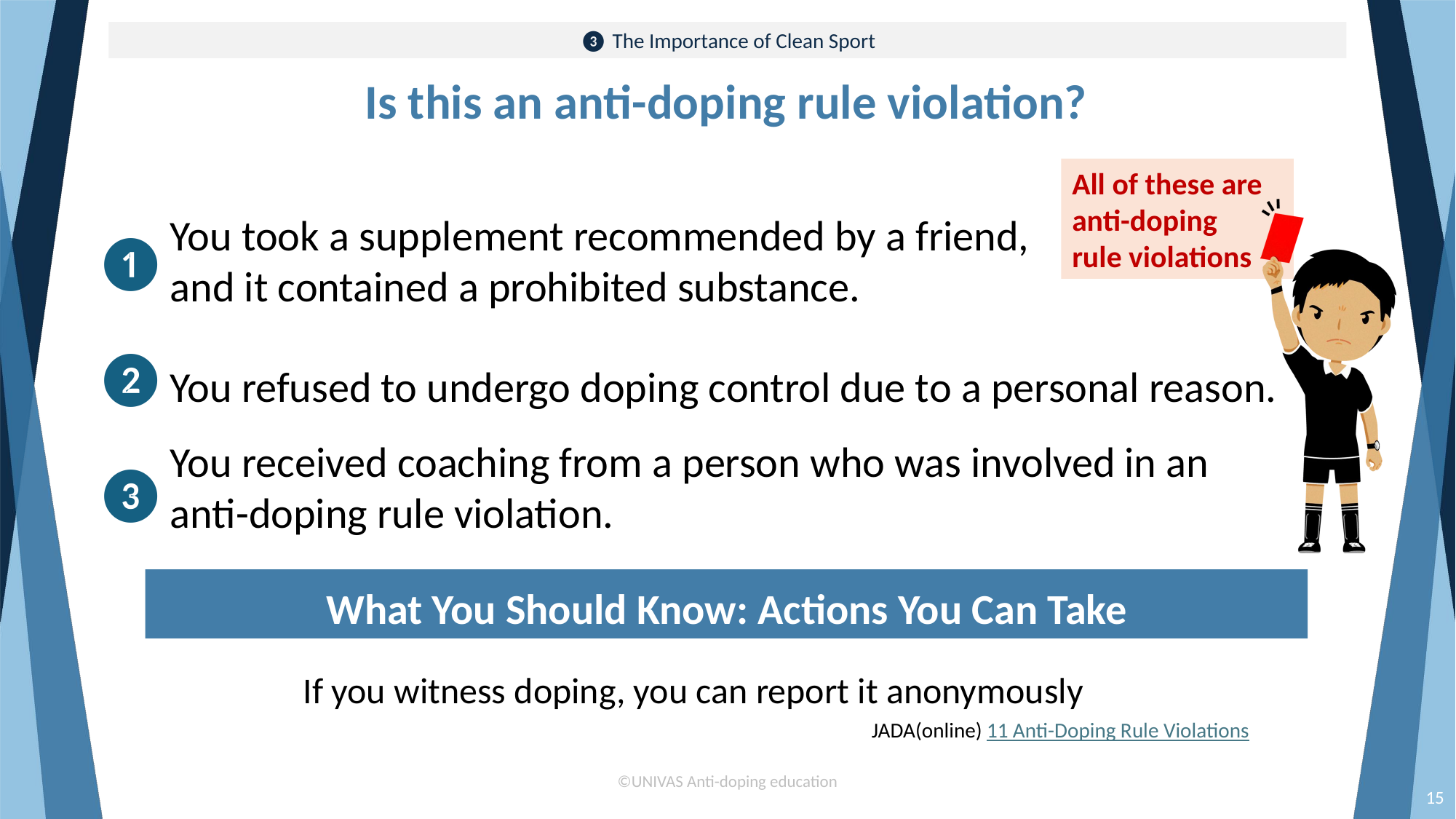

❸ The Importance of Clean Sport
Is this an anti-doping rule violation?
All of these are
anti-doping
rule violations
You took a supplement recommended by a friend,
and it contained a prohibited substance.
❶
You refused to undergo doping control due to a personal reason.
❷
You received coaching from a person who was involved in an anti-doping rule violation.
❸
What You Should Know: Actions You Can Take
If you witness doping, you can report it anonymously
JADA(online) 11 Anti-Doping Rule Violations
©UNIVAS Anti-doping education
14

## Slide 16
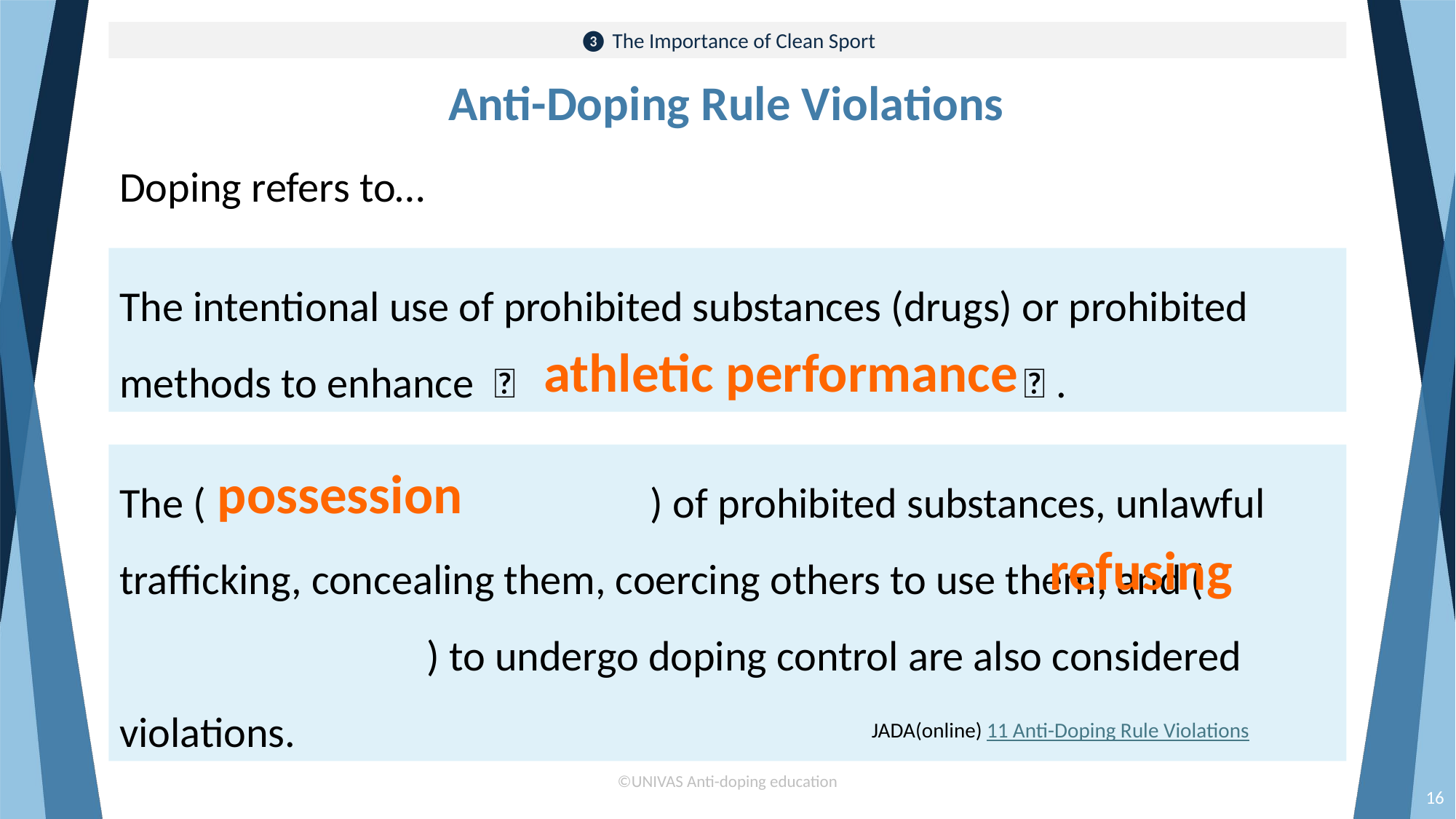

❸ The Importance of Clean Sport
Anti-Doping Rule Violations
Doping refers to…
The intentional use of prohibited substances (drugs) or prohibited methods to enhance （　　　　　　　　　　　　）.
athletic performance
The (　　　　　　　　　　) of prohibited substances, unlawful trafficking, concealing them, coercing others to use them, and (　　　　　　　　　) to undergo doping control are also considered violations.
possession
refusing
JADA(online) 11 Anti-Doping Rule Violations
©UNIVAS Anti-doping education
15

## Slide 17
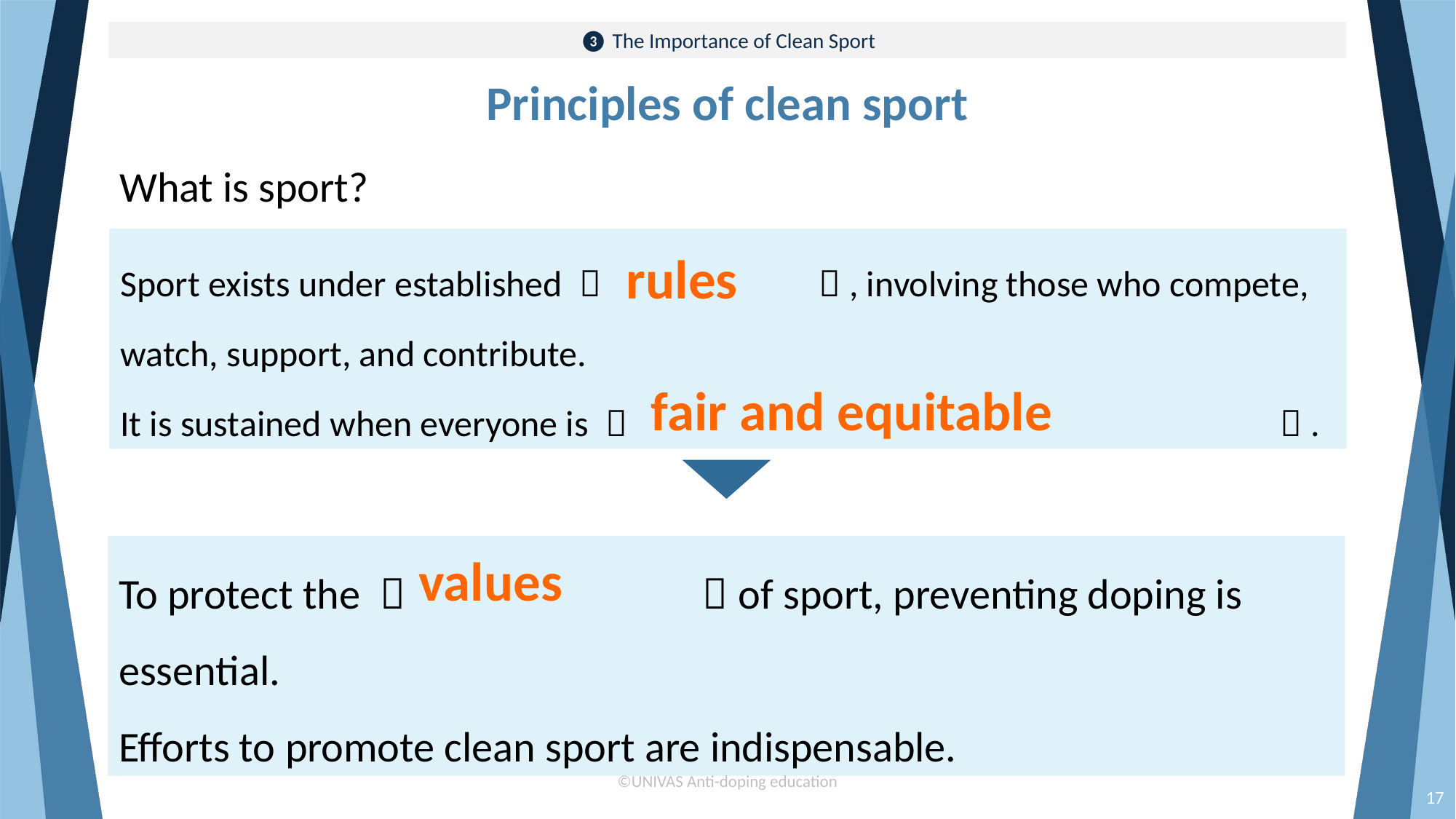

❸ The Importance of Clean Sport
Principles of clean sport
What is sport?
Sport exists under established （ ）, involving those who compete, watch, support, and contribute.
It is sustained when everyone is （ 　　　　　　　　　　　　　 ）.
rules
fair and equitable
To protect the （　　　　　　　）of sport, preventing doping is essential.
Efforts to promote clean sport are indispensable.
values
©UNIVAS Anti-doping education
16

## Slide 18
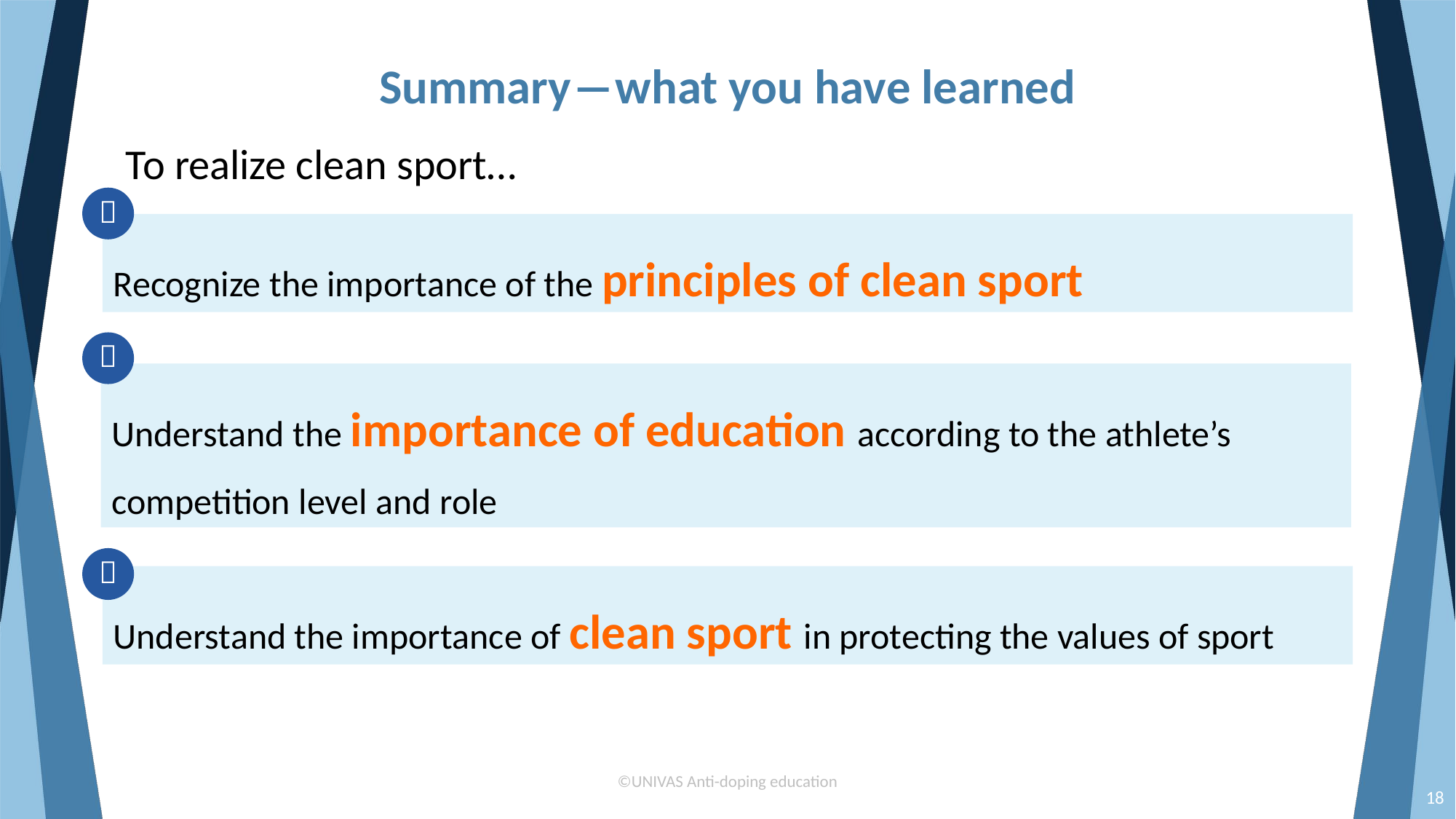

Summary―what you have learned
To realize clean sport…
１
Recognize the importance of the principles of clean sport
２
Understand the importance of education according to the athlete’s competition level and role
３
Understand the importance of clean sport in protecting the values of sport
©UNIVAS Anti-doping education
17

## Slide 19
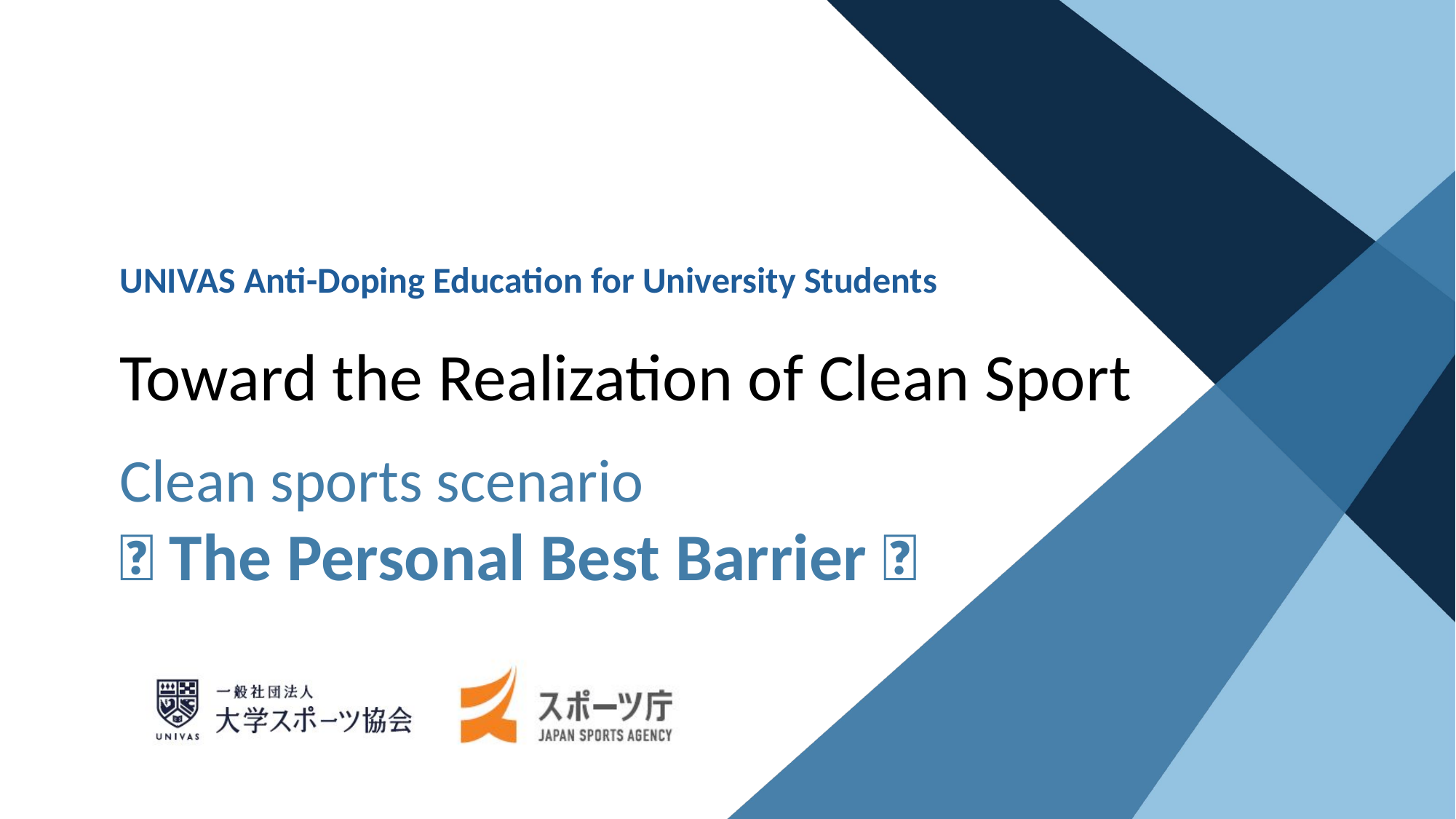

UNIVAS Anti-Doping Education for University Students
Toward the Realization of Clean Sport
Clean sports scenario
ーThe Personal Best Barrierー

## Slide 20
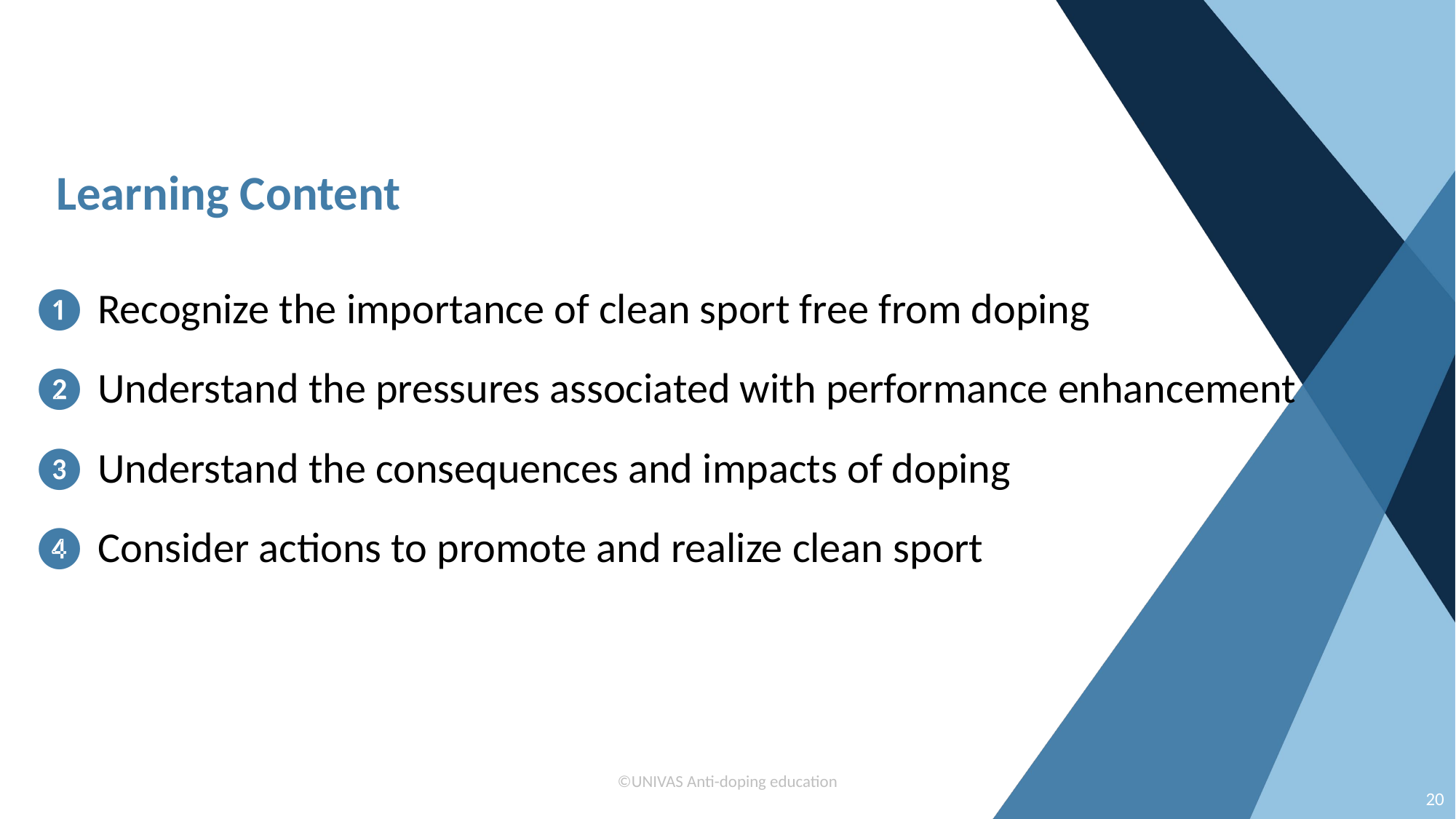

Learning Content
❶ Recognize the importance of clean sport free from doping
❷ Understand the pressures associated with performance enhancement
❸ Understand the consequences and impacts of doping
❹ Consider actions to promote and realize clean sport
©UNIVAS Anti-doping education
19

## Slide 21
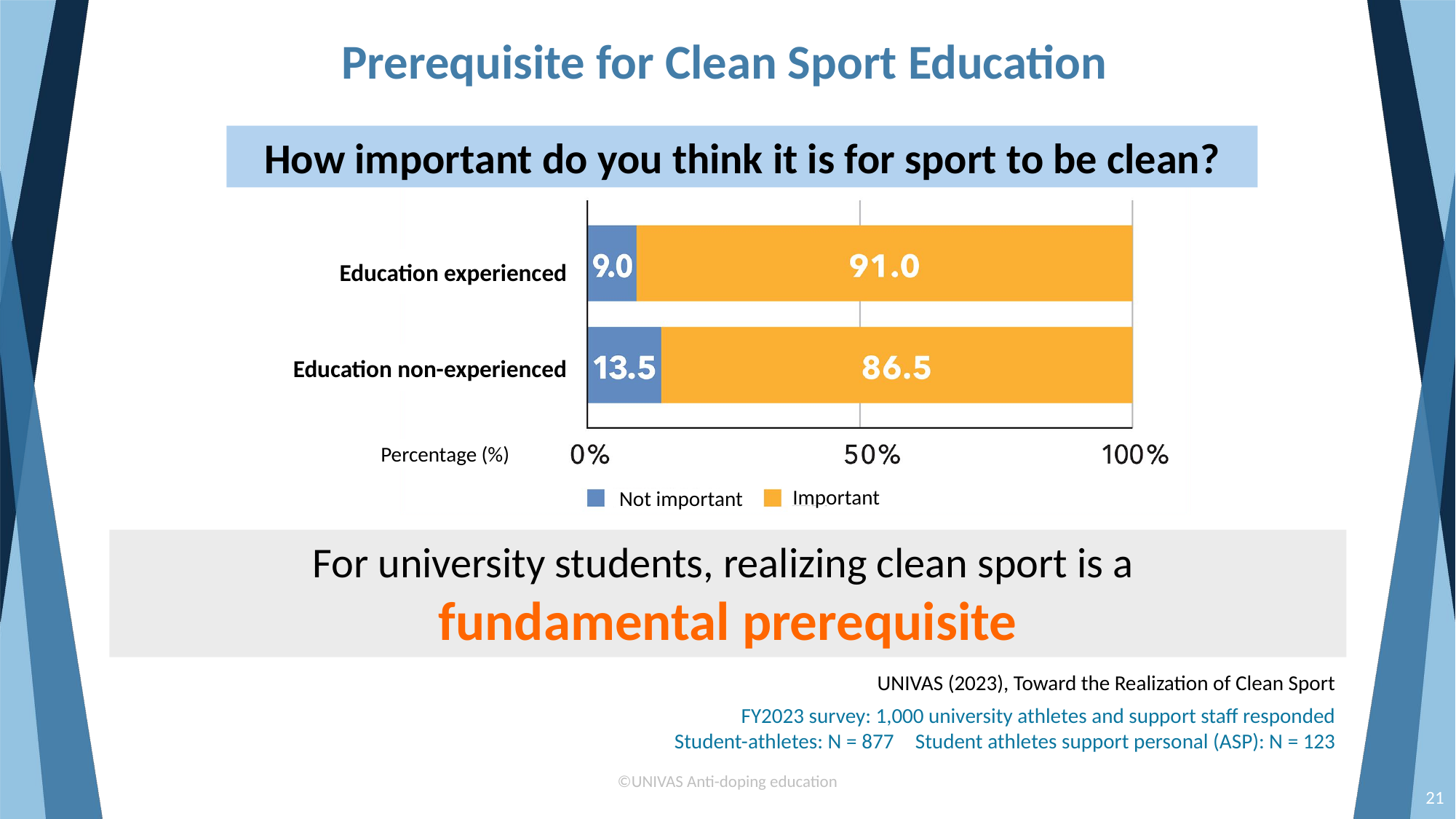

Prerequisite for Clean Sport Education
How important do you think it is for sport to be clean?
Education experienced
Education non-experienced
Percentage (%)
Important
Not important
For university students, realizing clean sport is a
fundamental prerequisite
UNIVAS (2023), Toward the Realization of Clean Sport
FY2023 survey: 1,000 university athletes and support staff responded
Student-athletes: N = 877 Student athletes support personal (ASP): N = 123
©UNIVAS Anti-doping education
20

## Slide 22
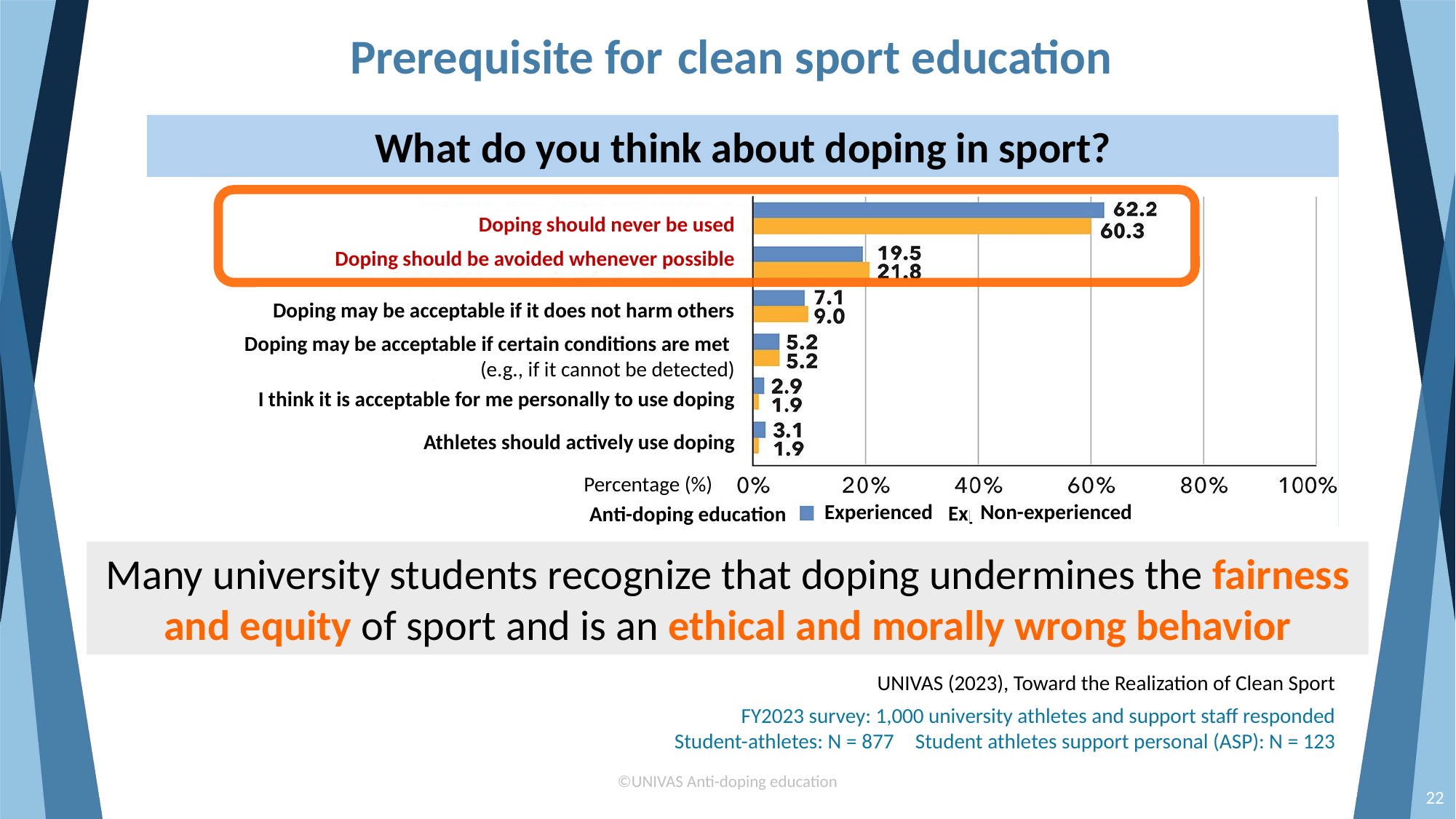

Prerequisite for 	clean sport education
What do you think about doping in sport?
Doping should never be used
Doping should be avoided whenever possible
Doping may be acceptable if it does not harm others
Doping may be acceptable if certain conditions are met
(e.g., if it cannot be detected)
I think it is acceptable for me personally to use doping
Athletes should actively use doping
Percentage (%)
Experienced
Non-experienced
Anti-doping education
Experienced
Many university students recognize that doping undermines the fairness and equity of sport and is an ethical and morally wrong behavior
UNIVAS (2023), Toward the Realization of Clean Sport
FY2023 survey: 1,000 university athletes and support staff responded
Student-athletes: N = 877 Student athletes support personal (ASP): N = 123
©UNIVAS Anti-doping education
21

## Slide 23
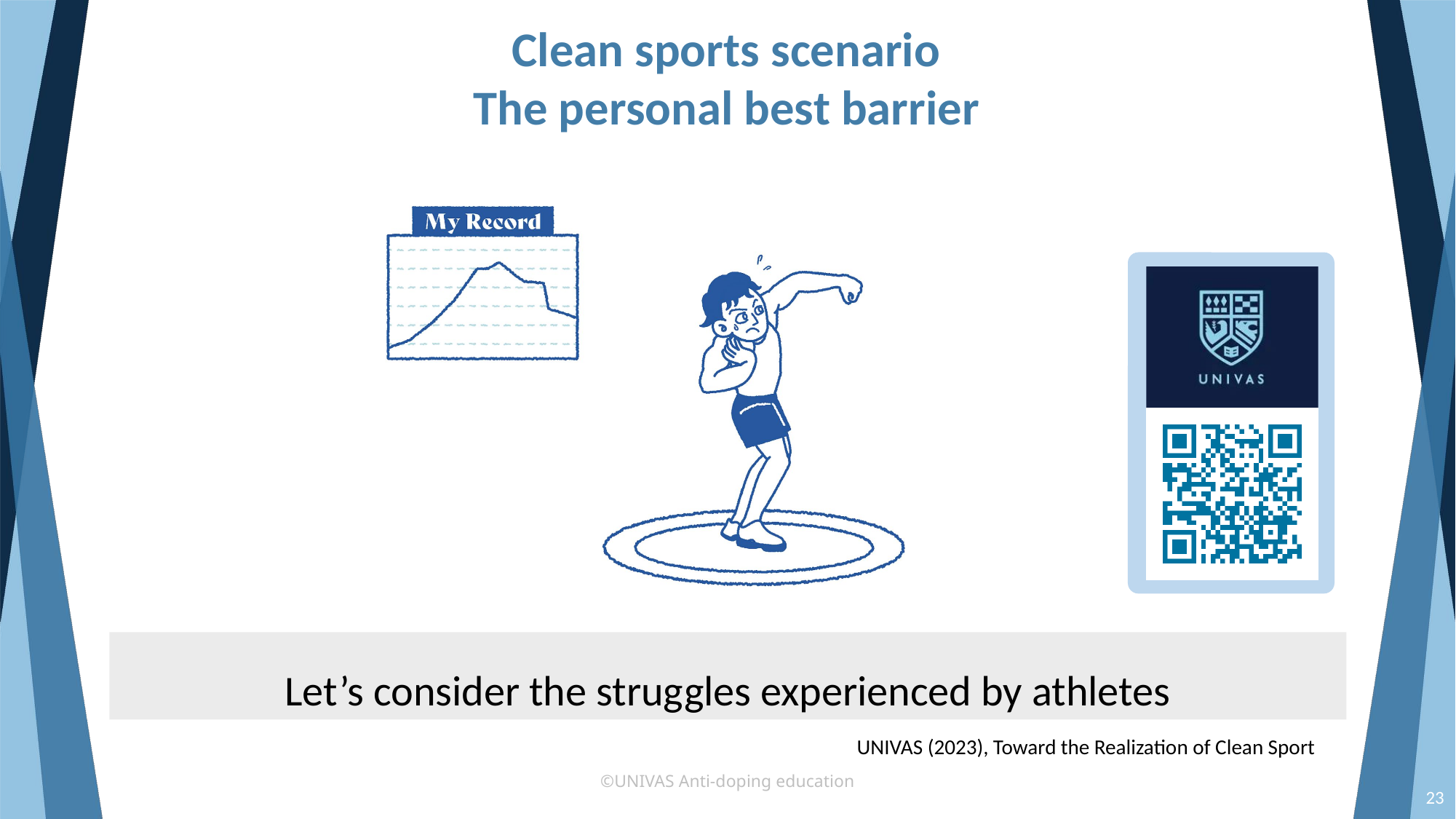

Clean sports scenario
The personal best barrier
Let’s consider the struggles experienced by athletes
UNIVAS (2023), Toward the Realization of Clean Sport
©UNIVAS Anti-doping education
22

## Slide 24
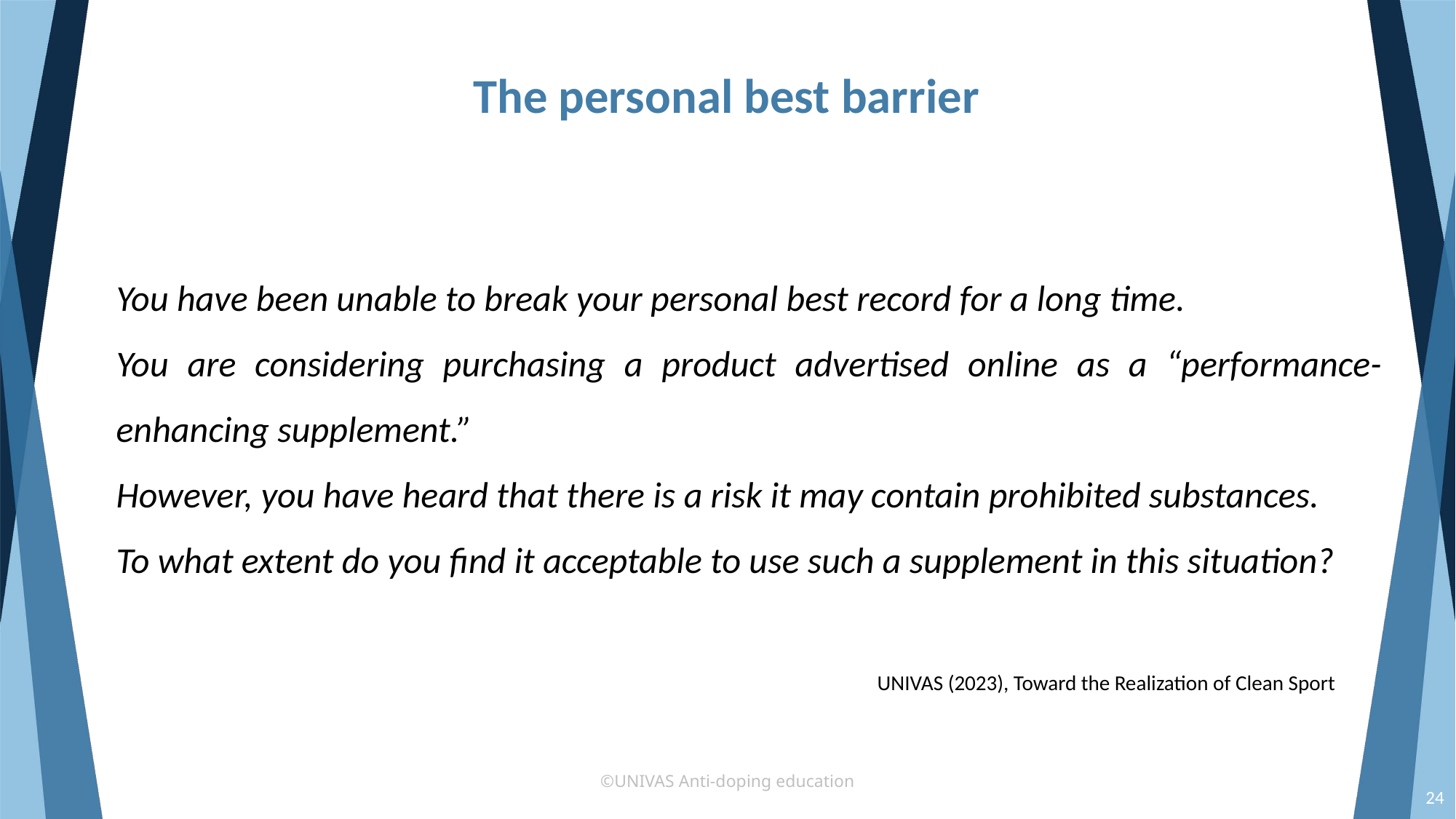

The personal best barrier
You have been unable to break your personal best record for a long time.
You are considering purchasing a product advertised online as a “performance-enhancing supplement.”
However, you have heard that there is a risk it may contain prohibited substances.
To what extent do you find it acceptable to use such a supplement in this situation?
23
UNIVAS (2023), Toward the Realization of Clean Sport
©UNIVAS Anti-doping education
23

## Slide 25
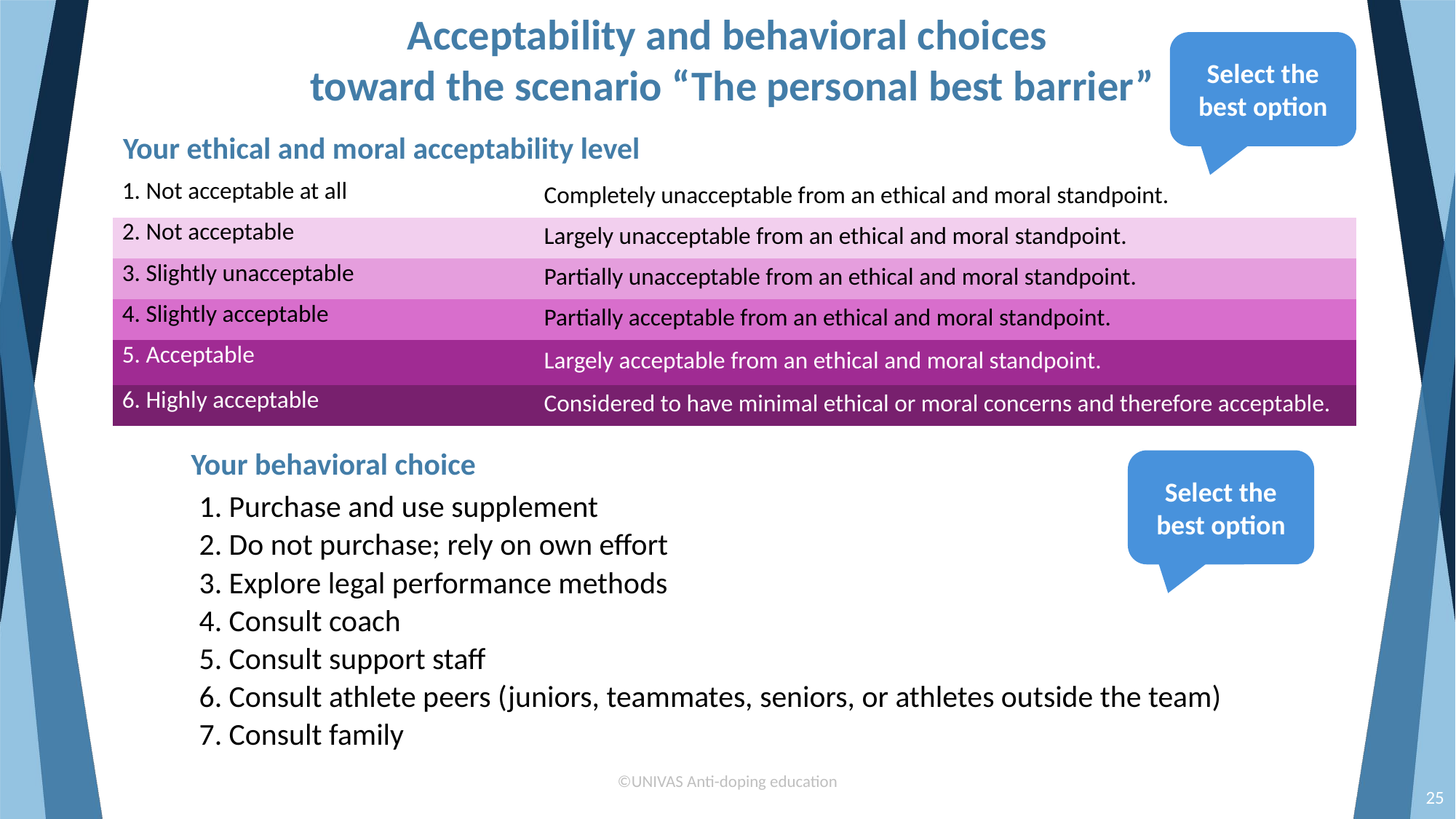

Acceptability and behavioral choices
toward the scenario “The personal best barrier”
Select the best option
Your ethical and moral acceptability level
| 1. Not acceptable at all | Completely unacceptable from an ethical and moral standpoint. |
| --- | --- |
| 2. Not acceptable | Largely unacceptable from an ethical and moral standpoint. |
| 3. Slightly unacceptable | Partially unacceptable from an ethical and moral standpoint. |
| 4. Slightly acceptable | Partially acceptable from an ethical and moral standpoint. |
| 5. Acceptable | Largely acceptable from an ethical and moral standpoint. |
| 6. Highly acceptable | Considered to have minimal ethical or moral concerns and therefore acceptable. |
Your behavioral choice
Select the best option
| 1. Purchase and use supplement |
| --- |
| 2. Do not purchase; rely on own effort |
| 3. Explore legal performance methods |
| 4. Consult coach |
| 5. Consult support staff |
| 6. Consult athlete peers (juniors, teammates, seniors, or athletes outside the team) |
| 7. Consult family |
©UNIVAS Anti-doping education
24

## Slide 26
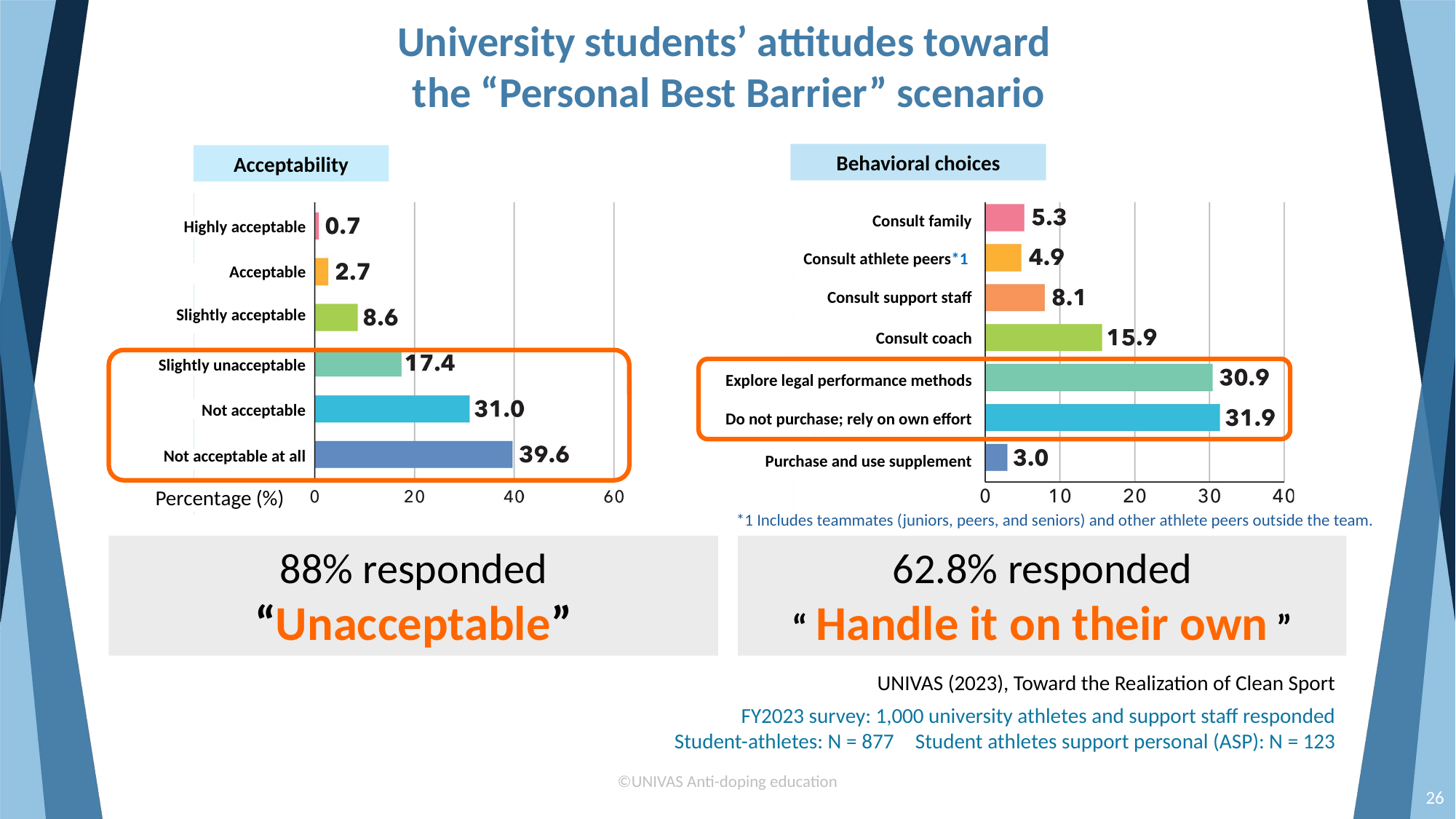

University students’ attitudes toward
the “Personal Best Barrier” scenario
Behavioral choices
Consult family
Consult athlete peers*1
Consult support staff
Consult coach
Explore legal performance methods
Do not purchase; rely on own effort
Purchase and use supplement
*1 Includes teammates (juniors, peers, and seniors) and other athlete peers outside the team.
Acceptability
Highly acceptable
Acceptable
Slightly acceptable
Slightly unacceptable
Not acceptable
Not acceptable at all
Percentage (%)
88% responded “Unacceptable”
62.8% responded
“ Handle it on their own ”
UNIVAS (2023), Toward the Realization of Clean Sport
FY2023 survey: 1,000 university athletes and support staff responded
Student-athletes: N = 877 Student athletes support personal (ASP): N = 123
©UNIVAS Anti-doping education
25

## Slide 27
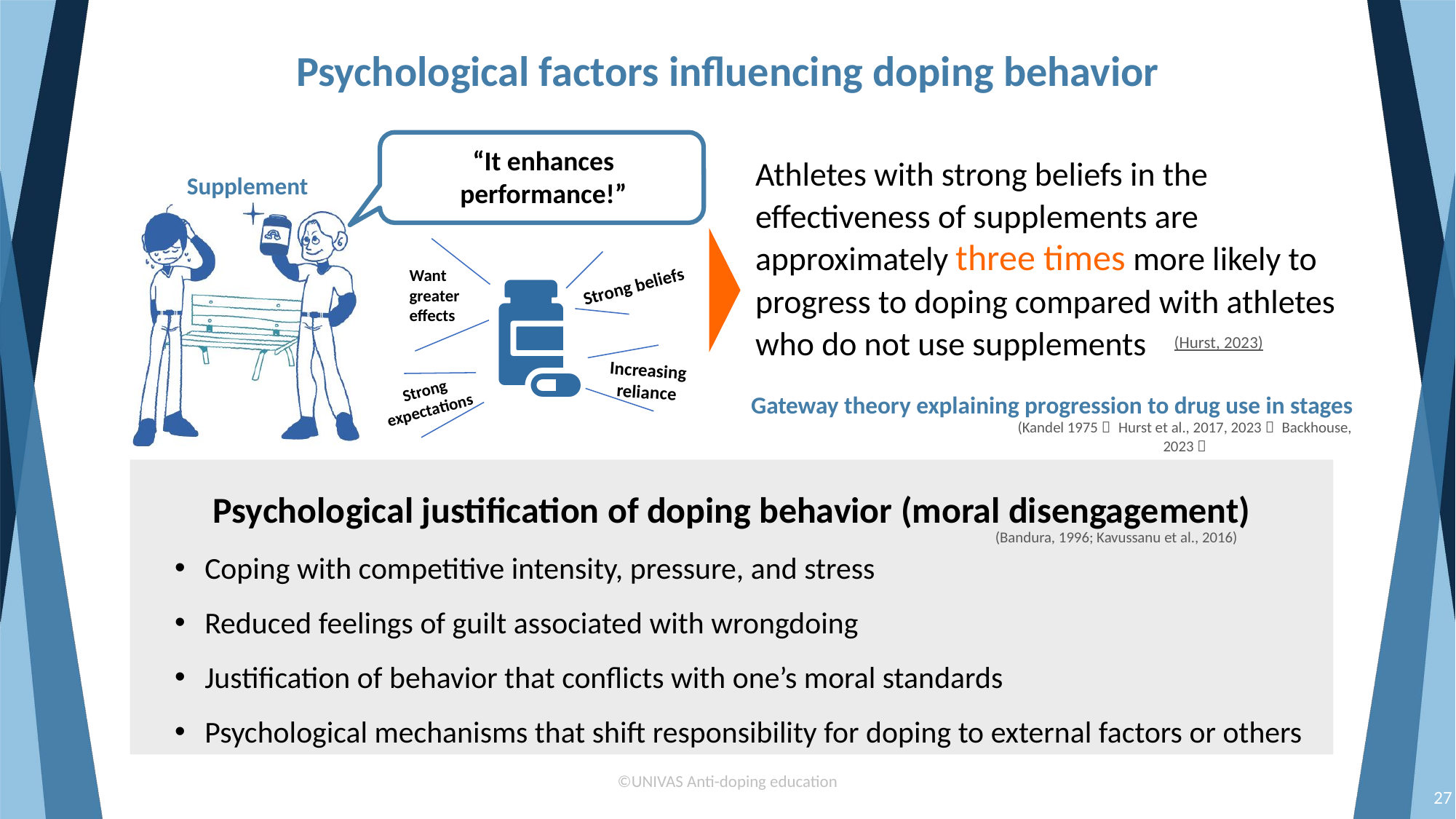

Psychological factors influencing doping behavior
Supplement
“It enhances performance!”
Athletes with strong beliefs in the effectiveness of supplements are approximately three times more likely to progress to doping compared with athletes who do not use supplements
Gateway theory explaining progression to drug use in stages
(Kandel 1975； Hurst et al., 2017, 2023： Backhouse, 2023）
(Hurst, 2023)
Strong beliefs
Want greater effects
Increasing reliance
Strong expectations
Psychological justification of doping behavior (moral disengagement)
Coping with competitive intensity, pressure, and stress
Reduced feelings of guilt associated with wrongdoing
Justification of behavior that conflicts with one’s moral standards
Psychological mechanisms that shift responsibility for doping to external factors or others
(Bandura, 1996; Kavussanu et al., 2016)
©UNIVAS Anti-doping education
26

## Slide 28
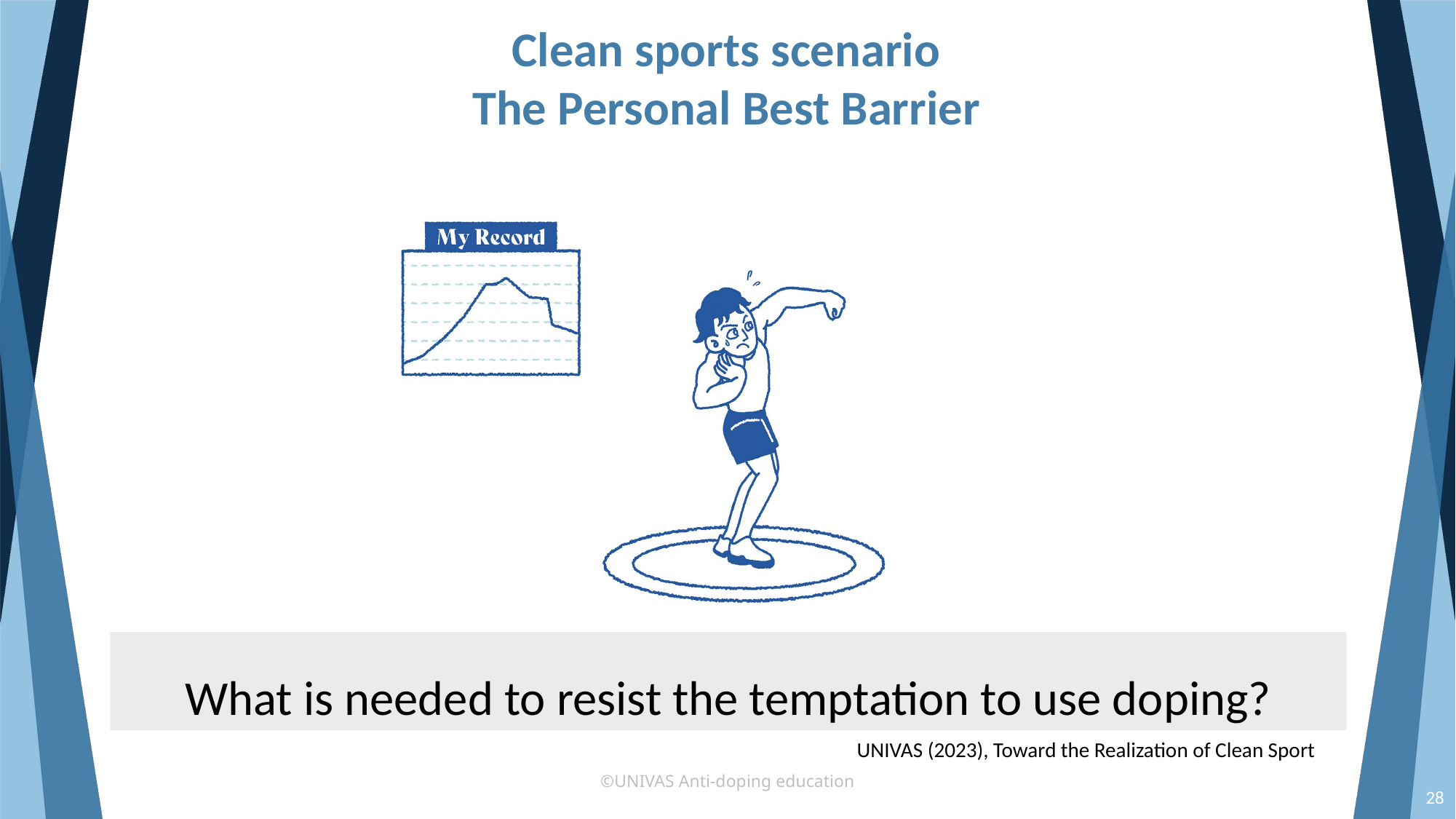

Clean sports scenario
The Personal Best Barrier
What is needed to resist the temptation to use doping?
UNIVAS (2023), Toward the Realization of Clean Sport
©UNIVAS Anti-doping education
27

## Slide 29
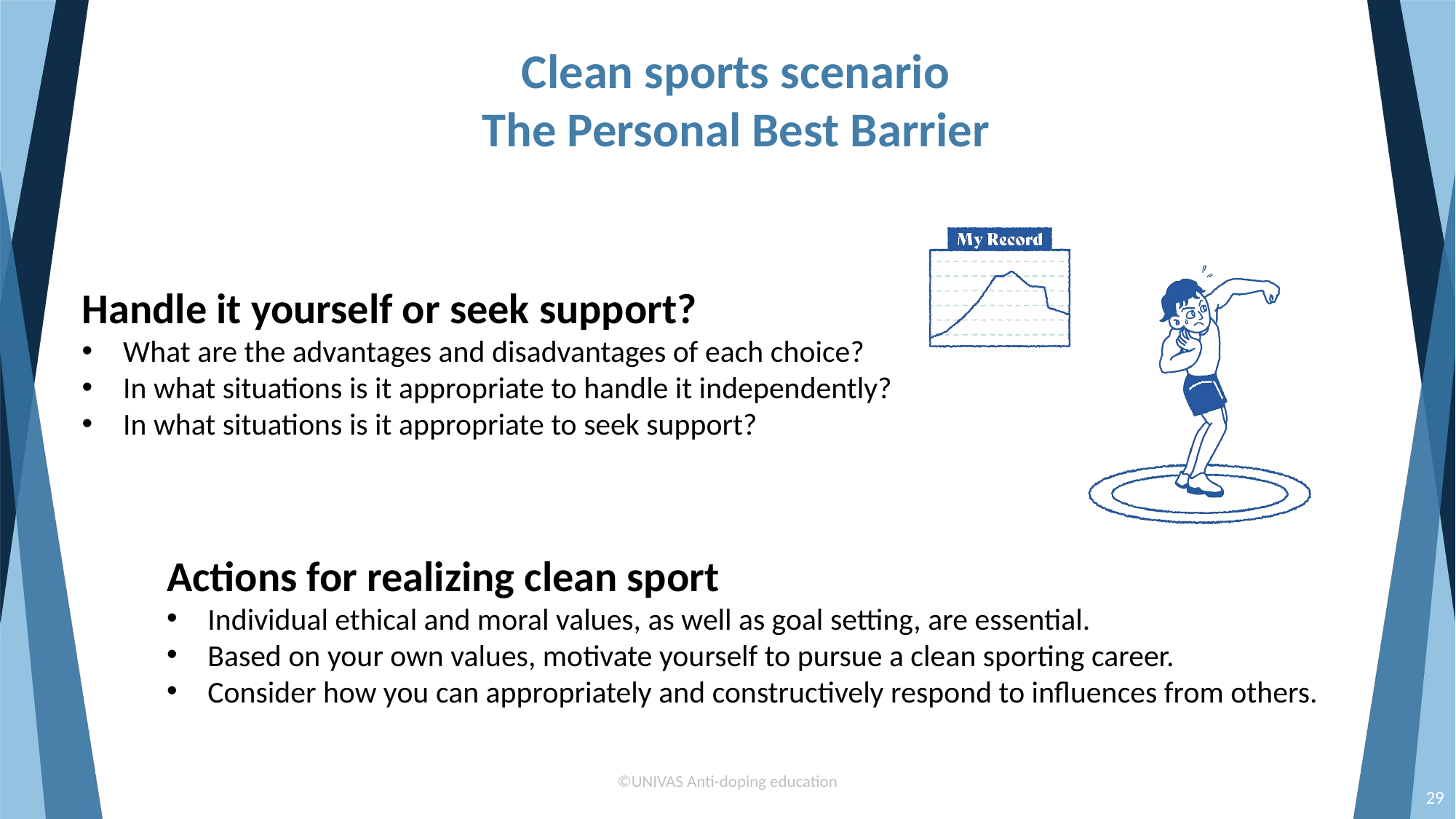

Clean sports scenario
The Personal Best Barrier
Handle it yourself or seek support?
What are the advantages and disadvantages of each choice?
In what situations is it appropriate to handle it independently?
In what situations is it appropriate to seek support?
Actions for realizing clean sport
Individual ethical and moral values, as well as goal setting, are essential.
Based on your own values, motivate yourself to pursue a clean sporting career.
Consider how you can appropriately and constructively respond to influences from others.
©UNIVAS Anti-doping education
28

## Slide 30
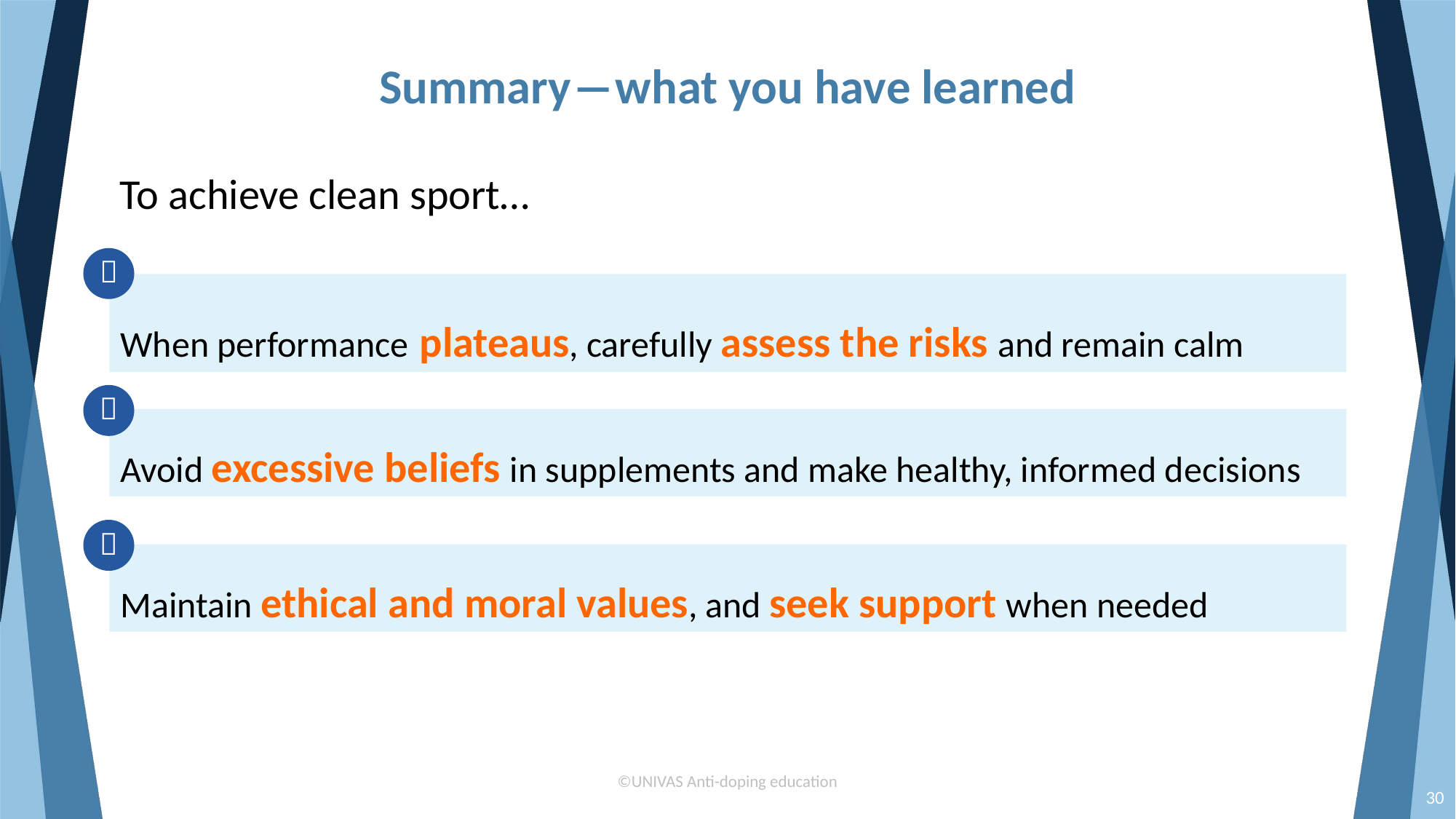

Summary―what you have learned
To achieve clean sport…
１
When performance plateaus, carefully assess the risks and remain calm
２
Avoid excessive beliefs in supplements and make healthy, informed decisions
３
Maintain ethical and moral values, and seek support when needed
©UNIVAS Anti-doping education
29

## Slide 31
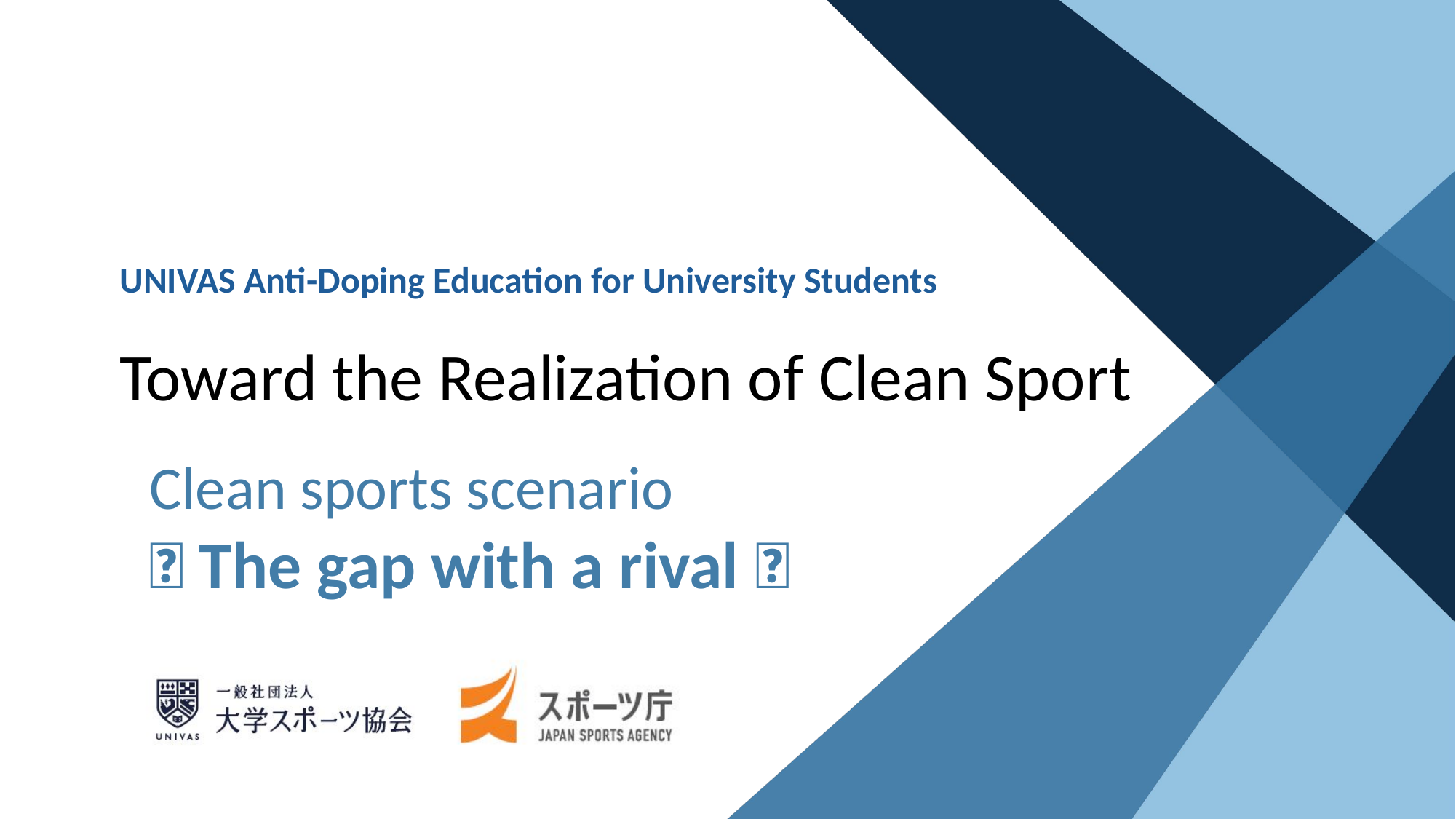

UNIVAS Anti-Doping Education for University Students
Toward the Realization of Clean Sport
Clean sports scenario
ーThe gap with a rivalー

## Slide 32
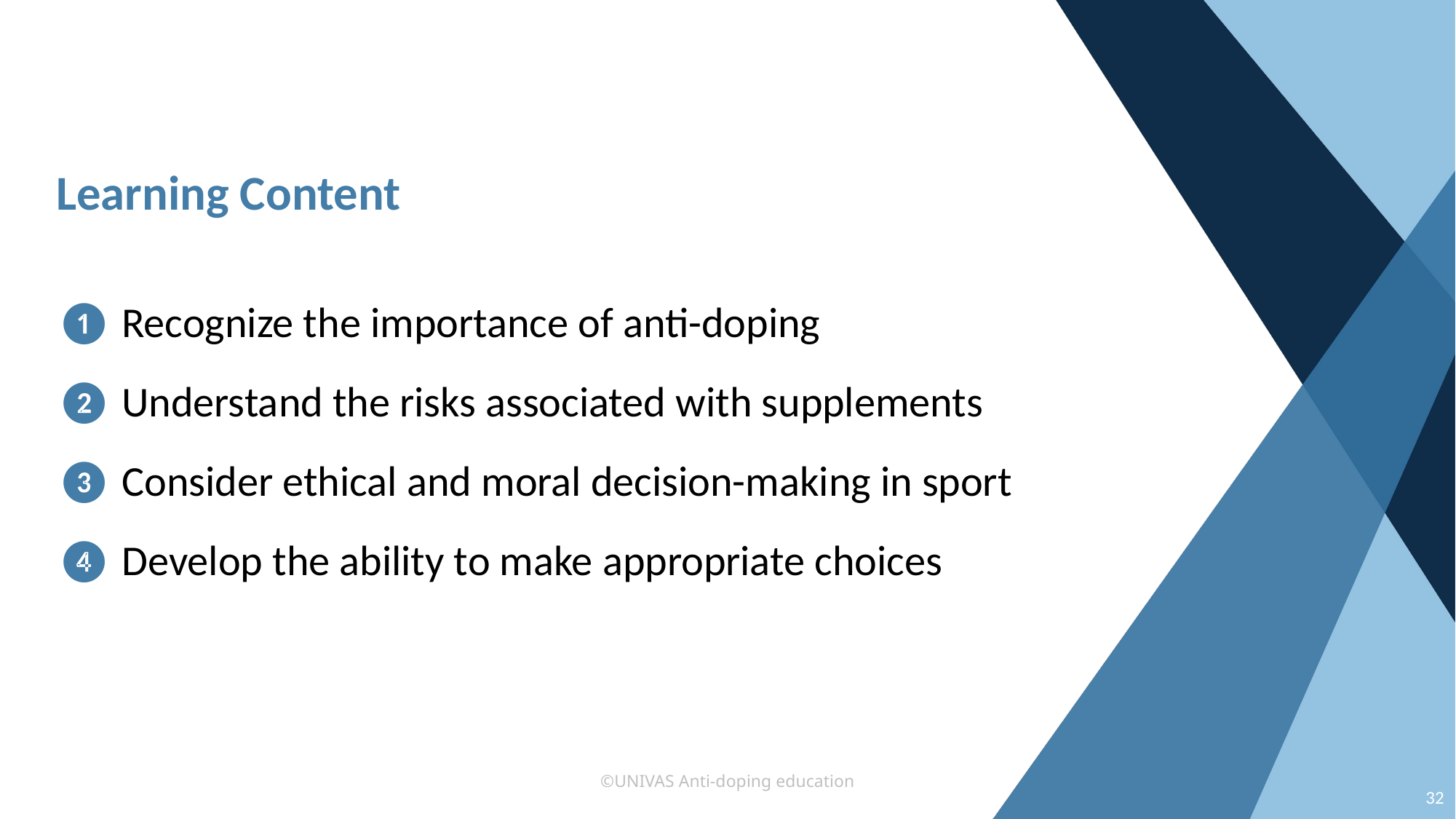

Learning Content
❶ Recognize the importance of anti-doping
❷ Understand the risks associated with supplements
❸ Consider ethical and moral decision-making in sport
❹ Develop the ability to make appropriate choices
©UNIVAS Anti-doping education
31

## Slide 33
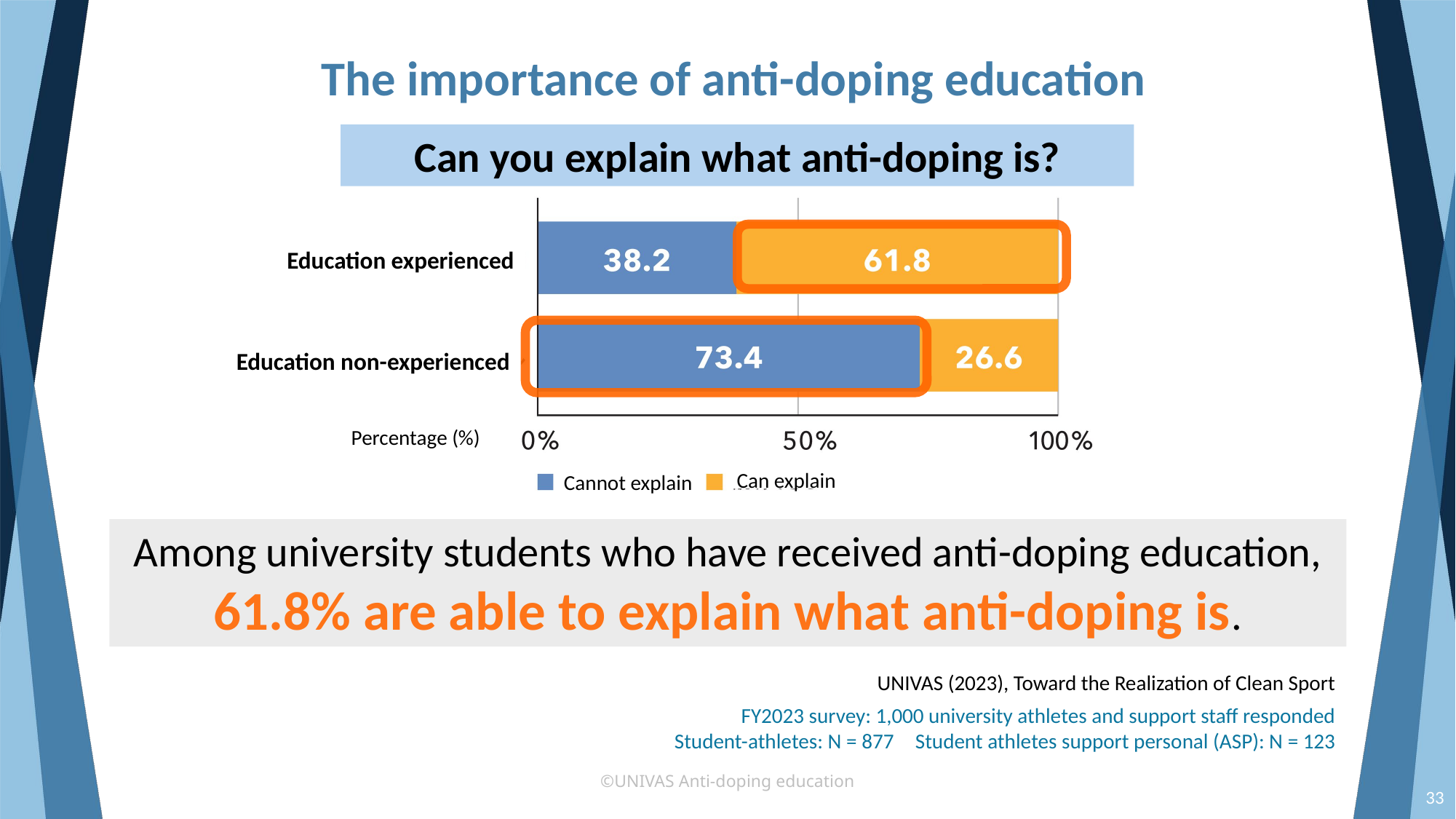

The importance of anti-doping education
Can you explain what anti-doping is?
Education experienced
Education non-experienced
Percentage (%)
Can explain
Cannot explain
Among university students who have received anti-doping education, 61.8% are able to explain what anti-doping is.
UNIVAS (2023), Toward the Realization of Clean Sport
FY2023 survey: 1,000 university athletes and support staff responded
Student-athletes: N = 877 Student athletes support personal (ASP): N = 123
©UNIVAS Anti-doping education
32

## Slide 34
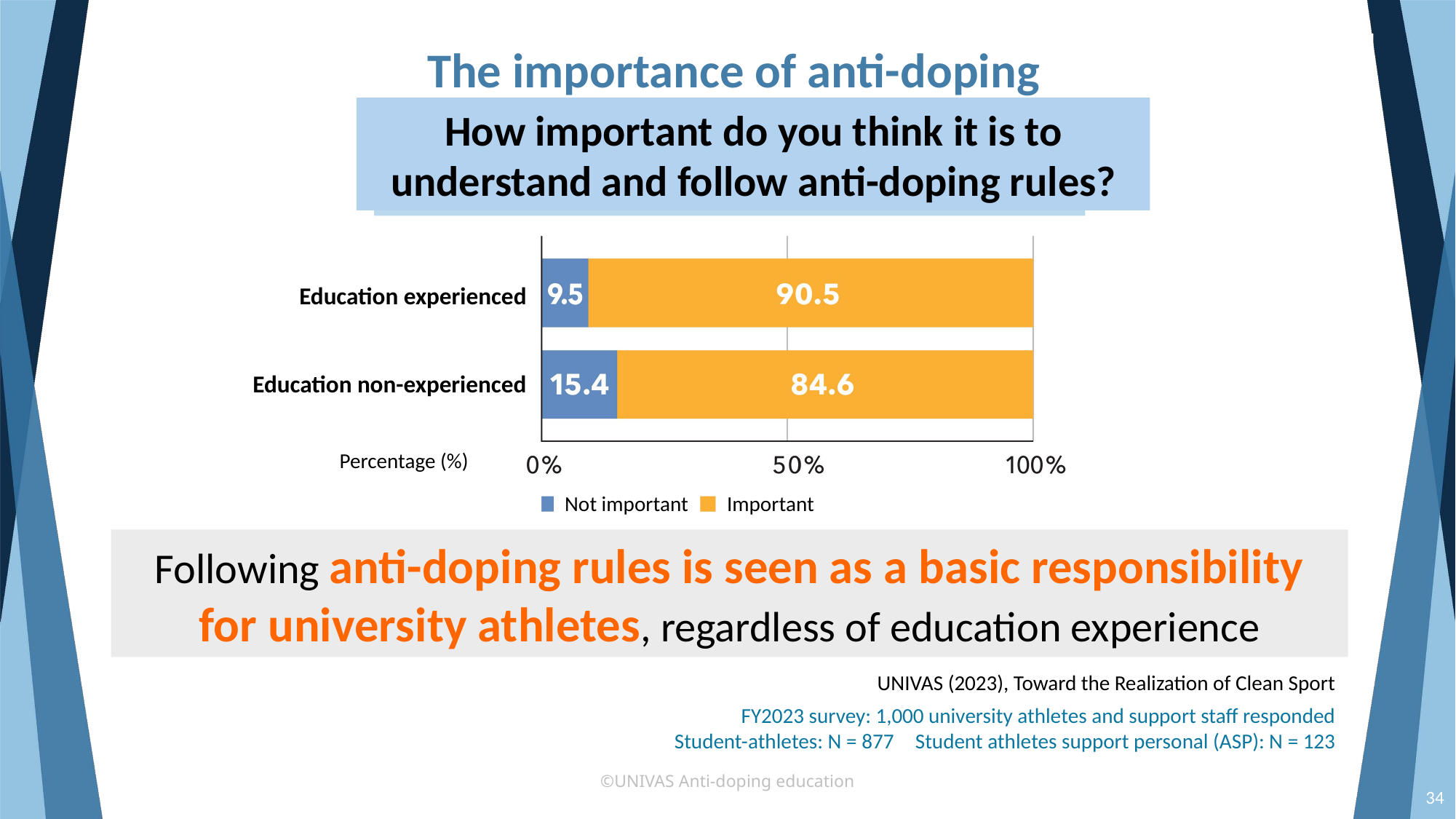

The importance of anti-doping
アンチ・ドーピングの重要性
How important do you think it is to understand and follow anti-doping rules?
Education experienced
Education non-experienced
Percentage (%)
Not important
Important
Following anti-doping rules is seen as a basic responsibility for university athletes, regardless of education experience
UNIVAS (2023), Toward the Realization of Clean Sport
FY2023 survey: 1,000 university athletes and support staff responded
Student-athletes: N = 877 Student athletes support personal (ASP): N = 123
©UNIVAS Anti-doping education
33

## Slide 35
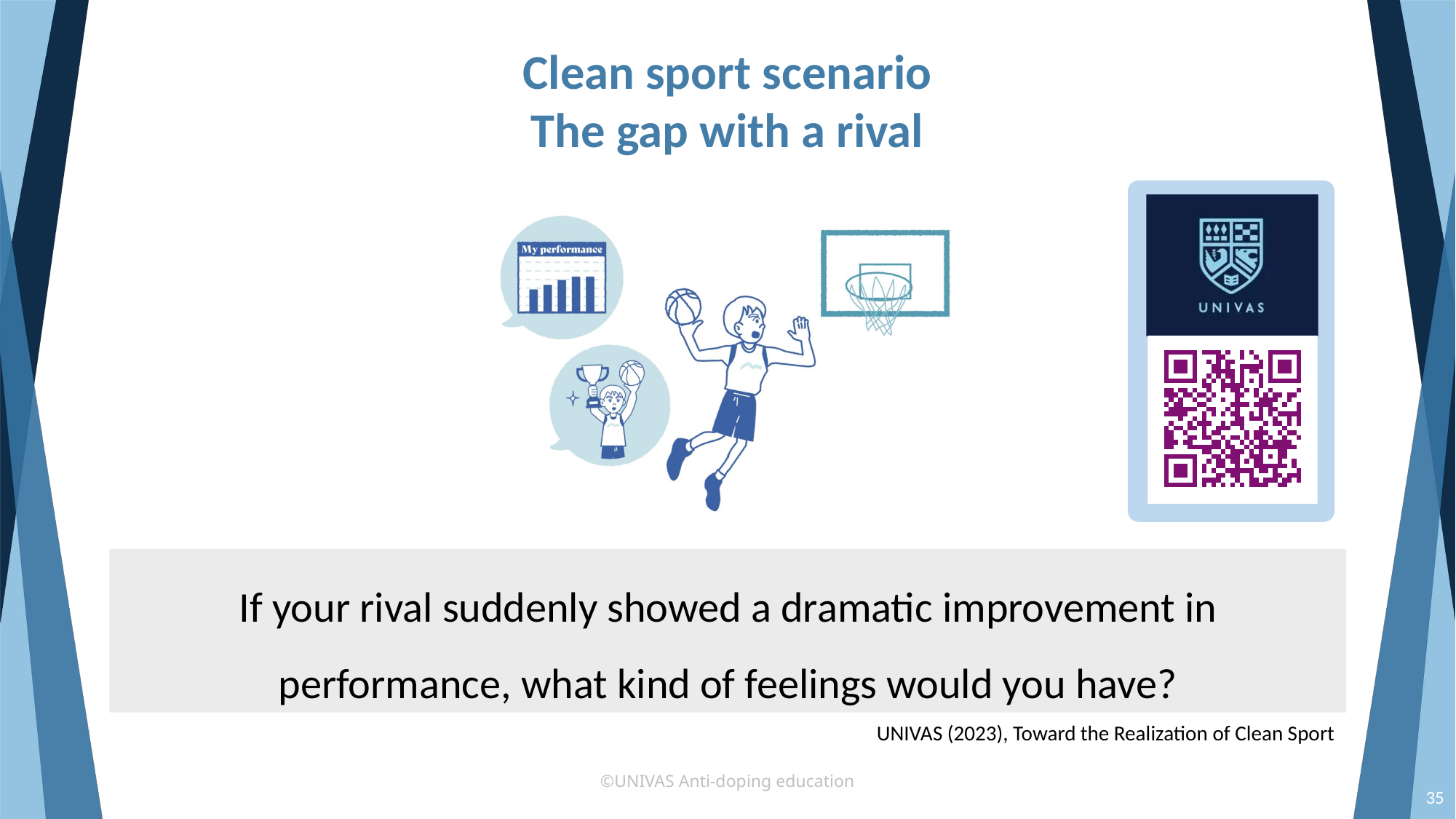

Clean sport scenario
The gap with a rival
If your rival suddenly showed a dramatic improvement in performance, what kind of feelings would you have?
UNIVAS (2023), Toward the Realization of Clean Sport
©UNIVAS Anti-doping education
34

## Slide 36
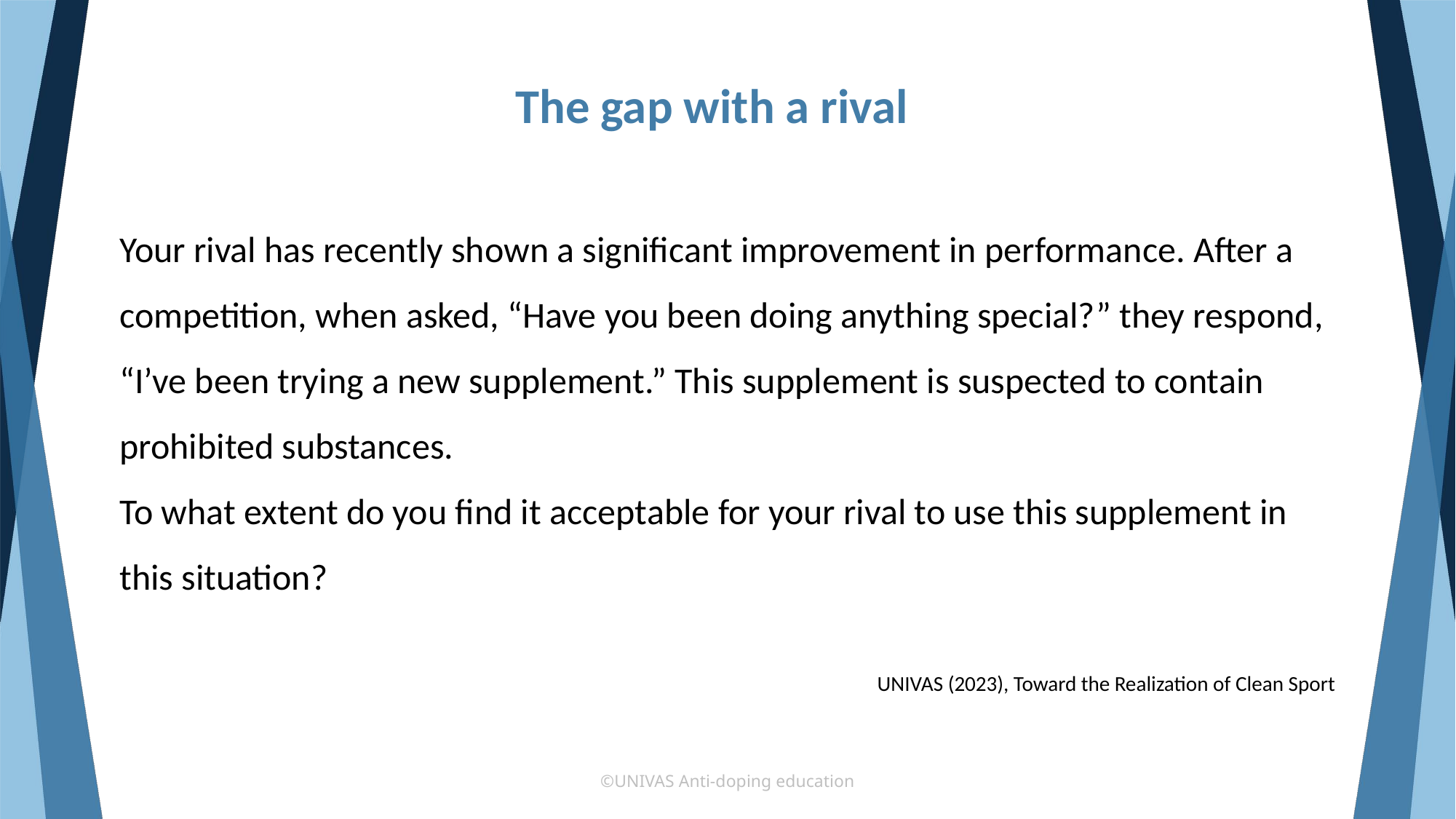

The gap with a rival
Your rival has recently shown a significant improvement in performance. After a competition, when asked, “Have you been doing anything special?” they respond, “I’ve been trying a new supplement.” This supplement is suspected to contain prohibited substances.
To what extent do you find it acceptable for your rival to use this supplement in this situation?
35
UNIVAS (2023), Toward the Realization of Clean Sport
©UNIVAS Anti-doping education

## Slide 37
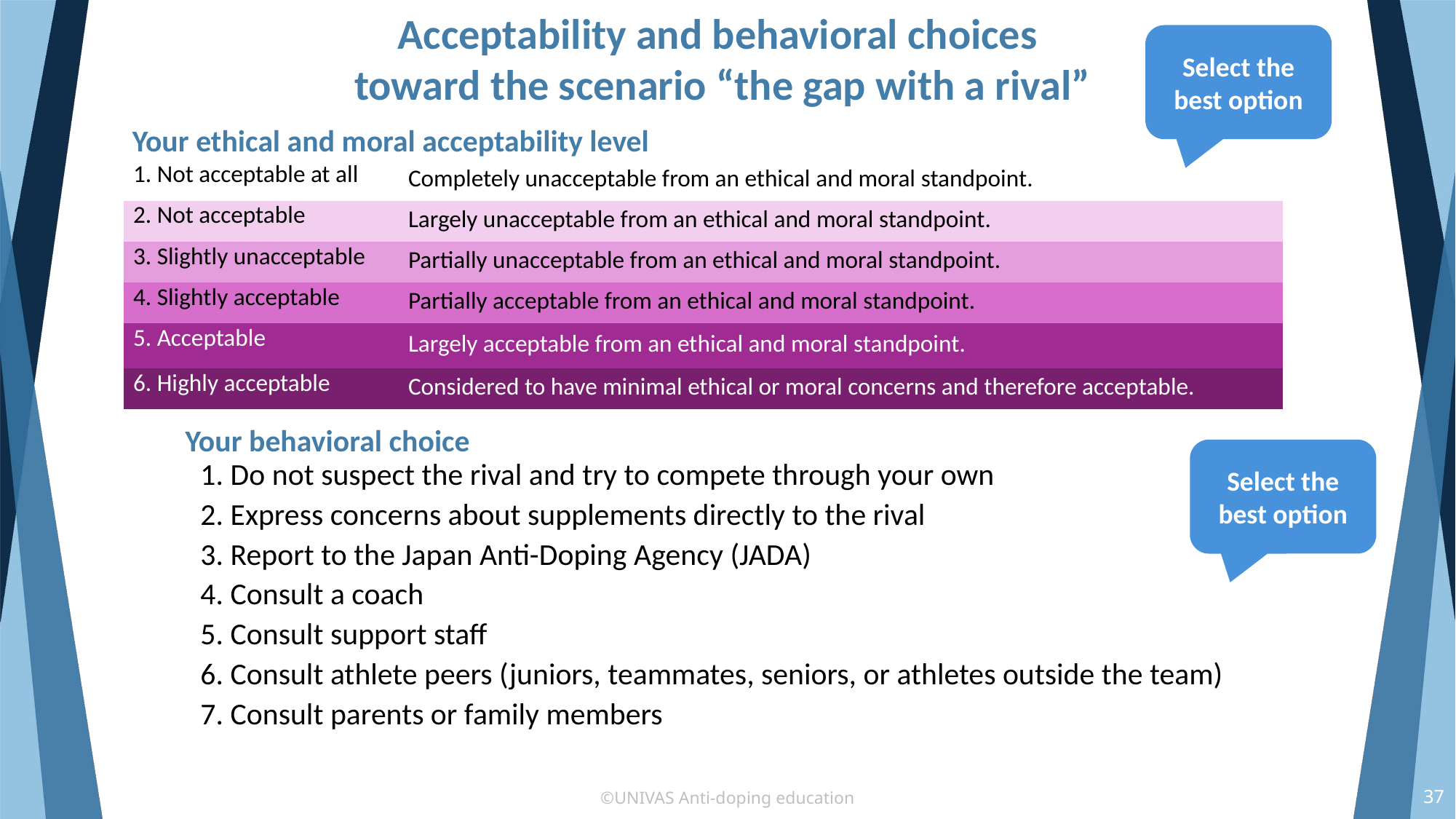

Acceptability and behavioral choices
toward the scenario “the gap with a rival”
Select the best option
Your ethical and moral acceptability level
| 1. Not acceptable at all | Completely unacceptable from an ethical and moral standpoint. |
| --- | --- |
| 2. Not acceptable | Largely unacceptable from an ethical and moral standpoint. |
| 3. Slightly unacceptable | Partially unacceptable from an ethical and moral standpoint. |
| 4. Slightly acceptable | Partially acceptable from an ethical and moral standpoint. |
| 5. Acceptable | Largely acceptable from an ethical and moral standpoint. |
| 6. Highly acceptable | Considered to have minimal ethical or moral concerns and therefore acceptable. |
Your behavioral choice
Select the best option
| 1. Do not suspect the rival and try to compete through your own |
| --- |
| 2. Express concerns about supplements directly to the rival |
| 3. Report to the Japan Anti-Doping Agency (JADA) |
| 4. Consult a coach |
| 5. Consult support staff |
| 6. Consult athlete peers (juniors, teammates, seniors, or athletes outside the team) |
| 7. Consult parents or family members |
©UNIVAS Anti-doping education
36

## Slide 38
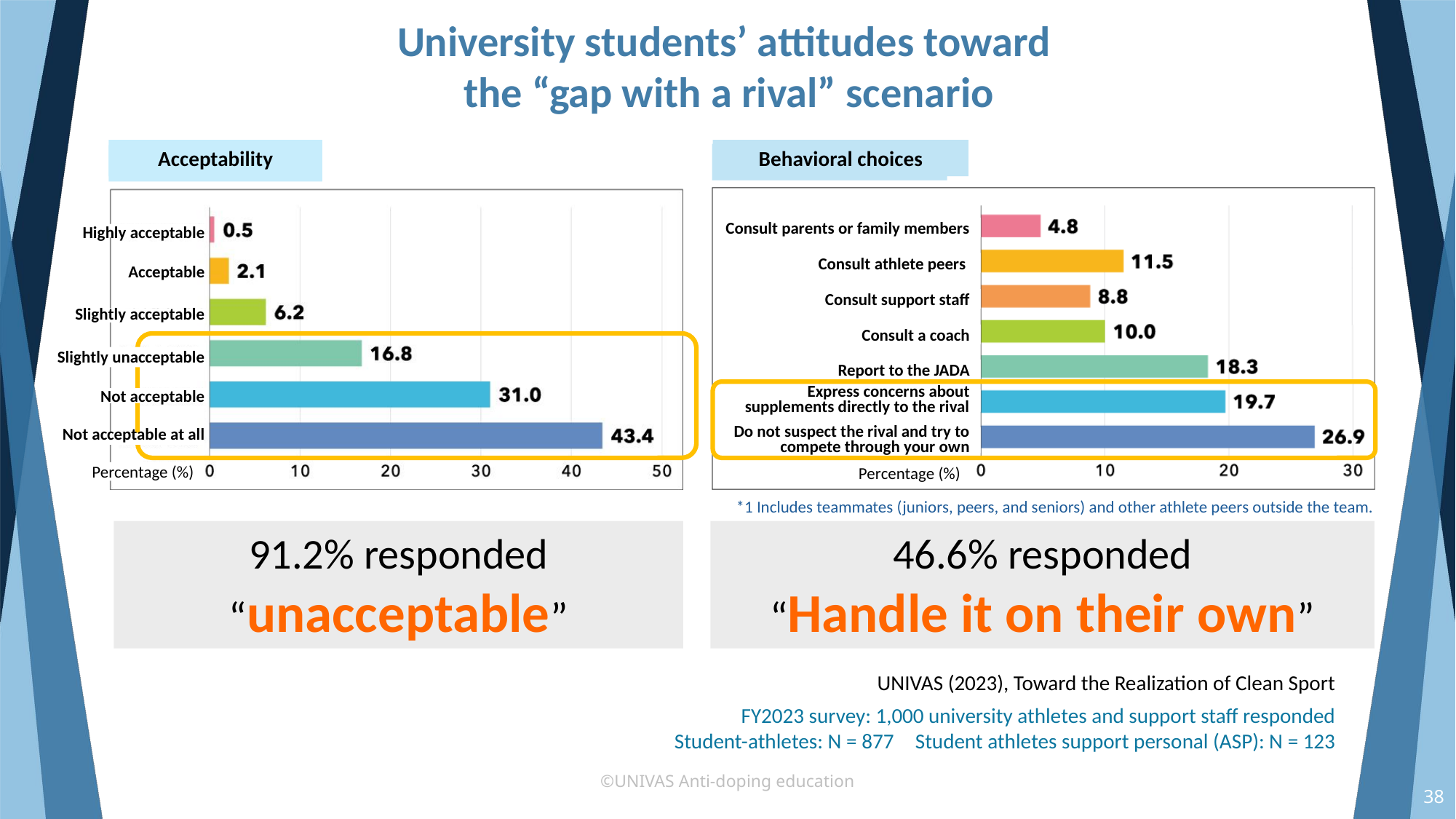

University students’ attitudes toward
the “gap with a rival” scenario
Acceptability
学生が選んだ許容度
Highly acceptable
Acceptable
Slightly acceptable
Slightly unacceptable
Not acceptable
Not acceptable at all
Percentage (%)
Behavioral choices
Consult parents or family members
Consult athlete peers
Consult support staff
Consult a coach
Report to the JADA
Express concerns about supplements directly to the rival
Do not suspect the rival and try to compete through your own
Percentage (%)
学生が選んだ行動
*1 Includes teammates (juniors, peers, and seniors) and other athlete peers outside the team.
91.2% responded
“unacceptable”
46.6% responded
“Handle it on their own”
UNIVAS (2023), Toward the Realization of Clean Sport
FY2023 survey: 1,000 university athletes and support staff responded
Student-athletes: N = 877 Student athletes support personal (ASP): N = 123
©UNIVAS Anti-doping education
37

## Slide 39
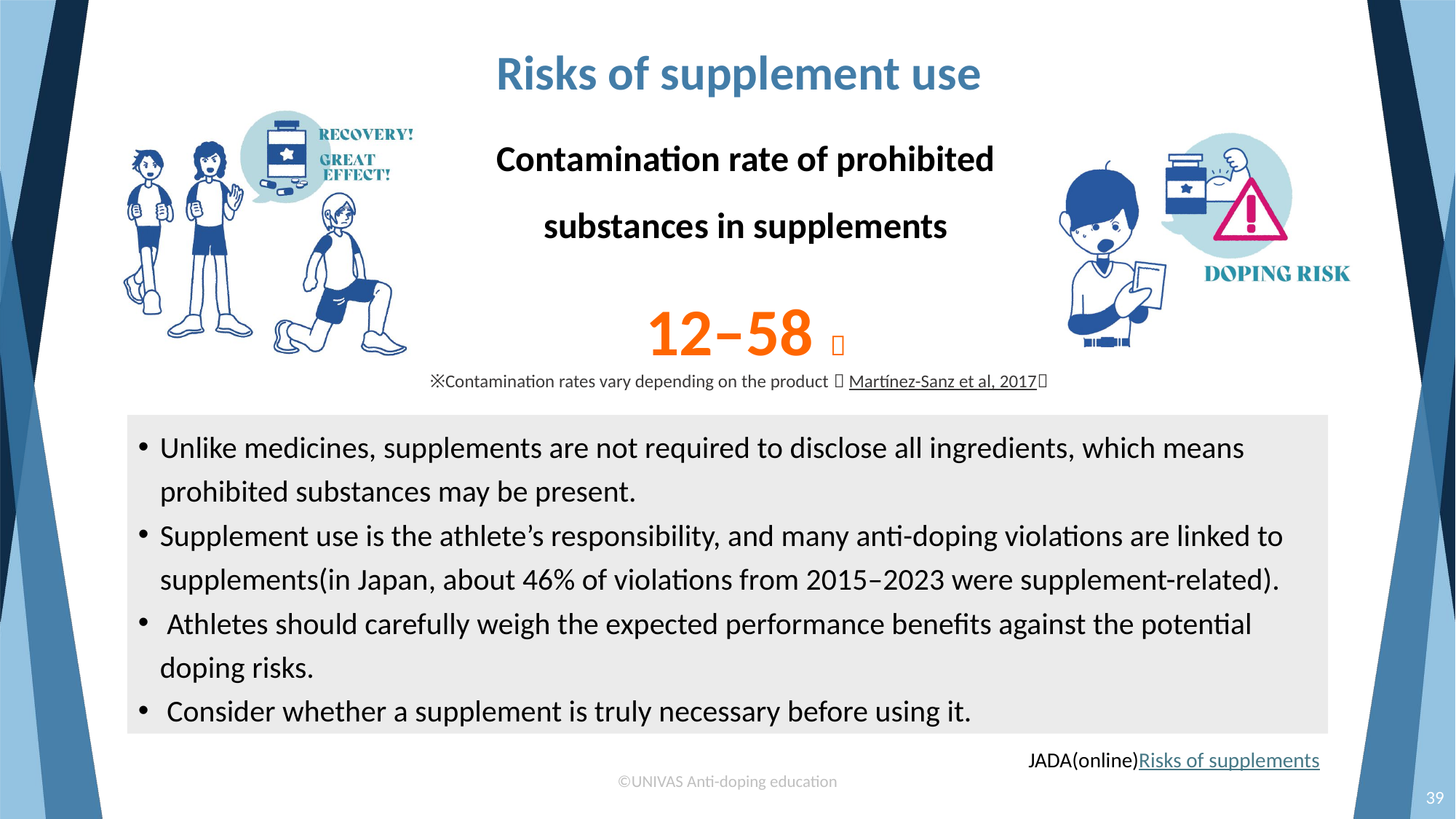

Risks of supplement use
Contamination rate of prohibited substances in supplements
12–58％
※Contamination rates vary depending on the product（Martínez-Sanz et al, 2017）
Unlike medicines, supplements are not required to disclose all ingredients, which means prohibited substances may be present.
Supplement use is the athlete’s responsibility, and many anti-doping violations are linked to supplements(in Japan, about 46% of violations from 2015–2023 were supplement-related).
 Athletes should carefully weigh the expected performance benefits against the potential doping risks.
 Consider whether a supplement is truly necessary before using it.
JADA(online)Risks of supplements
©UNIVAS Anti-doping education
38

## Slide 40
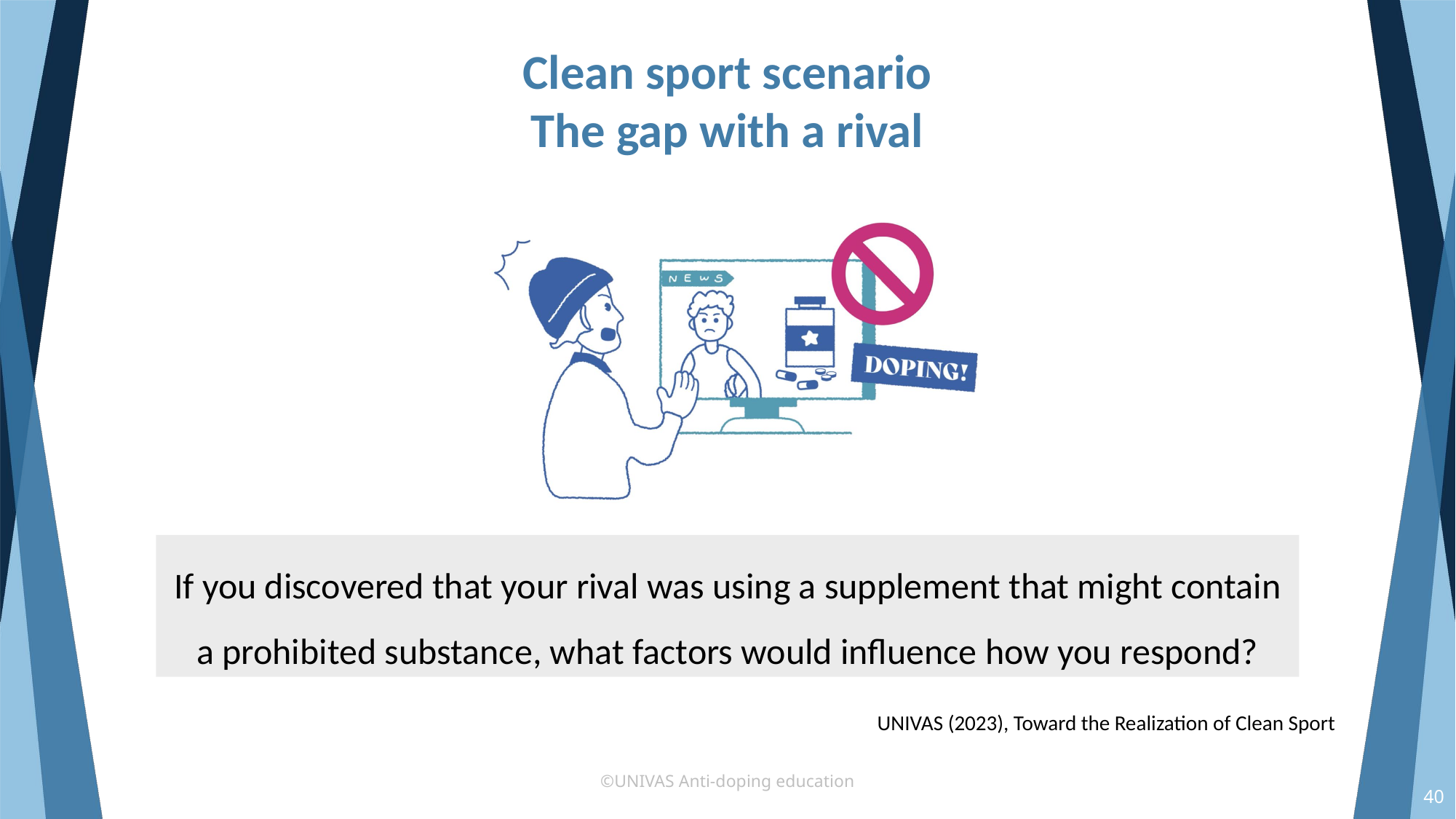

Clean sport scenario
The gap with a rival
If you discovered that your rival was using a supplement that might contain a prohibited substance, what factors would influence how you respond?
UNIVAS (2023), Toward the Realization of Clean Sport
©UNIVAS Anti-doping education
39

## Slide 41
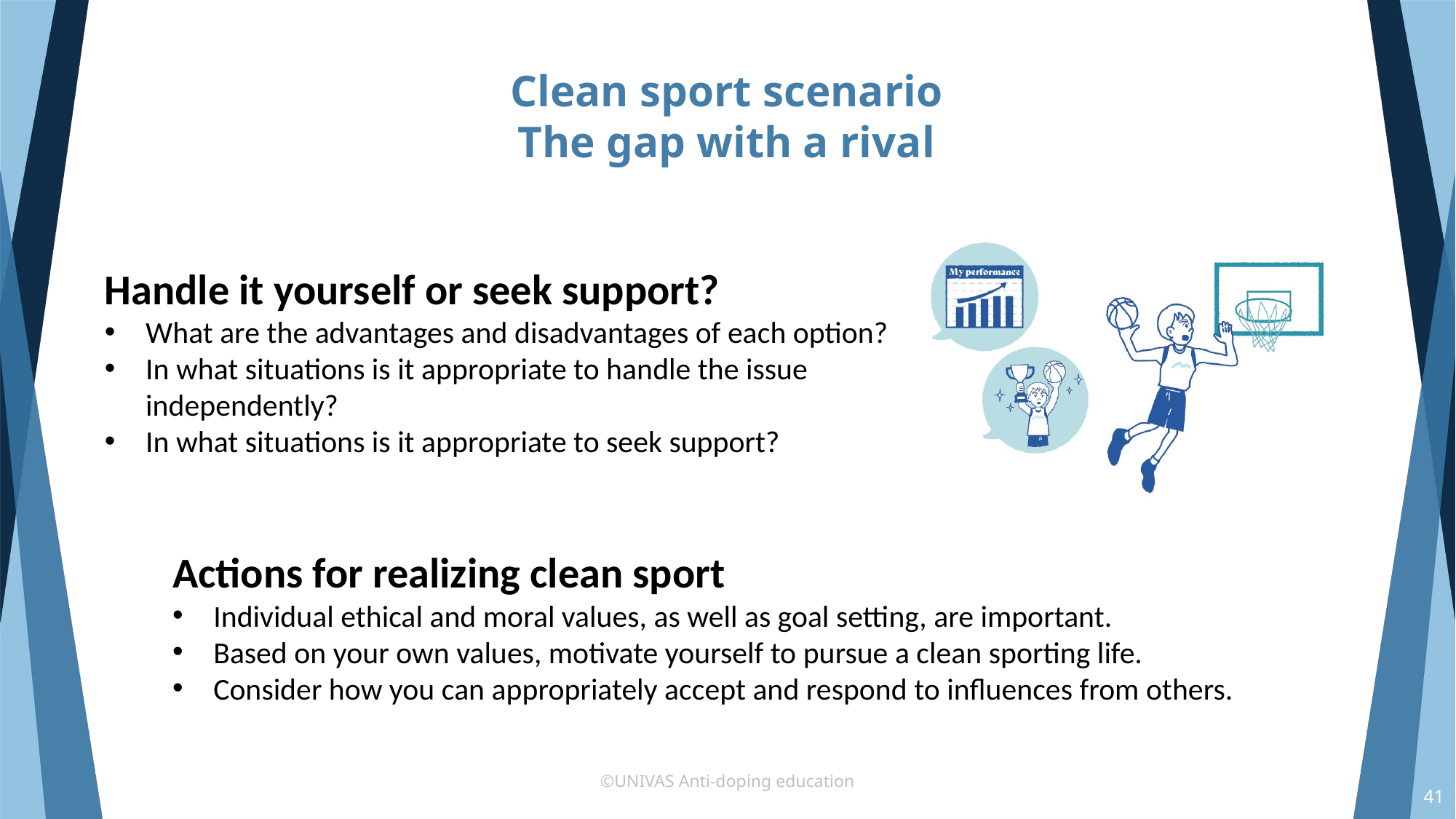

Clean sport scenario
The gap with a rival
Handle it yourself or seek support?
What are the advantages and disadvantages of each option?
In what situations is it appropriate to handle the issue independently?
In what situations is it appropriate to seek support?
Actions for realizing clean sport
Individual ethical and moral values, as well as goal setting, are important.
Based on your own values, motivate yourself to pursue a clean sporting life.
Consider how you can appropriately accept and respond to influences from others.
©UNIVAS Anti-doping education
40

## Slide 42
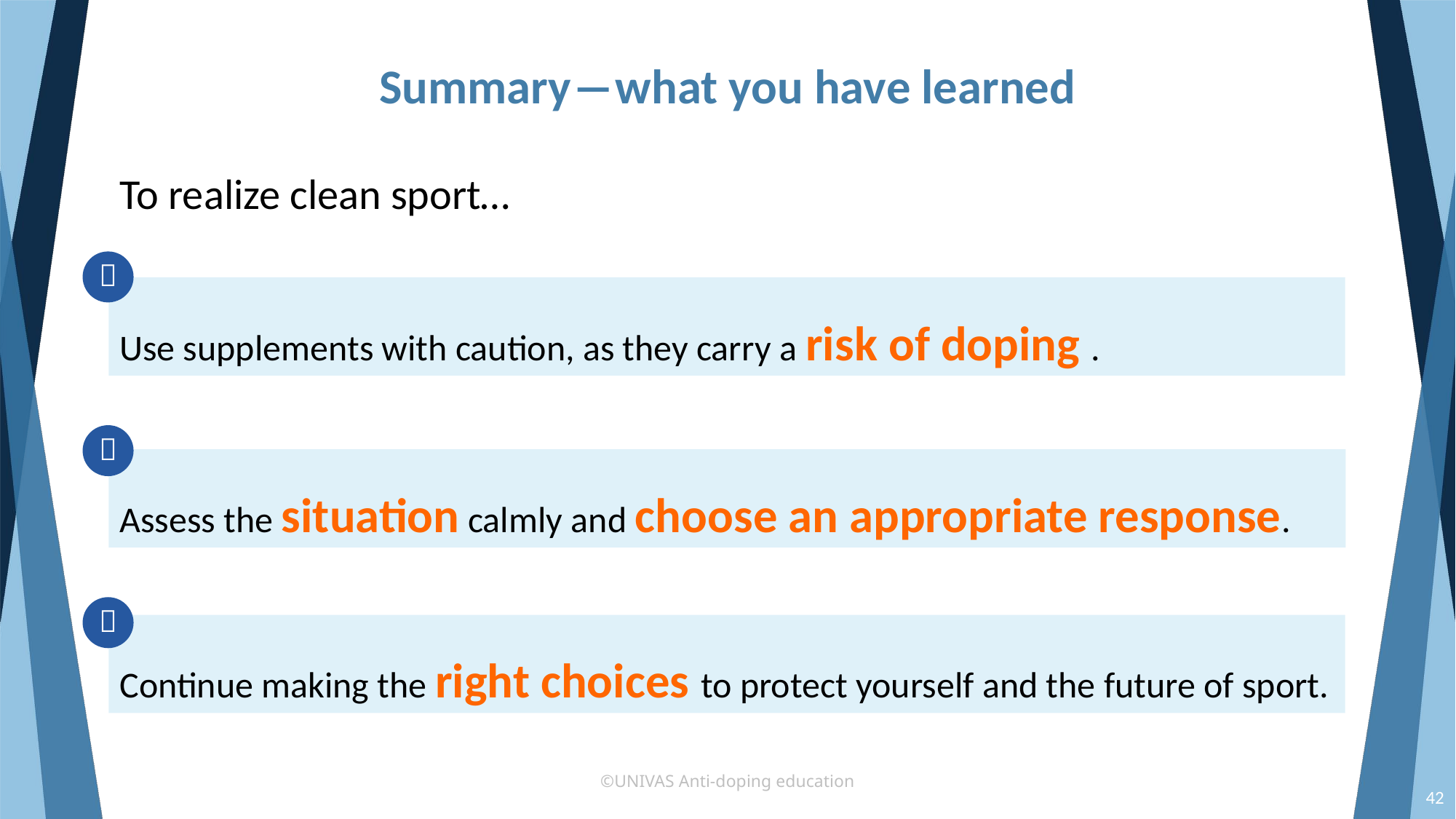

Summary―what you have learned
To realize clean sport…
１
Use supplements with caution, as they carry a risk of doping .
２
Assess the situation calmly and choose an appropriate response.
３
Continue making the right choices to protect yourself and the future of sport.
©UNIVAS Anti-doping education
41
